# Supplementary material for: PHENO-RAG: An artificial intelligence tool for guideline-informed management decisions in hepatocellular carcinoma
Source: JHEP Rep. 2025 Dec 19;8(4):101715. doi: 10.1016/j.jhepr.2025.101715 (PMC12995883; doi:10.1016/j.jhepr.2025.101715)

PHENO-RAG: An artificial intelligence tool for guideline-informed management decisions in hepatocellular carcinoma

Ciro Celsa, Mauro Giuffrè, Gabriele Di Maria, Salvatore Gruttadauria, Ugo Palazzo, Roberto Miraglia, Luigi Maruzzelli, Duilio Pagano, Roberto Cannella, Federico Midiri, Roberta Ciccia, Mauro Salvato, Alessandro Grova, Sofia Rao, Gaetano Giusino, Alessio Quartararo, Guido Cusimano, Alba Sparacino, Valeria Gaudioso, Valeria Genovese, Rosangela Montenegro, Claudia La Mantia, Francesco Mercurio, Simone Kresevic, Milos Ajcevic, Giuseppe Cabibbo, Giansalvo Cirrincione, Calogero Cammà

Table of contents

Definition of severe extrahepatic comorbidities……………………………..…………… 2

Criteria for MDT discussion…………………………………………………….……………2

Table S1………………………………………………………………………………...….3

Composition of 100 cases used for the validation of clinical concept extraction approach………………..……………………………………………………..…………5

Table S2…………………………………………………………………………………….5

Table S3…………………………………………………………………………………….6

Prompt list for patient phenotyping…………………………………………………………7

Prompt list for treatment allocation……………………………………………………..….17

Prompt list for case complexity determination…………………………………………...24

Fig. S1……………………………………….………………………………….…………31

Fig. S2…………………………………………………………………..………………32

Definition of severe extrahepatic comorbidities
Presence of at least one of the following: history of myocardial infarction, heart failure, peripheral vascular disease, stroke/ transient ischaemic attack (TIA), Chronic obstructive pulmonary disease (COPD), diabetes with end-organ damage, chronic kidney disease stage 4-5, active solid tumor, active leukemia/lymphoma, or Acquired Immune Deficiency Syndrome (AIDS).

Criteria for MDT discussion
PATIENTS REQUIRING MDT DISCUSSION:
1. Transplant candidates:
· Within Milan criteria
· Beyond Milan but within expanded criteria
· Downstaging candidates
2. Surgical resection candidates:
· Child-Pugh A with technically resectable tumor
· Selected Child-Pugh B or clinically significant portal hypertension with preserved liver function
3. Multiple treatment options available:
· Choice between resection vs. transplant
· Choice between ablation vs. resection
· Choice between resection vs intraarterial treatments
· Choice between ablation vs intraarterial treatments
· Bridge therapy selection for transplant candidates
4. Complex cases requiring careful evaluation:
· Borderline fitness for specific treatments
· Challenging tumor location
· Technical complexity
· Significant patient preferences affecting choice
· Relevant extrahepatic comorbidities impacting treatment selection

PATIENTS NOT REQUIRING MDT DISCUSSION:
1. Decompensated cirrhosis without transplant candidacy due to:
· Elderly age
· Severe extrahepatic comorbidities
· Tumor burden exceeding transplant criteria
2. Advanced HCC with:
· Extrahepatic spread
· Main portal vein invasion (Vp4)
· Clear systemic therapy indication

Table S1: Regex Dictionary for Phenotype Extraction.

Pattern Regex Alphafetoprotein:

```
Pattern_AFP =
r"(?i)(AFP|alfafetoproteina|alfa?feto(?:proteina)?|alfafeoproteina)\s*[=:]?\s*(\d+(?:[,.]\d+)?)"
```

Pattern Regex Comorbidities:

```
Pattern_Comorbidities = (
    r"(?i)(infarto|ischemia\s+miocardica|cardiopatia\s+ischemica|"
    r"scompenso\s+cardiaco|insufficienza\s+cardiaca|"
    r"vascolare\s+periferica|arteriopatia\s+periferica|"
    r"ictus|TIA|attacco\s+ischemico|"
    r"BPCO|broncopneumopatia\s+cronica\s+ostruttiva|enfisema|bronchite\s+cronica|"

r"diabete.*?(organo|complicanze)|retinopatia\s+diabetica|neuropatia\s+diabetica|nefropatia\s+diabetica
|"
    r"insufficienza\s+renale.*?(stadio\s*4|stadio\s*5)|uremia|IRC\s+avanzata|"
    r"tumore\s+solido\s+attivo|neoplasia|carcinoma|metastasi|"
    r"leucemia|linfoma|mieloma|AIDS|HIV)"
)
```

Pattern Regex Metastases:

```
Pattern_Metastases_Negation = (
r"(?i)(assenza\s+di|non\s+presenta|esclusione\s+di|non\s+si\s+osservano|"
r"non\s+sono\s+presenti|priva\s+di|assenti|negativo\s+per)" )
Pattern_Regex_Metastases = (
r"(?i)(metastasi|secondarismi|localizzazioni\s+secondarie|diffusione|coinvolgimento).*?"
r"(polmon[ei]|oss[oae]|linfonod[oi]|peritoneo|pleura|surreni|reni|cervello|"
r"encefalo|midollo\s+spinale|vertebre|scheletro|torace|mediastino|"
r"extraepatic[ohea]|fuori\s+dal\s+fegato)" )
```

Pattern Regex Performance Status:

```
Pattern_Performance_Status = ( r"(?i)(PS|Performance\s+Status|[Ss]tatus\s+[Ff]unzionale|"
r"[Pp]erformance\s+[Ff]unzionale|ECOG)[\s:=-]*(\b[0-4]\b)" )
```

Pattern Regex Any Previous Treatment:

```
Pattern_Any_Previous_Treatment = (
r"(?i)(trapianto|chirurgia|resezion[ei]|TACE|ablazione|termoablazione|"
r"alcolizzazione|PEI|etanolo|alcol|TAE|TARE|sorafenib|regorafenib|"
r"lenvatinib|atezolizumab|bevacizumab|terapia\s+sistemica|radioterapia|"
r"chemioembolizzazione|embolizzazione|radioembolizzazione|immunoterapia|"
r"trattamento\s+chirurgico|intervento\s+chirurgico|terapia\s+locoregionale|"
r"chemioterapia|terapia\s+target|radio\s*frequenza|RF|microonde|MW)" )
```

Pattern Regex Complete Response Last Treatment:

```
Pattern_Regex_Complete_Response_Last_Treatment = (
    r"(?i)(trapianto|chirurgia|resezion[ei]|TACE|ablazione|termoablazione|"
    r"alcolizzazione|PEI|etanolo|alcol|TAE|TARE|sorafenib|regorafenib|"
    r"lenvatinib|atezolizumab|bevacizumab|terapia\s+sistemica|radioterapia|"
    r"chemioembolizzazione|embolizzazione|radioembolizzazione|immunoterapia|"
    r"trattamento\s+chirurgico|intervento\s+chirurgico|terapia\s+locoregionale|"
    r"chemioterapia|terapia\s+target|radio\s*frequenza|RF|microonde|MW).*?"
    r"(?:.*?(?:\b\d{1,2}[\/-]\d{1,2}[\/-](?:19|20)\d{2}\b|\b(?:19|20)\d{2}\b))?.*?"
    r"(risposta\s+completa|CR|complete\s+response|assenza\s+di\s+recidiva|"
    r"no\s+recidiva\s+di\s+malattia|assenza\s+di\s+progressione|"
    r"assenza\s+di\s+malattia|malattia\s+non\s+rilevabile|"
    r"remissione\s+completa|nessuna\s+progressione)"
  )
```

Regex Child-Pugh:

```
Pattern_Regex_Child-Pugh = [
    r"(?i)(?:Child[-\s]?Pugh|CP)[-\s]*(?:score|punteggio|classe)?[-\s]*(\b[5-9]|1[0-5]\b)",
    r"(?i)(\b[5-9]|1[0-5]\b)[-\s]*(?:points?|punti)?[-\s]*(?:Child[-\s]?Pugh|CP)",
    # Pattern per classe A/B/C con punteggio
    r"(?i)(?:Child[-\s]?Pugh|CP)[-\s]*(?:classe|class)?[-\s]*[ABC][-\s]*\((\b[5-9]|1[0-5]\b)\)",
    # Pattern per punteggio seguito da classe
    r"(?i)(?:punteggio|score)[-\s]*(?:Child[-\s]?Pugh|CP)[-\s]*(?:di)?[-\s]*(\b[5-9]|1[0-5]\b)"
]
```

Regex Esophageal Varices:

```
Pattern_Esophageal_Varices_Negation = (
    r"(?i)(non\s+sono\s+presenti|assenza\s+di|non\s+si\s+evidenziano|"
    r"non\s+si\s+rilevano|non\s+si\s+osservano|assenti|negative\s+per)"
)
Pattern_Esophageal_Varices_Presence = (
    r"(?i)(varic[ei]\s+esofage[ea]|varic[ei]\s+gastro-?esofage[ea])"
    r"(?:\s+(?:di\s+)?(?:F[1-3]|[Gg]rado\s*[1-4]|[Pp]iccolo\s+[Cc]alibro|"
    r"[Mm]edio\s+[Cc]alibro|[Gg]rosso\s+[Cc]alibro))?"
)
```

Regex Nodule Dimensions:

```
Pattern_Nodule_Dimension = (
    r"(?i)(nodulo|neoformazione|lesione|massa|formazione|HCC|lesioni\s+focali|"
    r"monofocale|S[1-8]|S4[ab]|tumefazione).*?"
    r"(?:di\s+)?(\d+(?:\.\d+)?(?:\s*[x×]\s*\d+(?:\.\d+)?)?)\s*(cm|mm)"
)
```

Regex Portal_Vein_Invasion:

```
Pattern_Portal_Vein_Negation = re.compile(
    r"\b(assenza\s+di|no|non|assenza|non\s+evidenza\s+di|no\s+evidenza|"
    r"non\s+si\s+osservano|no\s+segni\s+di|non\s+evidenti|senza|privo\s+di|"
    r"non\s+trombosi|non\s+invasione|non\s+più\s+evidente|"
    r"non\s+apprezz\w+|non\s+rilevat[oa]|assenza\s+di\s+recidiva)\b",
    re.IGNORECASE
)
Pattern_Portal_Vein_Normality = re.compile(
    r"\b(pervio|perviet[àa]|nella\s+norma|regolare|normale|invariat[oa])\b",
    re.IGNORECASE
)

Pattern_Portal_Vein_Presence = re.compile(
    r"\b(trombosi\s+portale|trombosi\s+portale\s+neoplastica|"
    r"invasione\s+vascolare|invasione\s+vena\s+porta|"
    r"invasione\s+portale|infiltrazione\s+vascolare|infiltrazione\s+portale|"
    r"infiltrante|LR-TIV|trombosi\s+(vascolare|tumorale)|"
    r"ramo\s+(destro|sinistro)\s+della\s+vena\s+porta|"
    r"ramo\s+portale\s+(destro|sinistro)|"
    r"con\s+trombosi\s+portale|con\s+invasione\s+vascolare)\b",
    re.IGNORECASE
)
```

Albumin:

```
Pattern_Albumin = [
r"(?i)(?:albumin[ae]|alb\.?)[\s:=]*(\d+(?:[.,]\d+)?)(?:\s*(?:g/[dD][lL]|g/[lL]|gr/dl|g%|gr%))",
r"(?i)(?:albumin[ae]|alb\.?)[\s:=]*(\d+(?:[.,]\d+)?)",
r"(?i)(?:esami[\s\w]+?|bioumor[\s\w]+?).*?(?:albumin[ae]|alb\.?)[\s:=]*(\d+(?:[.,]\d+)?)"
]
```

Composition of 100 cases used for the validation of clinical concept extraction approach

Among 100 cases used for validating clinical concept extraction, clinical decisions were represented by liver transplantation in 5 cases, surgical resection in 6, ablation in 6, transarterial treatments in 6, systemic therapy in 27, BSC in 24 and follow-up continuation in 26.
Case complexity was judged as low in 26 of cases, moderate in 41 and high in 33.

Table S2: Model-specific performance metrics for extraction of individual clinical concepts

Model Configuration	Metastasis	Complete Response	Comorbidities	Vascular Invasion	Varices	Previous Treatment
REGEX	0.40	0.50	0.58	0.62	0.68	0.75
Llama-3-8B	0.60	0.68	0.76	0.81	0.83	0.88
Llama-3-8B+REGEX	0.64	0.71	0.79	0.84	0.86	0.90
Llama-3-70B	0.66	0.73	0.82	0.87	0.88	0.92
Llama-3-70B+REGEX	0.72	0.79	0.86	0.90	0.91	0.95
GPT-oss-20B	0.63	0.70	0.78	0.83	0.85	0.89
GPT-oss-20B+REGEX	0.75	0.81	0.87	0.88	0.93	0.96
GPT-oss-120B	0.69	0.77	0.82	0.85	0.90	0.93
GPT-oss-120B+REGEX	0.80	0.85	0.90	0.92	0.95	0.98
Qwen-3-8B	0.58	0.65	0.73	0.79	0.85	0.87
Qwen-3-8B+REGEX	0.66	0.77	0.84	0.85	0.91	0.94
Qwen-3-80B	0.62	0.74	0.79	0.83	0.92	0.95
Qwen-3-80B+REGEX	0.70	0.80	0.85	0.88	0.93	0.96
Falcon-7B	0.52	0.60	0.63	0.65	0.71	0.75
Falcon-7B+REGEX	0.58	0.65	0.70	0.71	0.74	0.78
Falcon-40B	0.55	0.64	0.67	0.69	0.71	0.75
Falcon-40B+REGEX	0.60	0.66	0.72	0.73	0.78	0.82

Supplementary Table S2 reports the per-concept F1-scores for categorical clinical variables extracted from narrative clinical reports across all evaluated model configurations. The table includes performance for six key clinical concepts—metastatic disease, complete radiological response, severe comorbidities, vascular invasion, esophageal varices, and previous HCC treatment—representing the principal categorical features used for downstream clinical decision-support tasks. For each language-model family (Meta Llama-3, OpenAI GPT-oss, Qwen-3, and TII Falcon), both base models and their corresponding REGEX-augmented variants are presented alongside the standalone REGEX pipeline.

Table S3: Model-specific performance metrics for extraction of numerical parameters.

Model Configuration	Nodule Diameter	MELD	AFP	ECOG-PS	Child-Pugh	Albumin
REGEX	0.65	0.74	0.95	0.87	0.82	0.89
Llama-3-8B	0.60	0.67	0.90	0.81	0.77	0.86
Llama-3-8B+REGEX	0.63	0.71	0.90	0.84	0.79	0.85
Llama-3-70B	0.70	0.77	0.96	0.89	0.85	0.92
Llama-3-70B+REGEX	0.72	0.80	0.97	0.90	0.87	0.92
GPT-oss-20B	0.65	0.73	0.88	0.80	0.78	0.84
GPT-oss-20B+REGEX	0.66	0.71	0.91	0.85	0.80	0.87
GPT-oss-120B	0.75	0.83	0.98	0.90	0.87	0.95
GPT-oss-120B+REGEX	0.78	0.85	0.99	0.93	0.90	0.94
Qwen-3-8B	0.55	0.65	0.80	0.70	0.68	0.76
Qwen-3-8B+REGEX	0.58	0.69	0.88	0.79	0.75	0.83
Qwen-3-80B	0.57	0.65	0.86	0.76	0.72	0.81
Qwen-3-80B+REGEX	0.60	0.70	0.89	0.82	0.77	0.83
Falcon-7B	0.58	0.65	0.78	0.69	0.67	0.73
Falcon-7B+REGEX	0.59	0.64	0.80	0.72	0.70	0.75
Falcon-40B	0.62	0.74	0.89	0.82	0.79	0.83
Falcon-40B+REGEX	0.68	0.74	0.89	0.82	0.79	0.83

Supplementary Table S3 presents the intraclass correlation coefficients (ICC) for six numerical parameters extracted from clinical reports—nodule maximum diameter, MELD score, AFP levels, ECOG Performance Status, Child-Pugh score, and serum albumin. These metrics quantify agreement between automated extraction systems and human-annotated reference values, reflecting the accuracy of continuous variable retrieval.

Prompt List for Patient Phenotyping

Prompt Extraction Alpha-fetoprotein:

This prompt consists of three sections: “Instruction”, “Examples”, and “Input”. The “Instruction” section contains information on your task, the “Examples” section contains 10 examples of clinical notes from patients with Hepatocellular Carcinoma (HCC) and the relevant clinical note extracted; while the “Input” section contains a new clinical note from a patient with HCC, for which you need to extract the relevant information based on the provided “Instruction” and “Examples”. All the notes are in their original language (i.e., Italian), thus some word variations reported in the Instruction section are based on the original language of the clinical note.

Section 1: Instruction
Read the following clinical report and extract the numerical value of **Alpha-Fetoprotein (AFP)**. The value may be preceded or followed by the terms **AFP**, **alpha-feto**, **alfafetoproteina**, **alfa-fetoproteina**, or similar variations.
- The AFP value may be written in decimal format using either a **dot (4.4) or a comma (4,4)**. Both formats are considered equivalent.
- The AFP value is usually (but not exclusively) found in a section that begins with "Laboratory tests" or "Biochemical exams."
- **If the AFP value is present, respond ONLY with the number in dot-decimal format.**
- **If the AFP value is not reported, respond with `"Not reported"`.**
- **Do not include any additional text in your response.**

Section 2: Examples
Example N:
Clinical Report:
"{Example Clinical Note}"
Response: {Example of Value Extraction}

Section 3: Input
Clinical Report:
"{Patient_Data}"
Response:

Prompt Extraction Severe Comorbidities:

This prompt consists of three sections: “Instruction”, “Examples”, and “Input”. The “Instruction” section contains information on your task, the “Examples” section contains 10 examples of clinical notes from patients with Hepatocellular Carcinoma (HCC) and the relevant clinical note extracted; while the “Input” section contains a new clinical note from a patient with HCC, for which you need to extract the relevant information based on the provided “Instruction” and “Examples”. All the notes are in their original language (i.e., Italian), thus some word variations reported in the Instruction section are based on the original language of the clinical note.

Section 1: Instruction

Read the following clinical report and determine whether at least **one** of the following comorbidities is present. If **any** of the listed comorbidities are found, respond with `1`. Otherwise, respond with `0`. #### **Targeted Comorbidities:** - **History of myocardial infarction** (e.g., *infarto*, *ischemia miocardica*, *cardiopatia ischemica*) - **Heart failure** (e.g., *scompenso cardiaco*, *insufficienza cardiaca*) - **Peripheral vascular disease** (e.g., *vascolare periferica*, *arteriopatia periferica*) - **Stroke or transient ischemic attack (TIA)** (e.g., *ictus*, *TIA*, *attacco ischemico transitorio*) - **Chronic obstructive pulmonary disease (COPD)** (e.g., *BPCO*, *broncopneumopatia cronica ostruttiva*, *enfisema*, *bronchite cronica*) - **Diabetes with organ complications** (e.g., *diabete con complicanze d'organo*, *retinopatia diabetica*, *neuropatia diabetica*, *nefropatia diabetica*) - **Chronic kidney disease (stage 4 or 5)** (e.g., *insufficienza renale stadio 4 o 5*, *uremia*, *IRC avanzata*) - **Active solid tumor** (e.g., *tumore solido attivo*, *neoplasia*, *carcinoma*, *metastasi*) - **Active leukemia or lymphoma** (e.g., *leucemia*, *linfoma*, *mieloma*) - **AIDS/HIV** (e.g., *AIDS*, *HIV*)
Section 2: Examples
Example N:
Clinical Report:
"{Example Clinical Note}"
Response: {Example of Value Extraction}

Section 3: Input
Clinical Report:
"{Patient_Data}"
Response:

Prompt Extraction Metastases:

This prompt consists of three sections: "Instruction", "Examples", and "Input". The "Instruction" section contains information on your task, the "Examples" section contains 10 examples of clinical notes from patients with Hepatocellular Carcinoma (HCC) and the relevant clinical note extracted; while the "Input" section contains a new clinical note from a patient with HCC, for which you need to extract the relevant information based on the provided "Instruction" and "Examples". All the notes are in their original language (i.e., Italian), thus some word variations reported in the Instruction section are based on the original language of the clinical note.

Section 1: Instruction
Read the following clinical report and determine whether at least **one** of the following extrahepatic metastases (i.e., tumor spread beyond the liver) is present. If **any** of the listed metastatic sites are found, respond with `1`. Otherwise, respond with `0`. #### **Extrahepatic Metastases Locations:** - **Lungs** (e.g., *polmoni*, *polmone*) - **Bones** (e.g., *osso*, *ossa*, *scheletro*, *vertebre*, *midollo spinale*) - **Lymph nodes** (e.g., *linfonodi*, *linfonodale*, *mediastino*) - **Peritoneum** (e.g., *peritoneo*) - **Pleura** (e.g., *pleura*, *torace*) - **Adrenal glands** (e.g., *surreni*) - **Kidneys** (e.g., *reni*) - **Brain** (e.g., *cervello*, *encefalo*) - **Other locations beyond the liver** (e.g., *extraepatico*, *extraepatiche*, *fuori dal fegato*, *extrapatico*)
Section 2: Examples

Example N:
Clinical Report:
"{Example Clinical Note}"
Response: {Example of Value Extraction}

Section 3: Input
Clinical Report:
"{Patient_Data}"
Response:

Prompt Extraction Performance Status:

This prompt consists of three sections: “Instruction”, “Examples”, and “Input”. The “Instruction” section contains information on your task, the “Examples” section contains 10 examples of clinical notes from patients with Hepatocellular Carcinoma (HCC) and the relevant clinical note extracted; while the “Input” section contains a new clinical note from a patient with HCC, for which you need to extract the relevant information based on the provided “Instruction” and “Examples”. All the notes are in their original language (i.e., Italian), thus some word variations reported in the Instruction section are based on the original language of the clinical note.

Section 1: Instruction
Read the following clinical report and extract the **numerical value** of the **Performance Status (PS)**. The PS is an **integer between 0 and 4** that represents the patient's functional capacity. - The PS value may be written as **"PS"**, **"Performance Status"**, **"status funzionale"**, **"performance funzionale"**, or similar terms. - The PS value is always an **integer (0, 1, 2, 3, or 4)**. - **If the value is present, respond ONLY with the number.** - **If the value is not reported, respond with `"Not reported"`.** - **Do not include any additional text in your response.** If a match is found, extract the **numerical value (0-4)**. Otherwise, return `"Not reported"`.

Section 2: Examples
Example N:
Clinical Report:
"{Example Clinical Note}"
Response: {Example of Value Extraction}

Section 3: Input
Clinical Report:
"{Patient_Data}"
Response:

Prompt Extraction Portal Vein Invasion:

This prompt consists of three sections: “Instruction”, “Examples”, and “Input”. The “Instruction” section contains information on your task, the “Examples” section contains 10 examples of clinical notes from patients with Hepatocellular Carcinoma (HCC) and the relevant clinical note extracted; while the “Input” section contains a new clinical note from

a patient with HCC, for which you need to extract the relevant information based on the provided "Instruction" and "Examples". All the notes are in their original language (i.e., Italian), thus some word variations reported in the Instruction section are based on the original language of the clinical note.

Section 1: Instruction
Read the following clinical report and determine whether there is **clear and non-negated evidence** of **tumor invasion of the portal vein** or **portal system**.
Criteria for Identifying Positive Cases (Portal Vein Invasion Present) Consider the presence of **portal vein invasion** only if there is a clear and **non-negated** mention of **at least one** of the following conditions: 1. **Neoplastic portal vein thrombosis** (*trombosi portale neoplastica*) 2. **Tumor or vascular thrombosis of the portal vein** (*trombosi portale tumorale o vascolare*) 3. **Tumor-related vascular invasion** (*invasione vascolare riferita a processo tumorale*) 4. **Portal vein invasion** (*invasione della vena porta*) 5. **Portal invasion** (*invasione portale*) 6. **Vascular or portal infiltration** (*infiltrazione vascolare o portale*) 7. **Presence of LR-TIV** 8. **Thrombosis or invasion of the right or left branch of the portal vein** (*ramo portale destro o sinistro con trombosi/invasione*) 9. **Thrombosis or infiltration of a portal branch (right or left)**
If **any** of these terms appear **without negation**, classify the case as `"1"`.
Criteria for Identifying Negative Cases (No Portal Vein Invasion) Consider the absence of **portal vein invasion** if **any** of the following conditions are mentioned: 1. **Portal system is patent** (*sistema portale pervio*) 2. **Portal vein is patent** (*vena porta pervia*) 3. **Portal system is normal** (*sistema portale nella norma*) 4. **Patent portal system** (*pervietà del sistema portale*) 5. **Absence of portal thrombosis** (*assenza di trombosi portale*) 6. **No evidence of thrombosis** (*non evidenza di trombosi*) 7. **Absence of vascular invasion** (*assenza di invasione vascolare*) 8. **No signs of infiltration** (*no segni di infiltrazione*) 9. **No observed invasion** (*non si osserva invasione*) 10. **Vascular system is normal or regular** (*sistema vascolare regolare/normale*) 11. **Follow-up shows normal findings** (*follow-up nella norma*) 12. **No recurrence is detected** (*assenza di recidiva*) 13. **Not evident anymore** (*non più evidente*) 14. **Not appreciable** (*non apprezzabile*) 15. **Invasion not detected** (*non rilevata invasione*) 16. **Portal system unchanged and previously normal** (*sistema portale invariato e precedentemente normale*) If **any** of these terms appear in the clinical note, classify the case as `"0"`.

Important Considerations - The **mere presence of words** like *"thrombosis"* or *"invasion"* is **not sufficient** if they are preceded by **negations**. - If the report describes a **normal follow-up** or **absence of recurrence**, classify the case as `"No"`.
- **If in doubt or ambiguity exists, classify the case as `"No"`**.

Section 2: Examples
Example N:
Clinical Report:
"{Example Clinical Note}"
Response: {Example of Value Extraction}

Section 3: Input
Clinical Report:
"{Patient_Data}"
Response:

Prompt Extraction Previous HCC Treatment:

This prompt consists of three sections: “Instruction”, “Examples”, and “Input”. The “Instruction” section contains information on your task, the “Examples” section contains 10 examples of clinical notes from patients with Hepatocellular Carcinoma (HCC) and the relevant clinical note extracted; while the “Input” section contains a new clinical note from a patient with HCC, for which you need to extract the relevant information based on the provided “Instruction” and “Examples”. All the notes are in their original language (i.e., Italian), thus some word variations reported in the Instruction section are based on the original language of the clinical note.

Section 1: Instruction
Read the following clinical report and determine whether at least **one** of the following **HCC treatments** is present. If **any** of the listed treatments are found, respond with `1`. Otherwise, respond with `0`. Previous treatment refers to **any therapeutic intervention** the patient has received **at any point in time** for HCC, regardless of the outcome. This includes **surgical, locoregional, systemic, and radiation-based treatments**, as well as **targeted and immunotherapies**. The presence of a treatment is confirmed if there is a direct mention of a specific intervention in the clinical note. #### **HCC Treatments:** - **Liver transplant** (e.g., *trapianto*) - **Surgery or resection** (e.g., *chirurgia*, *resezione*, *resezioni*, *trattamento chirurgico*, *intervento chirurgico*) - **Transarterial chemoembolization (TACE)** (e.g., *TACE*, *chemioembolizzazione*) - **Ablation (e.g., radiofrequency or microwave ablation)** (e.g., *ablazione*, *termoablazione*) - **Transarterial embolization (TAE)** (e.g., *TAE*, *embolizzazione*) - **Transarterial radioembolization (TARE)** (e.g., *TARE*, *radioembolizzazione*) - **Systemic therapy** (e.g., *terapia sistemica*, *chemioterapia*, *terapia target*) - **Targeted therapy** (e.g., *sorafenib*, *regorafenib*, *lenvatinib*, *atezolizumab*, *bevacizumab*, *immunoterapia*) - **Radiotherapy** (e.g., *radioterapia*)

Section 2: Examples
Example N:
Clinical Report:
"{Example Clinical Note}"
Response: {Example of Value Extraction}

Section 3: Input
Clinical Report:

"{Patient_Data}"
Response:

<u>Prompt Extraction Complete Response Last Treatment:</u>

This prompt consists of three sections: “Instruction”, “Examples”, and “Input”. The “Instruction” section contains information on your task, the “Examples” section contains 10 examples of clinical notes from patients with Hepatocellular Carcinoma (HCC) and the relevant clinical note extracted; while the “Input” section contains a new clinical note from a patient with HCC, for which you need to extract the relevant information based on the provided “Instruction” and “Examples”. All the notes are in their original language (i.e., Italian), thus some word variations reported in the Instruction section are based on the original language of the clinical note.

Section 1: Instruction
Read the following clinical report and determine whether **esophageal varices** are present. If **any** mention of esophageal varices is found, respond with `"1"`. Otherwise, respond with `"0"`. **Criteria for Identifying Esophageal Varices:** The presence of esophageal varices is confirmed if the clinical note mentions **any of the following terms**: - **Explicit mention of esophageal varices** (e.g., *varici esofagee*) - **Classification grades** associated with varices: - **F1, F2, F3**
Section 2: Examples
Example N:
Clinical Report:
"{Example Clinical Note}"
Response: {Example of Value Extraction}

Section 3: Input
Clinical Report:
"{Patient_Data}"
Response:

<u>Prompt Extraction Esophageal Varices:</u>

This prompt consists of three sections: “Instruction”, “Examples”, and “Input”. The “Instruction” section contains information on your task, the “Examples” section contains 10 examples of clinical notes from patients with Hepatocellular Carcinoma (HCC) and the relevant clinical note extracted; while the “Input” section contains a new clinical note from a patient with HCC, for which you need to extract the relevant information based on the provided “Instruction” and “Examples”. All the notes are in their original language (i.e., Italian), thus some word variations reported in the Instruction section are based on the original language of the clinical note.

Section 1: Instruction
Read the following clinical report and determine whether **esophageal varices** are present. If **any** mention of esophageal varices is found, respond with `"1"`. Otherwise, respond with `"0"`. *Criteria for Identifying Esophageal Varices:** The presence of esophageal varices is confirmed if the clinical note mentions **any of the following

terms**: - **Explicit mention of esophageal varices** (e.g., *varici esofagee*) - **Classification grades** associated with varices: - **(F)1, (F)2, (F)3**

Section 2: Examples
Example N:
Clinical Report:
"{Example Clinical Note}"
Response: {Example of Value Extraction}

Section 3: Input
Clinical Report:
"{Patient_Data}"
Response:

Prompt Extraction Nodule Dimension:

This prompt consists of three sections: “Instruction”, “Examples”, and “Input”. The “Instruction” section contains information on your task, the “Examples” section contains 10 examples of clinical notes from patients with Hepatocellular Carcinoma (HCC) and the relevant clinical note extracted; while the “Input” section contains a new clinical note from a patient with HCC, for which you need to extract the relevant information based on the provided “Instruction” and “Examples”. All the notes are in their original language (i.e., Italian), thus some word variations reported in the Instruction section are based on the original language of the clinical note.

Section 1: Instruction
Read the following clinical report and extract the **largest reported size** of a **nodule, lesion, or mass** found in the **most recent imaging study (CT, MRI, or ultrasound)**.
Criteria for Extracting Nodule Size:
1. **Identify the most recent imaging study** in the clinical note. If multiple imaging reports are present, use the **latest one**. To determine the **most recent imaging report**, extract the **latest valid year** in the format: (19|20)\d{2}.
- If **multiple sizes are mentioned**, select the **largest** one reported in the latest imaging study.
- **If a nodule size is present, return the extracted size in the original format:**
2. **Locate any mention of a nodule, lesion, mass, or tumor**, including:
- **Nodule / Neoformation / Lesion / Mass** (e.g., *nodulo*, *neoformazione*, *lesione*, *massa*, *formazione*)
- **HCC lesion** (e.g., *HCC*, *lesioni focali*, *monofocale*)
- **Segment-specific lesions** (e.g., *S1*, *S2*, *S3*, *S4*, *S4a*, *S4b*, *S5*, *S6*, *S7*, *S8*)
3. **Extract the largest reported size**, formatted as:
- **Single value** (e.g., `"5 cm"`, `"11 mm"`)
- **Multiple dimensions** (e.g., `"11 x 8.8 cm"`, `"4.5 x 3.2 mm"`)
- **Ensure the extracted value includes the unit** (`cm` or `mm`).
4. **If no nodules or lesions are found, return `"No nodule found"`**.

Section 2: Examples
Example N:
Clinical Report:
"{Example Clinical Note}"
Response: {Example of Value Extraction}

Section 3: Input
Clinical Report:
"{Patient_Data}"
Response:

Prompt Extraction Albumin:

This prompt consists of three sections: “Instruction”, “Examples”, and “Input”. The “Instruction” section contains information on your task, the “Examples” section contains 10 examples of clinical notes from patients with Hepatocellular Carcinoma (HCC) and the relevant clinical note extracted; while the “Input” section contains a new clinical note from a patient with HCC, for which you need to extract the relevant information based on the provided “Instruction” and “Examples”. All the notes are in their original language (i.e., Italian), thus some word variations reported in the Instruction section are based on the original language of the clinical note.

Section 1: Instruction
Read the following clinical report and extract the **numerical value** of **albumin** if present.
Criteria for Extracting Albumin Value: 1. **Identify the albumin value**, which may be preceded or followed by: - **Albumina, alb, albumin, albumina sierica**, or similar terms. 2. **Albumin values may be reported in decimal format** using: - **Dot (4.4) or comma (4,4)** notation (both are considered equivalent). 3. **Units are typically**: - **g/dL, g/L, gr/dL, or g%**. 4. **If the albumin value is present, respond ONLY with the number in dot-decimal format (e.g., `4.4`).** 5. **If the albumin value is not reported, respond with `"Not reported"`.** 6. **Do not include any additional text in your response.**

Section 2: Examples
Example N:
Clinical Report:
"{Example Clinical Note}"
Response: {Example of Value Extraction}

Section 3: Input
Clinical Report:
"{Patient_Data}"
Response:

Prompt Extraction MELD:

This prompt consists of three sections: “Instruction”, “Examples”, and “Input”. The “Instruction” section contains information on your task, the “Examples” section contains 10 examples of clinical notes from patients with Hepatocellular Carcinoma (HCC) and the relevant clinical note extracted; while the “Input” section contains a new clinical note from a patient with HCC, for which you need to extract the relevant information based on the provided “Instruction” and “Examples”. All the notes are in their original language (i.e., Italian), thus some word variations reported in the Instruction section are based on the original language of the clinical note.

Section 1: Instruction
Read the following clinical report and extract the **numerical value** of the **MELD score** if present.
Criteria for Extracting MELD Score: 1. **Identify the MELD value**, which may be preceded by: - **MELD, MELD score, punteggio MELD, MELD-Na**, or similar terms. 2. **MELD values are typically integers** between **6 and 40**. 3. **If the MELD value is present, respond ONLY with the number.** 4. **If the MELD value is not reported, respond with `"Not reported"`.** 5. **Do not include any additional text in your response.**

Section 2: Examples
Example N:
Clinical Report:
"{Example Clinical Note}"
Response: {Example of Value Extraction}

Section 3: Input
Clinical Report:
"{Patient_Data}"
Response:

<u>Prompt Extraction Child-Pugh:</u>
This prompt consists of three sections: “Instruction”, “Examples”, and “Input”. The “Instruction” section contains information on your task, the “Examples” section contains 10 examples of clinical notes from patients with Hepatocellular Carcinoma (HCC) and the relevant clinical note extracted; while the “Input” section contains a new clinical note from a patient with HCC, for which you need to extract the relevant information based on the provided “Instruction” and “Examples”. All the notes are in their original language (i.e., Italian), thus some word variations reported in the Instruction section are based on the original language of the clinical note.

Section 1: Instruction
Read the following clinical report and extract the **Child-Pugh classification**, including both: 1. **The class (A, B, or C)** 2. **The numerical score (if available)**.
Criteria for Extracting the Child-Pugh Classification: - The classification may appear as: - **"Class A6", "Class B7 of Child-Pugh", "Class C", "CP Class B"** - Variants such as **"Child-Pugh A", "CP class B", "Child Pugh C"** are also valid. - **If the Child-Pugh class and score are present, return both** (e.g., `"B7"`). - **If only the Child-Pugh class is

present without a score, return only the letter** (e.g., `"B"`). - **If the classification is not reported, respond with `"Not reported"`**. - **Do not include any additional text in your response.**

Section 2: Examples
Example N:
Clinical Report:
"{Example Clinical Note}"
Response: {Example of Value Extraction}

Section 3: Input
Clinical Report:
"{Patient_Data}"
Response:

Prompt List for Treatment Allocation

Zero-Shot Prompt [Unstructured Note]:

This prompt consists of two sections: “Instruction”, and “Input”. The “Instruction” section contains information on your task, while the “Input” section contains a new clinical note from a patient with HCC, for which you need to determine the most appropriate treatment based on the provided provided“Instruction”.

Section 1: Instruction
You are a **hepatology expert specializing in the treatment of Hepatocellular Carcinoma (HCC)**. Read the following unstructured clinical report (uploaded in its original language – Italian) carefully and, based on the **patient’s characteristics and disease status**, determine the **most appropriate therapeutic option** from the list below:
A. **Liver Transplantation** (`TRAPIANTO EPATICO`)
B. **Surgical Resection** (`RESEZIONE CHIRURGICA`)
C. **Ablation** (`ABLAZIONE`) → Includes:
- **Microwave Ablation (MWA)** (*ablazione a microonde*)
- **Radiofrequency Ablation (RFA)** (*ablazione con radiofrequenza*)
- **Cryoablation** (*crioablazione*)
D. **Embolization** (`EMBOLIZZAZIONE`) → Includes:
- **Transarterial Chemoembolization (TACE)** (*chemoembolizzazione transarteriosa*)
- **Transarterial Radioembolization (TARE, also called SIRT)** (*radioembolizzazione transarteriosa, SIRT*)
- **Transarterial Embolization (TAE)** (*embolizzazione transarteriosa senza chemioterapia*)
E. **Systemic Therapy** (`TERAPIA SISTEMICA`) → Includes: **Sorafenib** - **Lenvatinib** - **Regorafenib** - **Cabozantinib** - **Atezolizumab + Bevacizumab** - **Durvalumab + Tremelimumab** - **Pembrolizumab** - **Nivolumab** -
F. **Best Supportive Care** (`BEST SUPPORTIVE CARE`) → **To be selected ONLY when all other therapeutic options (curative or palliative) have been exhausted.**: The disease is **too advanced** for locoregional or has progressed on second and third line systemic therapy. The clinical approach is limited to **symptom management and supportive care** rather than curative or life-prolonging strategies.
G. **Follow-up** (`FOLLOW-UP`) → **Only if the patient has achieved complete response (CR) and no active disease is present on the latest imaging.**

Response Format: - **If the most appropriate treatment is identified, return only the name of the treatment option** from the list above. - **Do not include any additional text in your response.**

Section 2: Input
Clinical Report:
"{Patient_Data}"
Response:

Zero-Shot Prompt [Structured Note]:

This prompt consists of two sections: “Instruction”, and “Input”. The “Instruction” section contains information on your task, while the “Input” section contains a new clinical note from a patient with HCC, for which you need to determine the most appropriate treatment based on the provided provided“Instruction”.

Section 1: Instruction
You are a **hepatology expert specializing in the treatment of Hepatocellular Carcinoma (HCC)**. Read the following structured clinical report (uploaded in its original language – Italian) carefully and, based on the **patient’s characteristics and disease status**, determine the **most appropriate therapeutic option** from the list below:
A. **Liver Transplantation** (`TRAPIANTO EPATICO`)
B. **Surgical Resection** (`RESEZIONE CHIRURGICA`)
C. **Ablation** (`ABLAZIONE`) → Includes:
- **Microwave Ablation (MWA)** (*ablazione a microonde*)
- **Radiofrequency Ablation (RFA)** (*ablazione con radiofrequenza*)
- **Cryoablation** (*crioablazione*)
D. **Embolization** (`EMBOLIZZAZIONE`) → Includes:
- **Transarterial Chemoembolization (TACE)** (*chemoembolizzazione transarteriosa*)
- **Transarterial Radioembolization (TARE, also called SIRT)** (*radioembolizzazione transarteriosa, SIRT*)
- **Transarterial Embolization (TAE)** (*embolizzazione transarteriosa senza chemioterapia*)
E. **Systemic Therapy** (`TERAPIA SISTEMICA`) → Includes: **Sorafenib** - **Lenvatinib** - **Regorafenib** - **Cabozantinib** - **Atezolizumab + Bevacizumab** - **Durvalumab + Tremelimumab** - **Pembrolizumab** - **Nivolumab** -
F. **Best Supportive Care** (`BEST SUPPORTIVE CARE`) → **To be selected ONLY when all other therapeutic options (curative or palliative) have been exhausted.**: The disease is **too advanced** for locoregional or has progressed on second and third line systemic therapy. The clinical approach is limited to **symptom management and supportive care** rather than curative or life-prolonging strategies.
G. **Follow-up** (`FOLLOW-UP`) → **Only if the patient has achieved complete response (CR) and no active disease is present on the latest imaging.**

Response Format: - **If the most appropriate treatment is identified, return only the name of the treatment option** from the list above. - **Do not include any additional text in your response.**

Section 2: Input
Clinical Report:
"{Patient_Data}"
Response:

Few-Shot Prompt [Unstructured Note]:

This prompt consists of three sections: “Instruction”, “Examples”, and “Input”. The “Instruction” section contains information on your task, the “Examples” section contains 10 examples of clinical notes from patients with Hepatocellular Carcinoma (HCC) and the relevant treatment selection; while the “Input” section contains a new clinical note from a patient with HCC, for which you need to determine the most appropriate treatment based on the provided “Instruction” and “Examples.”

Section 1: Instruction
You are a **hepatology expert specializing in the treatment of Hepatocellular Carcinoma (HCC)**. Read the following unstructured clinical report (uploaded in its original language – Italian) carefully and, based on the **patient’s characteristics and disease status**, determine the **most appropriate therapeutic option** from the list below:
A. **Liver Transplantation** (`TRAPIANTO EPATICO`)
B. **Surgical Resection** (`RESEZIONE CHIRURGICA`)
C. **Ablation** (`ABLAZIONE`) → Includes:
- **Microwave Ablation (MWA)** (*ablazione a microonde*)
- **Radiofrequency Ablation (RFA)** (*ablazione con radiofrequenza*)
- **Cryoablation** (*crioablazione*)
D. **Embolization** (`EMBOLIZZAZIONE`) → Includes:
- **Transarterial Chemoembolization (TACE)** (*chemoembolizzazione transarteriosa*)
- **Transarterial Radioembolization (TARE, also called SIRT)** (*radioembolizzazione transarteriosa, SIRT*)
- **Transarterial Embolization (TAE)** (*embolizzazione transarteriosa senza chemioterapia*)
E. **Systemic Therapy** (`TERAPIA SISTEMICA`) → Includes: **Sorafenib** - **Lenvatinib** - **Regorafenib** - **Cabozantinib** - **Atezolizumab + Bevacizumab** - **Durvalumab + Tremelimumab** - **Pembrolizumab** - **Nivolumab** -
F. **Best Supportive Care** (`BEST SUPPORTIVE CARE`) → **To be selected ONLY when all other therapeutic options (curative or palliative) have been exhausted.**: The disease is **too advanced** for locoregional or has progressed on second and third line systemic therapy. The clinical approach is limited to **symptom management and supportive care** rather than curative or life-prolonging strategies.
G. **Follow-up** (`FOLLOW-UP`) → **Only if the patient has achieved complete response (CR) and no active disease is present on the latest imaging.**

Response Format: - **If the most appropriate treatment is identified, return only the name of the treatment option** from the list above. - **Do not include any additional text in your response.**

Section 2: Examples
Example N:
Clinical Report:
"{Example Clinical Note}"
Response: {Treatment Allocation}
Section 3: Input

Clinical Report:
"{Patient_Data}"
Response:

Few-Shot Prompt [Structured Note]:
This prompt consists of three sections: “Instruction”, “Examples”, and “Input”. The “Instruction” section contains information on your task, the “Examples” section contains 10 examples of clinical notes from patients with Hepatocellular Carcinoma (HCC) and the relevant treatment selection; while the “Input” section contains a new clinical note from a patient with HCC, for which you need to determine the most appropriate treatment based on the provided “Instruction” and “Examples.”

Section 1: Instruction
You are a **hepatology expert specializing in the treatment of Hepatocellular Carcinoma (HCC)**. Read the following Structured clinical report (uploaded in its original language – Italian) carefully and, based on the **patient’s characteristics and disease status**, determine the **most appropriate therapeutic option** from the list below:
A. **Liver Transplantation** (`TRAPIANTO EPATICO`)
B. **Surgical Resection** (`RESEZIONE CHIRURGICA`)
C. **Ablation** (`ABLAZIONE`) → Includes:
- **Microwave Ablation (MWA)** (*ablazione a microonde*)
- **Radiofrequency Ablation (RFA)** (*ablazione con radiofrequenza*)
- **Cryoablation** (*crioablazione*)
D. **Embolization** (`EMBOLIZZAZIONE`) → Includes:
- **Transarterial Chemoembolization (TACE)** (*chemoembolizzazione transarteriosa*)
- **Transarterial Radioembolization (TARE, also called SIRT)** (*radioembolizzazione transarteriosa, SIRT*)
- **Transarterial Embolization (TAE)** (*embolizzazione transarteriosa senza chemioterapia*)
E. **Systemic Therapy** (`TERAPIA SISTEMICA`) → Includes: **Sorafenib** - **Lenvatinib** - **Regorafenib** - **Cabozantinib** - **Atezolizumab + Bevacizumab** - **Durvalumab + Tremelimumab** - **Pembrolizumab** - **Nivolumab** -
F. **Best Supportive Care** (`BEST SUPPORTIVE CARE`) → **To be selected ONLY when all other therapeutic options (curative or palliative) have been exhausted.**: The disease is **too advanced** for locoregional or has progressed on second and third line systemic therapy. The clinical approach is limited to **symptom management and supportive care** rather than curative or life-prolonging strategies.
G. **Follow-up** (`FOLLOW-UP`) → **Only if the patient has achieved complete response (CR) and no active disease is present on the latest imaging.**

Response Format: - **If the most appropriate treatment is identified, return only the name of the treatment option** from the list above. - **Do not include any additional text in your response.**

Section 2: Examples
Example N:

Clinical Report:
"{Example Clinical Note}"
Response: {Treatment Allocation}

Section 3: Input
Clinical Report:
"{Patient_Data}"
Response:

<u>Few-Shot+RAG Prompt [Unstructured Note]:</u>

This prompt consists of four sections: “Instruction”, “Examples”, “Guidelines”, and “Input”. The “Instruction” section contains information on your task, the “Examples” section contains 10 examples of clinical notes from patients with Hepatocellular Carcinoma (HCC) and the relevant treatment selection; the “Guidelines” section provides text chunks from the latest guidelines, which contain strict recommendations that determine the correct treatment allocation - these guidelines must be used in addition to the provided examples when selecting the most appropriate treatment; while the “Input” section contains a new clinical note from a patient with HCC, for which you need to determine the most appropriate treatment based on the provided “Instruction”, “Examples”, and “Guidelines”.

Section 1: Instruction
You are a **hepatology expert specializing in the treatment of Hepatocellular Carcinoma (HCC)**. Read the following unstructured clinical report (uploaded in its original language – Italian) carefully and, based on the **patient’s characteristics and disease status**, determine the **most appropriate therapeutic option** from the list below:
A. **Liver Transplantation** (`TRAPIANTO EPATICO`)
B. **Surgical Resection** (`RESEZIONE CHIRURGICA`)
C. **Ablation** (`ABLAZIONE`) → Includes:
- **Microwave Ablation (MWA)** (*ablazione a microonde*)
- **Radiofrequency Ablation (RFA)** (*ablazione con radiofrequenza*)
- **Cryoablation** (*crioablazione*)
D. **Embolization** (`EMBOLIZZAZIONE`) → Includes:
- **Transarterial Chemoembolization (TACE)** (*chemoembolizzazione transarteriosa*)
- **Transarterial Radioembolization (TARE, also called SIRT)** (*radioembolizzazione transarteriosa, SIRT*)
- **Transarterial Embolization (TAE)** (*embolizzazione transarteriosa senza chemioterapia*)
E. **Systemic Therapy** (`TERAPIA SISTEMICA`) → Includes: **Sorafenib** - **Lenvatinib** - **Regorafenib** - **Cabozantinib** - **Atezolizumab + Bevacizumab** - **Durvalumab + Tremelimumab** - **Pembrolizumab** - **Nivolumab** -
F. **Best Supportive Care** (`BEST SUPPORTIVE CARE`) → **To be selected ONLY when all other therapeutic options (curative or palliative) have been exhausted.**: The disease is **too advanced** for locoregional or has progressed on second and third line

systemic therapy. The clinical approach is limited to **symptom management and supportive care** rather than curative or life-prolonging strategies.
G. **Follow-up** (`FOLLOW-UP`) → **Only if the patient has achieved complete response (CR) and no active disease is present on the latest imaging.**

Response Format: - **If the most appropriate treatment is identified, return only the name of the treatment option** from the list above. - **Do not include any additional text in your response.**

Section 2: Examples
Example N:
Clinical Report:
"{Example Clinical Note}"
Response: {Treatment Allocation}

Section 3: Guidelines
The following text chunks are extracted from the latest guidelines related to HCC treatment and provide strict recommendations for treatment allocation. These must be used in conjunction with the examples above to determine the correct treatment.

Section 4: Input
Clinical Report:
"{Patient_Data}"
Response:

Few-Shot+RAG Prompt [Structured Note]:
This prompt consists of four sections: “Instruction”, “Examples”, “Guidelines”, and “Input”. The “Instruction” section contains information on your task, the “Examples” section contains 10 examples of clinical notes from patients with Hepatocellular Carcinoma (HCC) and the relevant treatment selection; the “Guidelines” section provides text chunks from the latest guidelines, which contain strict recommendations that determine the correct treatment allocation - these guidelines must be used in addition to the provided examples when selecting the most appropriate treatment; while the “Input” section contains a new clinical note from a patient with HCC, for which you need to determine the most appropriate treatment based on the provided “Instruction”, “Examples”, and “Guidelines”.

Section 1: Instruction
You are a **hepatology expert specializing in the treatment of Hepatocellular Carcinoma (HCC)**. Read the following Structured clinical report (uploaded in its original language – Italian) carefully and, based on the **patient’s characteristics and disease status**, determine the **most appropriate therapeutic option** from the list below:
A. **Liver Transplantation** (`TRAPIANTO EPATICO`)
B. **Surgical Resection** (`RESEZIONE CHIRURGICA`)
C. **Ablation** (`ABLAZIONE`) → Includes:
- **Microwave Ablation (MWA)** (*ablazione a microonde*)

- **Radiofrequency Ablation (RFA)** (*ablazione con radiofrequenza*)
- **Cryoablation** (*crioablazione*)
D. **Embolization** (`EMBOLIZZAZIONE`) → Includes:
- **Transarterial Chemoembolization (TACE)** (*chemoembolizzazione transarteriosa*)
- **Transarterial Radioembolization (TARE, also called SIRT)** (*radioembolizzazione transarteriosa, SIRT*)
- **Transarterial Embolization (TAE)** (*embolizzazione transarteriosa senza chemioterapia*)
E. **Systemic Therapy** (`TERAPIA SISTEMICA`) → Includes: **Sorafenib** - **Lenvatinib** - **Regorafenib** - **Cabozantinib** - **Atezolizumab + Bevacizumab** - **Durvalumab + Tremelimumab** - **Pembrolizumab** - **Nivolumab** -
F. **Best Supportive Care** (`BEST SUPPORTIVE CARE`) → **To be selected ONLY when all other therapeutic options (curative or palliative) have been exhausted.**: The disease is **too advanced** for locoregional or has progressed on second and third line systemic therapy. The clinical approach is limited to **symptom management and supportive care** rather than curative or life-prolonging strategies.
G. **Follow-up** (`FOLLOW-UP`) → **Only if the patient has achieved complete response (CR) and no active disease is present on the latest imaging.**

Response Format: - **If the most appropriate treatment is identified, return only the name of the treatment option** from the list above. - **Do not include any additional text in your response.**

Section 2: Examples
Example N:
Clinical Report:
"{Example Clinical Note}"
Response: {Treatment Allocation}

Section 3: Guidelines
The following text chunks are extracted from the latest guidelines related to HCC treatment and provide strict recommendations for treatment allocation. These must be used in conjunction with the examples above to determine the correct treatment.

Section 4: Input
Clinical Report:
"{Patient_Data}"
Response:

Prompt List for Case Complexity Determination

Zero-Shot Prompt [Unstructured Note]:

This prompt consists of two sections: “Instruction” and “Input”. The “Instruction” section provides details on your task, including the definitions of clinical complexity. - The “Input” section contains a new clinical note from a patient with HCC, for which you need to determine the “clinical complexity level” based on treatment allocation difficulty.

Section 1: Instruction
You are a hepatology expert specializing in the treatment of Hepatocellular Carcinoma (HCC). Read the following unstructured clinical report (uploaded in its original language – Italian) carefully and determine the clinical complexity level for treatment allocation, according to the definitions below.
Definition of Clinical Complexity in Treatment Allocation:

A. **Low Complexity (`LOW`)** → The optimal treatment allocation is **straightforward** and follows clear guideline recommendations, with no ambiguity.
B. **Moderate Complexity (`MODERATE`)** → Some elements of **uncertainty** exist, where **multiple treatment options** could be considered, but a **preferred pathway** can still be identified based on guideline interpretation.
C. **High Complexity (`HIGH`)** → The case falls into **"grey areas"** of current guidelines, where: - **Multiple therapeutic options hold comparable validity**, OR - **Patient characteristics introduce significant uncertainty** in applying standard treatment algorithms.

Important Considerations: - Complexity is assessed **only based on treatment allocation difficulty** (not overall disease severity or number of comorbidities). - If guideline-based treatment selection is **clear and direct**, classify as `LOW`. - If **some debate exists** but a clear treatment choice is still possible, classify as `MODERATE`. - If the case **does not fit standard treatment pathways** or involves **significant uncertainty**, classify as `HIGH`.

Response Format: - **Return only one of the following labels: `"LOW"`, `"MODERATE"`, or `"HIGH"`**. - **Do not include any additional text in your response.**

Section 2: Input
Clinical Report:
"{Patient_Data}"
Response:

Zero-Shot Prompt [Structured Note]:

This prompt consists of two sections: “Instruction” and “Input”. The “Instruction” section provides details on your task, including the definitions of clinical complexity. - The “Input” section contains a new clinical note from a patient with HCC, for which you need to determine the “clinical complexity level” based on treatment allocation difficulty.

Section 1: Instruction
You are a hepatology expert specializing in the treatment of Hepatocellular Carcinoma (HCC). Read the following Structured clinical report (uploaded in its original language – Italian) carefully and determine the clinical complexity level for treatment allocation, according to the definitions below.
Definition of Clinical Complexity in Treatment Allocation:

A. **Low Complexity (`LOW`)** → The optimal treatment allocation is **straightforward** and follows clear guideline recommendations, with no ambiguity.
B. **Moderate Complexity (`MODERATE`)** → Some elements of **uncertainty** exist, where **multiple treatment options** could be considered, but a **preferred pathway** can still be identified based on guideline interpretation.
C. **High Complexity (`HIGH`)** → The case falls into **"grey areas"** of current guidelines, where: - **Multiple therapeutic options hold comparable validity**, OR - **Patient characteristics introduce significant uncertainty** in applying standard treatment algorithms.

Important Considerations: - Complexity is assessed **only based on treatment allocation difficulty** (not overall disease severity or number of comorbidities). - If guideline-based treatment selection is **clear and direct**, classify as `LOW`. - If **some debate exists** but a clear treatment choice is still possible, classify as `MODERATE`. - If the case **does not fit standard treatment pathways** or involves **significant uncertainty**, classify as `HIGH`.

Response Format: - **Return only one of the following labels: `"LOW"`, `"MODERATE"`, or `"HIGH"`**. - **Do not include any additional text in your response.**

Section 2: Input
Clinical Report:
"{Patient_Data}"
Response:

Few-Shot Prompt [Unstructured Note]:

This prompt consists of three sections: “Instruction”, “Examples”, and “Input”. The Instruction section provides details on your task, including the definitions of clinical complexity. The “Examples” section includes clinical notes with corresponding complexity classifications to illustrate the classification process. The “Input” section contains a new clinical note from a patient with HCC, for which you need to determine the **clinical complexity level** based on treatment allocation difficulty.

Section 1: Instruction
You are a hepatology expert specializing in the treatment of Hepatocellular Carcinoma (HCC). Read the following unstructured clinical report (uploaded in its original language – Italian) carefully and determine the clinical complexity level for treatment allocation, according to the definitions below.
Definition of Clinical Complexity in Treatment Allocation:

A. **Low Complexity (`LOW`)** → The optimal treatment allocation is **straightforward** and follows clear guideline recommendations, with no ambiguity.
B. **Moderate Complexity (`MODERATE`)** → Some elements of **uncertainty** exist, where **multiple treatment options** could be considered, but a **preferred pathway** can still be identified based on guideline interpretation.
C. **High Complexity (`HIGH`)** → The case falls into **"grey areas"** of current guidelines, where: - **Multiple therapeutic options hold comparable validity**, OR - **Patient characteristics introduce significant uncertainty** in applying standard treatment algorithms.

Important Considerations: - Complexity is assessed **only based on treatment allocation difficulty** (not overall disease severity or number of comorbidities). - If guideline-based treatment selection is **clear and direct**, classify as `LOW`. - If **some debate exists** but a clear treatment choice is still possible, classify as `MODERATE`. - If the case **does not fit standard treatment pathways** or involves **significant uncertainty**, classify as `HIGH`.

Response Format: - **Return only one of the following labels: `"LOW"`, `"MODERATE"`, or `"HIGH"`**. - **Do not include any additional text in your response.**

Section 2: Examples
Example N:
Clinical Report:
"{Example Clinical Note}"
Response: {Clinical Complexity Level}

Section 3: Input
Clinical Report:
"{Patient_Data}"
Response:

Few-Shot Prompt [Structured Note]:
This prompt consists of three sections: “Instruction”, “Examples”, and “Input”. The Instruction section provides details on your task, including the definitions of clinical complexity. The “Examples” section includes clinical notes with corresponding complexity classifications to illustrate the classification process. The “Input” section contains a new clinical note from a patient with HCC, for which you need to determine the **clinical complexity level** based on treatment allocation difficulty.

Section 1: Instruction
You are a hepatology expert specializing in the treatment of Hepatocellular Carcinoma (HCC). Read the following Structured clinical report (uploaded in its original language – Italian) carefully and determine the clinical complexity level for treatment allocation, according to the definitions below.
Definition of Clinical Complexity in Treatment Allocation:

A. **Low Complexity (`LOW`)** → The optimal treatment allocation is **straightforward** and follows clear guideline recommendations, with no ambiguity.
B. **Moderate Complexity (`MODERATE`)** → Some elements of **uncertainty** exist, where **multiple treatment options** could be considered, but a **preferred pathway** can still be identified based on guideline interpretation.
C. **High Complexity (`HIGH`)** → The case falls into **"grey areas"** of current guidelines, where: - **Multiple therapeutic options hold comparable validity**, OR - **Patient characteristics introduce significant uncertainty** in applying standard treatment algorithms.

Important Considerations: - Complexity is assessed **only based on treatment allocation difficulty** (not overall disease severity or number of comorbidities). - If guideline-based treatment selection is **clear and direct**, classify as `LOW`. - If **some debate exists** but a clear treatment choice is still possible, classify as `MODERATE`. - If the case **does not fit standard treatment pathways** or involves **significant uncertainty**, classify as `HIGH`.

Response Format: - **Return only one of the following labels: `"LOW"`, `"MODERATE"`, or `"HIGH"`**. - **Do not include any additional text in your response.**

Section 2: Examples
Example N:
Clinical Report:
"{Example Clinical Note}"
Response: {Clinical Complexity Level}

Section 3: Input
Clinical Report:
"{Patient_Data}"
Response:

Few-Shot+RAG Prompt [Unstructured Note]:

This prompt consists of four sections: “Instruction”, “Examples”, “Guidelines”, and “Input”. - The “Instruction” section provides details on your task, including the definitions of clinical complexity. The “Examples” section includes clinical notes with corresponding complexity classifications to illustrate the classification process. The “Guidelines” section provides text chunks from the latest guidelines, which contain strict recommendations that influence treatment allocation complexity. These guidelines must be used in addition to the provided examples when determining the complexity level. The “Input” section contains a new clinical note from a patient with HCC, for which you need to determine the **clinical complexity level** based on treatment allocation difficulty.

Section 1: Instruction
You are a hepatology expert specializing in the treatment of Hepatocellular Carcinoma (HCC). Read the following unstructured clinical report (uploaded in its original language – Italian) carefully and determine the clinical complexity level for treatment allocation, according to the definitions below.
Definition of Clinical Complexity in Treatment Allocation:

A. **Low Complexity (`LOW`)** → The optimal treatment allocation is **straightforward** and follows clear guideline recommendations, with no ambiguity.
B. **Moderate Complexity (`MODERATE`)** → Some elements of **uncertainty** exist, where **multiple treatment options** could be considered, but a **preferred pathway** can still be identified based on guideline interpretation.
C. **High Complexity (`HIGH`)** → The case falls into **"grey areas"** of current guidelines, where: - **Multiple therapeutic options hold comparable validity**, OR - **Patient characteristics introduce significant uncertainty** in applying standard treatment algorithms.

Important Considerations: - Complexity is assessed **only based on treatment allocation difficulty** (not overall disease severity or number of comorbidities). - If guideline-based treatment selection is **clear and direct**, classify as `LOW`. - If **some debate exists** but a clear treatment choice is still possible, classify as `MODERATE`. - If the case **does not fit standard treatment pathways** or involves **significant uncertainty**, classify as `HIGH`.

Response Format: - **Return only one of the following labels: `"LOW"`, `"MODERATE"`, or `"HIGH"`**. - **Do not include any additional text in your response.**

Section 2: Examples
Example N:
Clinical Report:
"{Example Clinical Note}"
Response: {Clinical Complexity Level}

Section 3: Guidelines

The following text chunks are extracted from the latest guidelines related to HCC treatment and provide strict recommendations for treatment allocation. These must be used in conjunction with the examples above to determine the correct case complexity.

Section 4: Input
Clinical Report:
"{Patient_Data}"
Response:

Few-Shot+RAG Prompt [Structured Note]:

This prompt consists of four sections: “Instruction”, “Examples”, “Guidelines”, and “Input”. - The “Instruction” section provides details on your task, including the definitions of clinical complexity. The “Examples” section includes clinical notes with corresponding complexity classifications to illustrate the classification process. The “Guidelines” section provides text chunks from the latest guidelines, which contain strict recommendations that influence treatment allocation complexity. These guidelines must be used in addition to the provided examples when determining the complexity level. The “Input” section contains a new clinical note from a patient with HCC, for which you need to determine the **clinical complexity level** based on treatment allocation difficulty.

Section 1: Instruction
You are a hepatology expert specializing in the treatment of Hepatocellular Carcinoma (HCC). Read the following Structured clinical report (uploaded in its original language – Italian) carefully and determine the clinical complexity level for treatment allocation, according to the definitions below.
Definition of Clinical Complexity in Treatment Allocation:

A. **Low Complexity (`LOW`)** → The optimal treatment allocation is **straightforward** and follows clear guideline recommendations, with no ambiguity.
B. **Moderate Complexity (`MODERATE`)** → Some elements of **uncertainty** exist, where **multiple treatment options** could be considered, but a **preferred pathway** can still be identified based on guideline interpretation.
C. **High Complexity (`HIGH`)** → The case falls into **"grey areas"** of current guidelines, where: - **Multiple therapeutic options hold comparable validity**, OR - **Patient characteristics introduce significant uncertainty** in applying standard treatment algorithms.

Important Considerations: - Complexity is assessed **only based on treatment allocation difficulty** (not overall disease severity or number of comorbidities). - If guideline-based treatment selection is **clear and direct**, classify as `LOW`. - If **some debate exists** but a clear treatment choice is still possible, classify as `MODERATE`. - If the case **does not fit standard treatment pathways** or involves **significant uncertainty**, classify as `HIGH`.

Response Format: - **Return only one of the following labels: `"LOW"`, `"MODERATE"`, or `"HIGH"`**. - **Do not include any additional text in your response.**

Section 2: Examples
Example N:
Clinical Report:
"{Example Clinical Note}"
Response: {Clinical Complexity Level}

Section 3: Guidelines
The following text chunks are extracted from the latest guidelines related to HCC treatment and provide strict recommendations for treatment allocation. These must be used in conjunction with the examples above to determine the correct case complexity.

Section 4: Input
Clinical Report:
"{Patient_Data}"
Response:

Fig. S1: Confusion matrix for treatment allocation by GPT-oss-120B with few-shot without RAG and with few-shot+RAG on structured notes

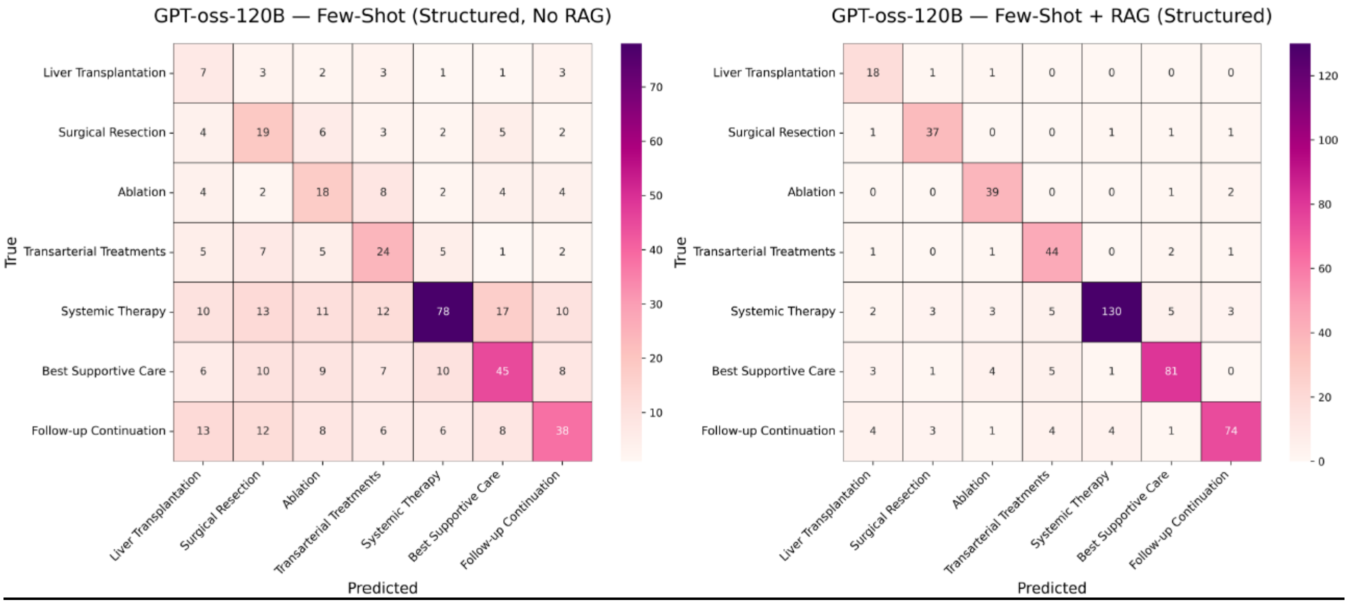

Fig. S2: Confusion matrix for clinical complexity by GPT-oss-120B with few-shot without RAG and with few-shot+RAG on structured notes

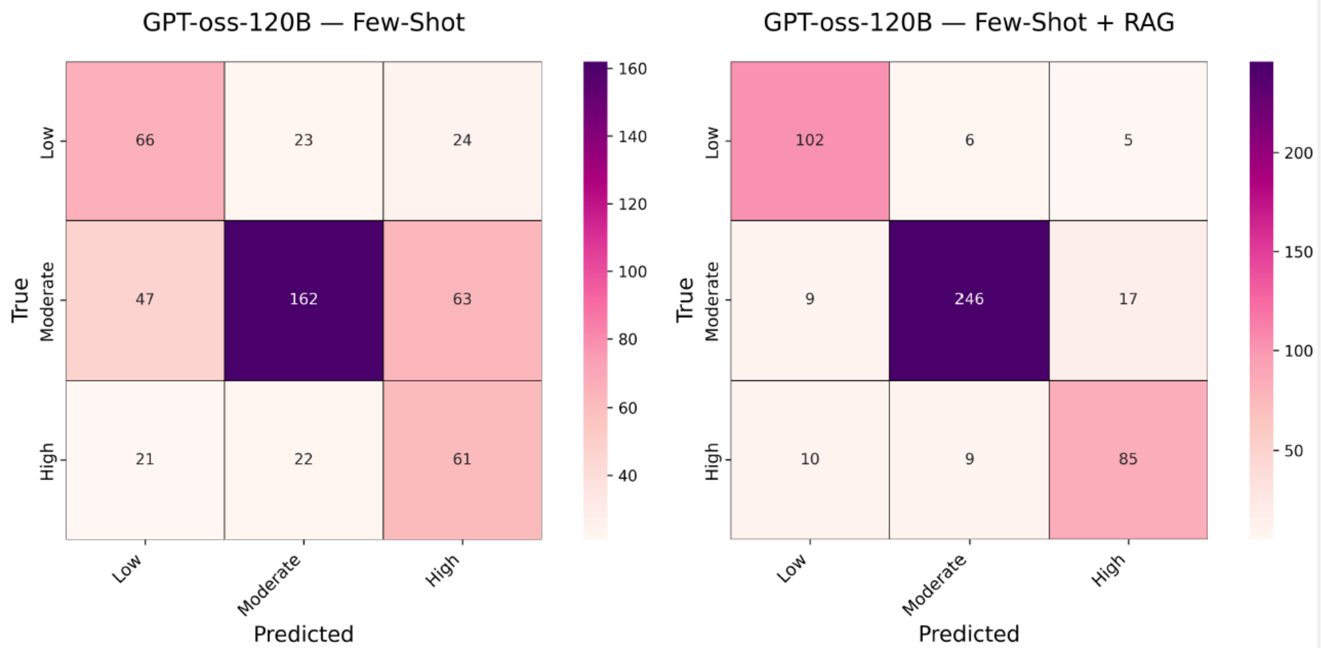

Supplement: Multimedia component 1 [file mmc1.pdf]
