# Supplementary material for: PHENO-RAG: An artificial intelligence tool for guideline-informed management decisions in hepatocellular carcinoma
Source: JHEP Rep. 2025 Dec 19;8(4):101715. doi: 10.1016/j.jhepr.2025.101715 (PMC12995883; doi:10.1016/j.jhepr.2025.101715)

JHEP|Reports

PHENO-RAG: An artificial intelligence tool for guideline-informed management decisions in hepatocellular carcinoma

Authors

Ciro Celsa, Mauro Giuffrè, Gabriele Di Maria, …, Giuseppe Cabibbo, Giansalvo Cirrincione, Calogero Cammà

Correspondence

Calogero.camma@unipa.it (C. Cammà).

Graphical abstract

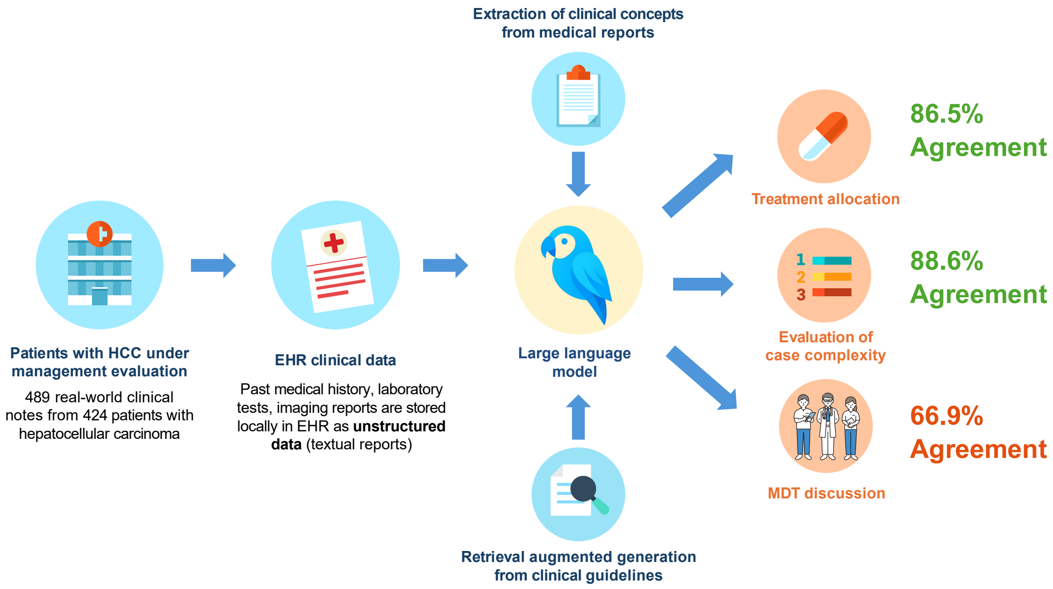

Highlights:

- PHENO-RAG is an AI system that extracts clinical data from real-world notes to provide guideline-based HCC treatment guidance.
- Automated structuring of clinical notes significantly improved decision accuracy.
- PHENO-RAG achieved 86.5% concordance with physicians for treatment allocation.
- Clinical complexity assessment showed 88.6% agreement with expert evaluation.
- PHENO-RAG is useful to support treatment decisions and complexity assessment in HCC.

Impact and implications:

Clinical decisions in the management of hepatocellular carcinoma are complex and multiparametric, requiring resource-intensive multidisciplinary care and creating challenges for optimal treatment allocation across different healthcare settings. We developed PHENO-RAG, a large language model-based framework that combines patient phenotyping through automated clinical information extraction from real-world clinical notes with treatment decision support, based on international guidelines. Our framework demonstrated concordance of 86.5% with real-world clinical decisions for treatment allocation and 88.6% for clinical complexity assessment, suggesting potential to enhance decision consistency and quality of care. In clinical practice, this AI-assisted framework could help standardize hepatocellular carcinoma management workflows, support training of hepatology and oncology fellows, assist in quality assurance programs, and facilitate more systematic identification of complex cases requiring multidisciplinary consultation, particularly in resource-constrained settings.

https://doi.org/10.1016/j.jhepr.2025.101715

© 2025 The Authors. Published by Elsevier B.V. on behalf of European Association for the Study of the Liver (EASL). This is an open access article under the CC BY license (http://creativecommons.org/licenses/by/4.0/). JHEP Reports, 2026, 8, 1–13

PHENO-RAG: An artificial intelligence tool for guideline-informed management decisions in hepatocellular carcinoma

Ciro Celsa[1,2,†], **Mauro Giuffrè**[3,4,†], Gabriele Di Maria[5], Salvatore Gruttadauria[6,7], Ugo Palazzo[8], Roberto Miraglia[9], Luigi Maruzzelli[9], Duilio Pagano[6], Roberto Cannella[10], Federico Midiri[9], Roberta Ciccia[1], Mauro Salvato[1], Alessandro Grova[1], Sofia Rao[1], Gaetano Giusino[1], Alessio Quartararo[1], Guido Cusimano[1], Alba Sparacino[1], Valeria Gaudioso[1], Valeria Genovese[1], Rosangela Montenegro[1], Claudia La Mantia[1], Francesco Mercurio[1], Simone Kresevic[11], Milos Ajcevic[11], Giuseppe Cabibbo[1], Giansalvo Cirrincione[12], Calogero Cammà[1,*]

JHEP Reports **2026**. vol. 8 | 1–13

Check for updates

Background & Aims: Management of hepatocellular carcinoma (HCC) poses unique challenges due to its development in the context of chronic liver disease and the availability of multiple treatment options. Although multidisciplinary team (MDT) management improves outcomes, universal MDT discussion is resource-intensive, underscoring the need for effective patient-stratification tools. We developed a novel large language model (LLM) framework, PHENO-RAG, that integrates contemporary HCC management guidelines with patient-specific clinical data.

Methods: We retrospectively analysed 489 clinical reports from 424 patients treated at a tertiary referral centre between September 2020 and November 2024. Eight locally hosted LLMs were tested: Llama-3-8B/70B, GPT-oss-20B/120B, Qwen-3-8B/80B, and Falcon-7B/40B. Two ablation studies assessed clinical concept extraction (using REGEX, pure LLMs, and hybrid REGEX+LLM pipelines) and decision generation across six configurations (zero-shot/few-shot with unstructured *vs*. structured notes, with and without retrieval-augmented generation [RAG] using clinical guidelines). The primary outcome was exact-match accuracy against real-world clinical decisions for treatment allocation, clinical complexity, and recommendation for MDT discussion.

Results: GPT-oss-120B+REGEX achieved the best overall agreement (median F1 for categorical concepts 0.92 [95% CI 0.85–0.95]; median intraclass correlation coefficient for numerical parameters 0.93 [95% CI 0.85–0.94]). For decision support, accuracy increased with structured inputs, few-shot exemplars, and RAG across all models. Under the strongest configuration (few-shot+RAG on structured notes), GPT-oss-120B reached 86.5% exact match for treatment allocation, 88.6% for clinical complexity, and 66.9% for MDT recommendation; Llama-3-70B achieved 80.8%, 83.4%, and 63.0%, respectively. Performance in the baseline zero-shot, unstructured-note configuration was substantially lower.

Conclusions: PHENO-RAG delivers accurate, guideline-concordant support for HCC treatment allocation and complexity grading from real-world notes, with performance driven less by model family alone than by hybrid extraction, input structuring, in-context examples, and evidence retrieval. MDT referral remains the hardest task – appropriate for prioritization rather than automation. Prospective, multi-site and multimodal validation is warranted.

© 2025 The Authors. Published by Elsevier B.V. on behalf of European Association for the Study of the Liver (EASL). This is an open access article under the CC BY license (http://creativecommons.org/licenses/by/4.0/).

Introduction

Hepatocellular carcinoma (HCC) represents the sixth most prevalent cancer and the third leading cause of cancer mortality, imposing a major health burden worldwide.[1] Unlike other malignancies, challenges associated with the management of HCC are related to several factors: 1) it develops primarily in the context of chronic liver disease, mainly cirrhosis; 2) diagnosis is usually radiological, rather than histopathological; 3) the co-existence with underlying cirrhosis influences prognosis and treatment outcomes, due to competing risks between oncological outcomes and liver-related events;[2] 4) the availability of multiple treatment options – including liver transplantation, surgical resection, ablative therapies, trans-arterial treatments, and systemic therapies, representing a challenge in risk stratification and identification of best treatment sequence. All in all, this leads to a complex decision-making landscape for clinicians.[3]

Evidence from retrospective studies consistently demonstrates that multidisciplinary team (MDT) management is significantly associated with patient outcomes in HCC.[4,5] The establishment and implementation of MDT care was shown to be significantly associated with improved survival across different healthcare settings.[6–9] The reasons for the survival benefit associated with MDT management are not fully

* Corresponding author. Address: Section of Gastroenterology and Hepatology, Department of Health Promotion, Mother and Child Care, Internal Medicine and Medical Specialties PROMISE, University of Palermo, Piazza delle Cliniche n 2, 90127, Palermo, Italy.
E-mail address: Calogero.camma@unipa.it (C. Cammà).
† **Co-authorship:** Ciro Celsa and Mauro Giuffrè share co-first authorship.
https://doi.org/10.1016/j.jhepr.2025.101715

understood and may include improved personalization of care through multiparametric decision-making, enhanced access to clinical trials, and shorter time from diagnosis to treatment initiation.

However, the implementation of universal MDT discussion for all patients with HCC presents significant practical challenges. There is a lack of standardization in the definition of the core MDT and its procedures. Moreover, the process is resource-intensive and time-consuming, and not all cases may require the same level of multidisciplinary expertise. Some treatment decisions might be relatively straightforward and can be made by single specialists, while others demand complex multi-modal approaches. This underscores the need for innovative tools that can effectively stratify patients based on their clinical complexity, identifying those who would derive the greatest benefit from a comprehensive MDT approach.

Recent advances in artificial intelligence, particularly large language models (LLMs), have shown promising potential in clinical decision support and guideline interpretation.[10,11] These transformer-based neural networks, trained on large datasets from unstructured and structured sources, can process and analyse complex clinical information while accounting for multiple variables simultaneously. Recent studies have demonstrated that LLMs, when enhanced with domain-specific knowledge through techniques such as retrieval augmented generation (RAG) or supervised fine-tuning, can achieve high accuracy rates in guideline-based decision making.[12,13] Conversely, foundational LLMs have demonstrated unacceptably low accuracy in gastroenterology and hepatology clinical tasks, potentially leading to suboptimal decision making, when used by patients or non-specialists.[14] Therefore, these implementations require careful framework design, including structured guideline reformatting and advanced prompt engineering to ensure reliable outputs.

Despite these advances, there exists a significant knowledge gap in the application of LLMs to HCC decision-making. While studies have explored LLM applications in different medical specialties, none have specifically addressed their potential role in stratifying patients with HCC by clinical complexity or predicting optimal treatment decisions using real-world clinical data. The unstructured nature of real-life clinical reports presents distinct challenges compared to artificially constructed clinical cases. Navigating unstructured clinical real-world data requires LLMs to first extract relevant clinical concepts before applying decision frameworks – a two-step process with the potential for compounding errors. Therefore, we sought to leverage LLM capability for clinical concept extraction from unstructured notes to create structured clinical cases containing information relevant for HCC management.

To address this gap, we developed PHENO-RAG, a modular ensemble LLM framework that combines patient phenotyping through automated clinical information extraction with treatment decision support guided by RAG, based on EASL (European Association for the Study of the Liver) guidelines[15] and the multiparametric therapeutic hierarchy concept.[3] By integrating unstructured real-world clinical notes with current HCC management guidelines, PHENO-RAG performs sequential reasoning across two core tasks: the structured extraction of clinically relevant parameters to enable patient stratification, and the generation of guideline-informed recommendations for treatment allocation and multidisciplinary team (MDT) discussion. To minimize error propagation through these two sequential tasks, the extraction step was systematically optimized with validation against human-labelled data, and each component was independently evaluated to ensure that structured outputs meet high accuracy thresholds before being used for downstream decision-making. The framework was evaluated for its ability to predict therapeutic decisions, assess clinical complexity, and identify patients likely to benefit from MDT evaluation.

Patients and methods

Study population and data collection

We conducted a retrospective analysis of clinical reports from patients consecutively evaluated at University Hospital of Palermo (Italy), one of the hub centres of the Sicily Network for Liver Cancer,[16] between September 1, 2020, and November 1, 2024. Clinical data were systematically collected through a dedicated web-based platform (https://www.sintesihepatology.org/study/PSN), which serves as the central database for the network. The platform facilitates structured data entry including: demographic information, clinical parameters related to chronic liver disease and HCC, laboratory values, radiological findings, treatment history and response assessment, multidisciplinary board discussion details and outcomes. All clinical reports were anonymized prior to analysis. A key feature of the platform is its capability to automatically generate standardized clinical reports in Italian language, available in both Word and PDF formats, which clinicians can subsequently modify as needed.

Clinical reports selection

For patients with multiple clinical reports during the study period, we selected reports corresponding to specific therapeutic decision points. For patients maintaining complete response throughout the recruitment period and requiring only clinical-radiological follow-up, we included only the latest available report. For all other patients, therapeutic decisions were categorized as: liver transplantation, surgical resection, ablation, transarterial treatment, systemic therapy, best supportive care (BSC).

Clinical decision criteria

Clinical decisions were based on multiple parameters[3] including: age, Eastern Cooperative Oncology Group - performance status, Child-Pugh class, model for end-stage liver disease (MELD) score, presence of esophageal varices as an indicator of clinically significant portal hypertension, presence of severe extrahepatic comorbidities (adapted from comorbidities included in Charlson comorbidity index. Further details are reported in the supplementary materials), previous HCC treatments, response to previous HCC treatments, alpha-fetoprotein (AFP) levels, maximum diameter of the main nodule, number of nodules, vascular invasion and presence of extrahepatic disease.

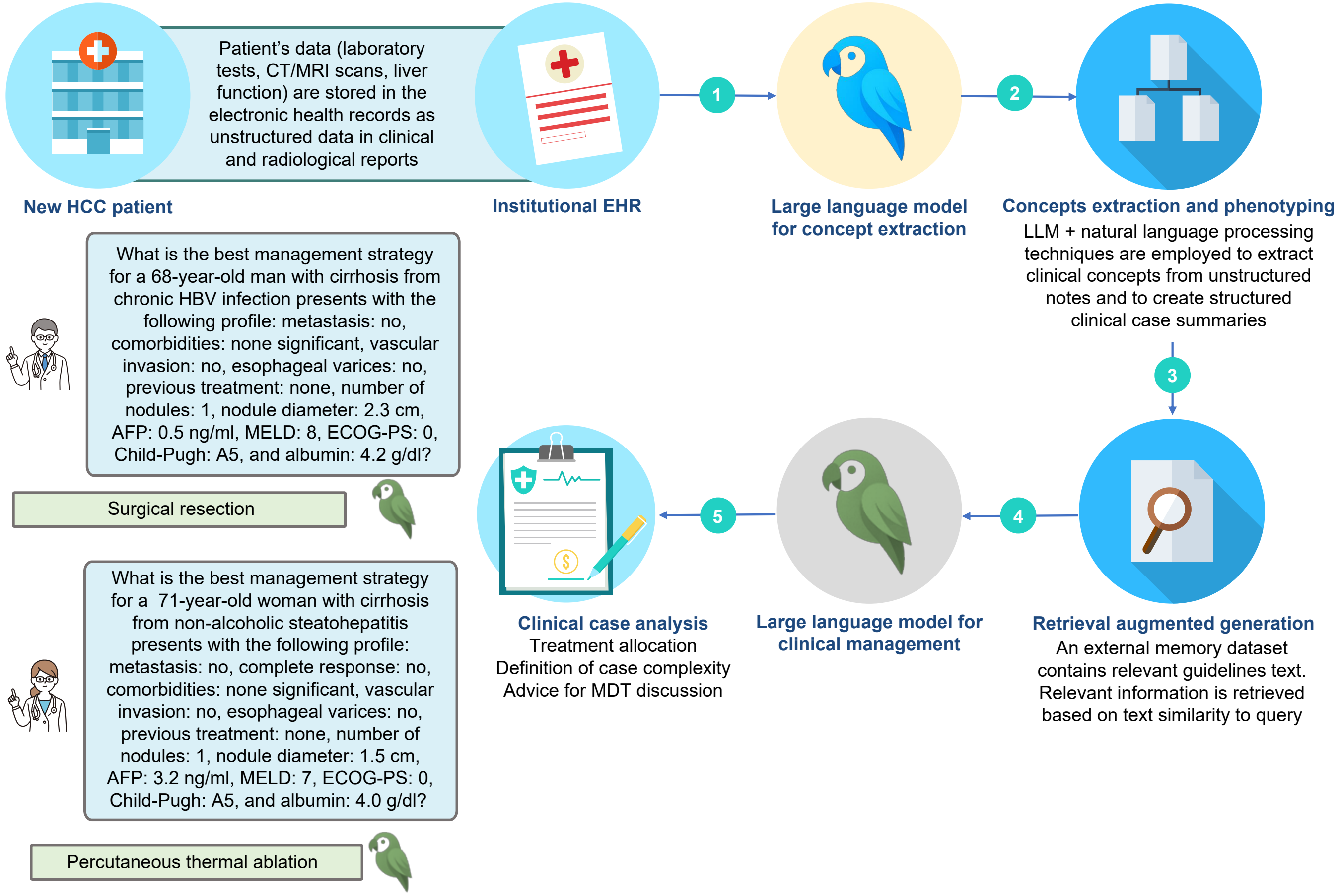

Fig. 1. PHENO-RAG framework for HCC clinical decision support system. The clinical evaluation begins with patient evaluation, with clinicians collecting comprehensive data in multiple data formats, including demographic information, liver function parameters, tumor characteristics, and imaging findings. These unstructured clinical reports are processed through a two-stage extraction system combining natural language processing techniques (*i.e.* regular expression) and multiple large language models to transform them into structured data with high fidelity. The structured clinical data is then processed by each large language model configuration using advanced prompt engineering and retrieval augmented generation, which incorporates HCC management guidelines to generate three key outputs: recommended treatment allocation, clinical complexity assessment and decision for multidisciplinary discussion. AFP, alpha-fetoprotein; ECOG-PS, Eastern Cooperative Oncology Group – performance status, HCC, hepatocellular carcinoma; MDT, multidisciplinary team; LLM, large language model; MELD, model for end-stage liver disease.

Clinical complexity assessment and multidisciplinary discussion

Clinical complexity was retrospectively assessed by two clinicians (Ci.C, and G.C.) who reviewed all clinical reports and graded complexity as low, moderate or high. In cases of disagreement between the two physicians, a third senior physician (Ca.C.) acted as an adjudicator to resolve discrepancies. For each patient, we documented whether the case was discussed by the multidisciplinary board. The board comprises hepatologists, diagnostic and interventional radiologists, resection and transplant surgeons, and oncologists. The center's standard criteria for determining which patients require multidisciplinary discussion are detailed in the supplementary materials.

PHENO-RAG framework

As illustrated in Fig. 1, the PHENO-RAG framework was designed to replicate the cognitive workflow of clinicians navigating complex HCC cases, by sequentially integrating clinical concept extraction, patient phenotyping, and evidence-based reasoning. Starting from unstructured data embedded in clinical and radiological reports, PHENO-RAG first employs LLMs and natural language processing techniques to extract key clinical variables and construct structured case profiles. Then management decisions are supported by relevant clinical guidelines with an integrated RAG system. This modular architecture enables the framework to support three essential decision-making tasks: assigning treatment recommendations, assessing clinical complexity, and identifying patients most likely to benefit from MDT discussion.

Patient phenotyping: Extraction of clinical concepts from medical reports

To identify the most effective method for clinical concept extraction, we conducted an ablation study comparing five different approaches: regular expression (REGEX)-based extraction alone, LLM-based extraction using LLMs

separately, and combined REGEX-LLM pipelines for both model variants.

To validate our clinical concept extraction approach, we randomly selected 100 full medical reports from our database for manual labelling. Two experienced physicians independently reviewed these clinical notes to extract numerical values and determine the presence/absence of key clinical features (*i.e.*, vascular invasion, severe comorbidities, metastatic disease, previous HCC treatment, complete response to last treatment, esophageal varices) or numerical values (*i.e.*, AFP levels, serum albumin, performance status, Child-Pugh score, MELD score, and maximum nodule diameter). In cases of disagreement between the two physicians, a third senior physician acted as an adjudicator to resolve discrepancies. These manually labelled clinical notes served as the gold standard for evaluating our automated extraction system's performance.

REGEX is a standardized pattern-matching language used to identify and extract specific text patterns within documents. REGEX operates by defining search patterns that can match both exact text and more complex patterns like numbers, dates, or specific word combinations. For example, a simple pattern might look for the word "nodule" followed by a number and the unit "mm", while more complex patterns can account for variations in terminology, spacing, and surrounding context. To systematically extract relevant clinical information from the reports, we developed a comprehensive REGEX-based approach. For instance, to identify vascular invasion, the system would look for patterns like "portal[\s]*-vein[\s]*thrombosis", where [\s] represents optional spaces between words. Regular expressions were employed to identify and extract both categorical features (presence/absence) and numerical parameters from the clinical narratives. For categorical features (vascular invasion, severe comorbidities, metastatic disease, previous HCC treatment, complete response to last treatment, and esophageal varices), we created an extensive dictionary of relevant terms and their variations (detailed in the supplementary materials). The extraction algorithm incorporated common negation phrases (such as "no", "absent", "without") and evaluated their presence within a three-word window before and after the target keywords to accurately determine the presence or absence of each condition. For numerical parameters (AFP, serum albumin, performance status, Child-Pugh score, MELD score), the algorithm searched for numerical values immediately preceding or following predefined keyword markers. Special consideration was given to the extraction of nodule measurements, where the algorithm first identified the most recent imaging report based on the documented date (year), then searched for specific dimensional keywords within that imaging section to determine the maximum nodule diameter.

For the LLM-based extraction approach, we implemented a system using the following locally hosted, privacy-preserving language models: Meta's Llama-3-8B and Llama-3-70B, OpenAI's GPT-oss 20B and GPT-oss-120B, Qwen's Qwen3-8B and Qwen3-Next-80B-A3B-Instruct, TII's Falcon-7B and Falcon-40B. These specific models were selected based on their demonstrated strong performance in processing Italian language text as reported in their technical documentation, which was crucial as all clinical reports in our dataset were written in Italian.[17–24] All prompts were formulated in Italian, and model outputs were generated and evaluated in the same language to ensure linguistic alignment and accuracy. For extraction, the models were configured with deterministic settings (temperature = 0, max tokens = 50) to ensure consistent outputs and minimize hallucination risks. Our prompt design (reported in the supplementary materials) was developed through an iterative optimization process, where multiple prompt variations were tested and evaluated for extraction accuracy. After several iterations and performance comparisons, we selected the best-performing prompt structure, which incorporated both chain-of-thought reasoning and few-shot learning principles. The final prompt structure began with a clear specification of the extraction task, listing all clinical parameters to be identified and their expected formats. This was followed by ten carefully selected clinical report-extraction pairs, representing diverse cases including reports with multiple findings, cases with negated conditions, various formats of numerical values, and complex scenarios requiring inference from context. Each example pair included both the input clinical report and its corresponding structured extraction output. The prompt also included explicit instructions for the model to identify relevant sections in the clinical report, extract specific values or conditions, validate extracted information against given criteria, and format the output in a consistent structure.

For the combined REGEX-LLM approach, we implemented a sequential processing pipeline where each clinical report was first analyzed by the LLM system, which attempted to extract all relevant parameters. For each parameter, the LLM would output either a specific value (numerical or categorical) or indicate "not reported" if the information could not be confidently extracted. In cases where the LLM indicated "not reported", the REGEX system was automatically triggered as a backup extraction method, providing an additional opportunity to identify and extract the missing information. This complementary approach leveraged the strengths of both methods, with the LLM's ability to understand context and complex relationships serving as the primary extraction tool, while the REGEX system acted as a safety net for cases where the LLM could not make a definitive determination.

We evaluated the performance of each extraction method against the human-labelled dataset using eight different LLMs as mentioned above. Each model was tested individually and in combination with REGEX. For clinical concepts – defined as categorical variables including vascular invasion, severe comorbidities, metastatic disease, previous HCC treatment, complete response to last treatment, and esophageal varices – we assessed the F1-score, reporting the median F1-score across all categorical variables. For numerical parameters – comprising AFP, serum albumin, performance status, Child-Pugh score, MELD score, and nodule maximum diameter – we evaluated the agreement using the intraclass correlation coefficient (ICC, two-way mixed effects), reporting the median ICC across all numerical variables. For numerical parameters, agreement with the human-labelled reference was defined as an exact match up to the first decimal place. Values differing beyond one decimal precision were classified as incorrect to ensure strict comparability with the reference annotations. For categorical variables, LLM outputs were evaluated using exact matching against human labels. Only binary responses (present/absent) matching the reference standard were considered

correct, while 'not reported' or ambiguous outputs were classified as incorrect.

LLM-enhanced clinical decision support system

To evaluate the impact of transforming unstructured clinical notes into structured formats and to systematically assess the effect of different prompt design strategies and the integration of external domain knowledge, we implemented a second ablation study focused on clinical decision generation. This study aimed to isolate the contribution of input structure (unstructured *vs.* structured clinical notes), prompting approach (zero-shot *vs*. few-shot), and the addition of evidence retrieval through RAG. We tested eight different LLMs as mentioned above – across six distinct experimental configurations: zero-shot (unstructured note), zero-shot (structured note), few-shot (unstructured note), few-shot (structured note), few-shot+RAG (unstructured note), and few-shot+RAG (structured note) as depicted in Fig. 2. For each configuration, we evaluated model performance across three core clinical tasks: treatment allocation, clinical complexity assessment, and identification of cases requiring MDT discussion. This framework allowed us to quantify the individual and combined benefits of structured input, prompt exemplars, and guideline-informed retrieval in optimizing clinical recommendation quality.

We designed a systematic evaluation framework comparing different prompting strategies: zero-shot, few-shot, and few-shot with RAG. Each strategy was tested using both raw clinical notes and structured notes containing key elements extracted through our previously described pipeline. Structured notes were generated by employing the best performing framework for note extraction (REGEX-GPT-oss-120B). This approach allowed us to assess the impact of information structure on recommendation quality.

For the few-shot experiments, we randomly selected 10 clinical cases from our database to serve as examples. These cases were presented in note-outcome pairs, maintaining consistency with the input format (structured or unstructured) used in each experimental condition, in order to provide comprehensive context for the models compatible with the model context window.

For the RAG-enhanced approach, we incorporated two key reference documents: the EASL clinical practice guidelines published in 2018[15] and a previously published paper proposing an evidence-based framework for HCC treatment based on the concept of multiparametric therapeutic hierarchy.[3] The whole document preprocessing followed our established protocols already reported in detail elsewhere[12] and involved chunking the text at the paragraph level to create meaningful, self-contained units of information.[25,26] We generated vector representations of these chunks using the all-MiniLM-L6-v2 embedding model, chosen for its robust performance in capturing medical domain semantics. During inference, our RAG system dynamically retrieved the five most relevant chunks from each reference document based on highest cosine similarity scores to the input clinical case. These retrieved chunks were then incorporated into the prompt structure to provide evidence-based context for the model's recommendations.

For each experimental condition (zero-shot, few-shot, and RAG), we developed specific prompt templates optimized for the task requirements. The zero-shot prompts contained clear task descriptions and expected output formats. Few-shot prompts included the ten example cases with their corresponding decisions, while RAG-enhanced prompts incorporated both examples and relevant retrieved passages from the reference documents.

The prompt structure for each condition was designed to maintain consistency while accommodating the specific requirements of each approach: zero-shot (direct instruction with clear specification of the three decision tasks); (2) few-shot (incorporation of the 10 example cases followed by the target case); RAG (integration of retrieved reference chunks alongside few-shot examples). This methodological framework enabled systematic evaluation of the models' capabilities

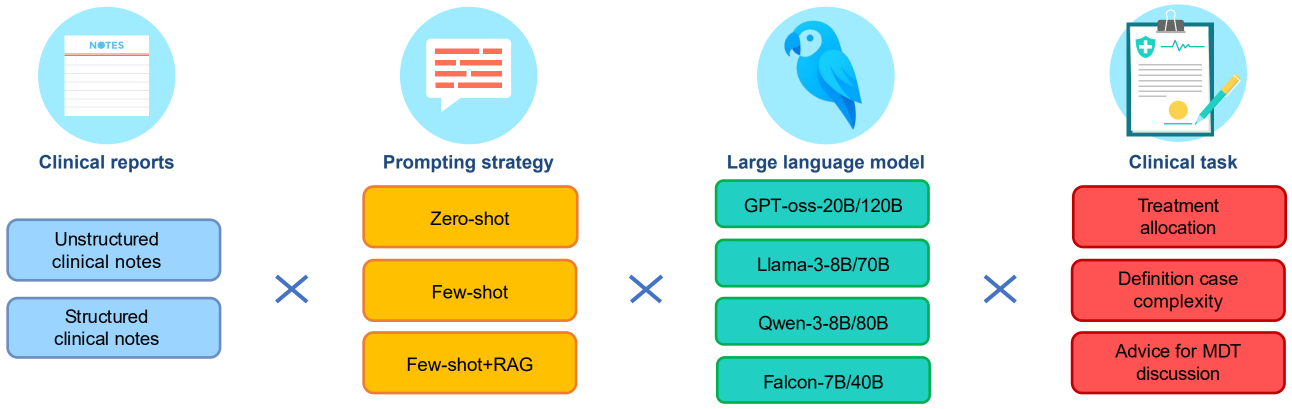

Fig. 2. Ablation study to test LLM-generated recommendation in HCC management. The diagram illustrates the comprehensive workflow for generating treatment recommendations using LLMs. The process begins with clinical reports as input (both unstructured notes and structured data), which are processed through different prompting strategies (zero-shot, few-shot, and few-shot with RAG). We evaluated eight different LLMs. The RAG component enhances model responses by incorporating domain knowledge from international guidelines and multidisciplinary expert opinion. The system generates three critical clinical outputs: treatment allocation, clinical complexity assessment, and decision for multidisciplinary discussion. HCC, hepatocellular carcinoma; LLMs, large language models; RAG, retrieval augmented generation.

across different levels of context and input structure, providing insights into the optimal approach for clinical recommendation generation.

All model interactions were configured with a temperature of 0.8 and max_tokens = 500. These hyperparameters were selected based on our prior experiments in clinical decision-support tasks, which identified this configuration as providing optimal text comprehension while minimizing the risk of hallucinations.[26,27] The maximum output length was set to accommodate comprehensive recommendations while maintaining computational efficiency.

For experiments using structured notes, we utilized the output from our previous extraction pipeline, which provided standardized representations of key clinical parameters. For unstructured experiments, we used the raw clinical notes as direct input to the models, allowing them to process the natural language content in its original form.

For model evaluation in treatment allocation, clinical complexity assessment, and MDT discussion recommendation, we computed accuracy, defined as exact match rate between LLM recommendations and real-life recommendations across all three items.

Statistical analysis

Continuous variables were summarized as mean ± standard deviation, and categorical variables as frequencies and percentages. For clinical concept extraction evaluation, we used two complementary metrics: F1 score and ICC. The F1 score was calculated for categorical variables as the harmonic mean of precision (positive predictive value) and recall (sensitivity), ranging from 0 (worst performance) to 1 (perfect performance), with higher values indicating better agreement. The ICC was computed for numerical parameters using a two-way mixed-effects model for absolute agreement, with values interpreted as: <0.50 = poor, 0.50-0.75 = moderate, 0.75-0.90 = good, and >0.90 = excellent reliability. In the main text, aggregated summaries of model agreement are reported using median, first quartile (Q1), and third quartile (Q3) across all variables within each domain. Full item-level performance metrics for every variable and model configuration are provided in the supplementary materials to enable detailed examination of extraction performance.

For clinical decision support tasks (treatment allocation, clinical complexity, and MDT recommendation), performance was evaluated using accuracy, defined as the exact match rate between LLM-generated recommendations and real-world clinical decisions, expressed as a percentage.

Confidence intervals (95% CIs) for F1 scores and ICC values were calculated using bootstrap resampling with 1,000 iterations. For each iteration, we randomly sampled with replacement from the original dataset, computed the metric, and derived CIs from the 2.5th and 97.5th percentiles of the bootstrap distribution.

Computational environment

All analyses were performed in Python 3.11 using a Linux-based high-performance computing environment with GPU acceleration. LLMs were executed locally via the Hugging Face *transformers* and *accelerate* libraries, ensuring on-premise inference and data confidentiality. Model weights for Llama-3, GPT-oss, Qwen-3, and Falcon architectures were loaded from official Hugging Face repositories and run on NVIDIA A100 and H100 GPUs. Data preprocessing and evaluation were implemented with *pandas* (v2.2.3), *numpy* (v1.26.4), and *scikit-learn* (v1.5.1), while the retrieval component relied on FAISS-based vector indexes and embeddings generated with the *all-MiniLM-L6-v2* model. This environment provided sufficient computational capacity to support inference and benchmarking of models ranging from 8B to 120B parameters.

Ethical considerations

This study was conducted in accordance with the Declaration of Helsinki and approved by Ethics Committee of University Hospital of Palermo (PSN 2017-Action 6.28). All clinical data were fully anonymized prior to analysis. Data confidentiality was maintained throughout the study, with access restricted to authorized researchers only.

Results

During study period, 489 clinical reports from 424 individual patients (mean age 71.2 ± 10.6 years, 73.3% male) were included. Clinical decisions included liver transplantation in 20 cases (4.1%), surgical resection in 41 (8.4%), ablation in 42 (8.6%), transarterial treatments in 49 (10.0%), systemic therapy in 151 (30.9%), BSC in 95 (19.4%) and follow-up continuation in 91 (18.6%).

Case complexity was judged as low in 113 cases (23.1%), moderate in 272 (55.6%) and high in 104 (21.3%). The distribution of clinical decisions according to case complexity is reported in Table 1.

Multidisciplinary discussion was undertaken in 63 cases (14.9%) (mean age 68.3 ± 11.9 years, 75.0% male). Among them, clinical decisions included liver transplantation in 10 cases (15.9%), surgical resection in 18 (28.6%), ablation in 12 (19.0%), transarterial treatments in 4 (6.3%), systemic therapy in 15 (23.8%), BSC in 2 (3.2%) and follow-up continuation in 2 (3.2%).

Accuracy analysis of the clinical concept extraction from clinical reports

The composition of 100 cases used for validating the clinical concept extraction approach is reported in the supplementary materials. Accuracy metrics for both categorical clinical concepts (evaluated with the F1 score) and continuous numerical parameters (evaluated with the ICC) are summarized in Table 2. Overall, LLMs outperformed the baseline REGEX approach across both domains. The best performance was achieved by GPT-oss-120B combined with REGEX, which reached a median F1 score of 0.92 (95% CI 0.85–0.95) for clinical concepts and an ICC of 0.93 (95% CI 0.85–0.94) for numerical parameters. Llama-3-70B+REGEX also demonstrated consistently strong results (F1 0.90, 95% CI 0.79–0.91; ICC 0.90, 95% CI 0.80–0.92), while Qwen-3-80B+REGEX achieved intermediate performance (F1 0.88, 95% CI 0.80–0.93; ICC 0.82, 95% CI 0.70–0.83). In contrast, Falcon-based models showed lower accuracy overall, although their performance improved modestly with REGEX integration. Model-specific performance metrics for extraction of individual clinical concepts and numerical parameters is reported in

Tables S2 and S3, respectively. GPT-oss-120B combined with REGEX achieved the highest overall performance, with the best extraction accuracy for previous HCC treatment (F1 = 0.98) and AFP levels (ICC = 0.99), whereas its lowest scores were observed for metastatic disease (F1 = 0.80) and nodule diameter (ICC = 0.78). Per-parameter extraction performance of GPT-oss-120B+REGEX is reported in Fig. 3.

Agreement analysis of treatment allocation

For treatment allocation, GPT-oss-120B achieved the highest performance across all experimental conditions, with exact match rates ranging from 28.8% (zero-shot on unstructured notes) to 86.5% (few-shot+RAG on structured notes). Llama-3-70B demonstrated strong performance, with exact match rates increasing from 25.8% for zero-shot on unstructured notes to 80.8% for few-shot+RAG on structured notes. In contrast, Falcon-7B showed the poorest performance, with accuracy ranging from only 12.5% to 42.3% across all experimental conditions. All models demonstrated consistent improvement with structured data input, few-shot examples, and RAG integration (Table 3).

The confusion matrix showing the distribution of treatment assignments by GPT-oss-120B with few-shot+RAG on structured notes, including misclassification patterns across all treatment categories, is reported in Fig. S1. In the few-shot setting without RAG, the model correctly classified 229 patients, with several consistent misclassification patterns. The most frequent confusion involved ablation and transarterial treatments, with 23 total bidirectional errors. Ablation remained one of the lowest-performing categories, with 18/42 correct classifications (42.9%), while systemic therapy – despite being the most prevalent category – achieved 78/151 correct classifications (51.7%). BSC performed similarly, with 45/95 correct classifications (47.4%), and follow-up continuation reached 38/91 (41.8%). The addition of RAG substantially improved model performance, increasing overall correct classifications to 425 patients – a net gain of +196 compared with few-shot alone. The most substantial improvements occurred in categories with previously low accuracy. Systemic therapy improved from 78 to 130 correct predictions (+52), ablation from 18 to 39 (+21), and transarterial treatments from 24 to 44 (+20). Follow-up continuation increased from 38 to 74 (+36), and surgical resection from 19 to 37 (+18).

Fig. 4 depicts the treatment allocation accuracy across prompting strategies for GPT-oss-120B, showing that the integration of RAG consistently improved classification accuracy across all treatment categories, with the largest gains observed for systemic therapy, ablation, and transarterial therapies.

Agreement analysis of clinical complexity

Similar patterns emerged for clinical complexity assessment. GPT-oss-120B again achieved the highest accuracy rates, ranging from 37.0% (zero-shot on unstructured notes) to 88.6% (few-shot+RAG on structured notes). Llama-3-70B showed comparable performance, with exact match rates improving from 32.9% to 83.4% across the experimental conditions. Falcon-7B consistently performed worst, with accuracy rates between 17.8% and 46.8%. The progressive enhancement from basic zero-shot prompting to the full few-shot+RAG approach with structured data was evident across all models (Table 3).

The confusion matrix showing clinical complexity assessment by GPT-oss-120B with few-shot+RAG on structured notes is depicted in Fig. S2. In the few-shot configuration, GPT-oss-120B exhibited a high rate of bidirectional misclassification, with frequent overestimation and underestimation of clinical complexity. Overestimation was the dominant pattern (110 errors), driven mainly by incorrect upgrading of low-complexity cases into the moderate or high categories and misclassification of moderate cases as high complexity. Underestimation (90 errors) was also common and largely reflected the tendency to downgrade moderate cases to low complexity and high-complexity cases to moderate or low complexity. The introduction of retrieval (few-shot+RAG) substantially altered the model's error profile. Overall misclassification dropped from 200 to 56 cases, and – critically – the directionality of errors became balanced, with underestimation and overestimation occurring at identical frequencies (28 *vs.* 28 cases). Underestimation primarily involved moderate cases predicted as low complexity and high cases predicted as moderate, whereas overestimation stemmed mostly from isolated upgrades of low-complexity notes into the moderate or high category.

Agreement analysis of decisions for multidisciplinary discussion

MDT recommendation proved the most challenging task for all models. GPT-oss-120B achieved the best performance with exact match rates ranging from 18.2% (zero-shot on unstructured notes) to 66.9% (few-shot+RAG on structured notes). Llama-3-70B demonstrated similar trends, improving from 16.2% to 63.0% across experimental conditions. Falcon-7B showed the lowest accuracy, with rates between 8.0% and 33.3%. Even the best-performing models struggled with this task compared to treatment allocation and clinical complexity assessment (Table 2).

Discussion

HCC management presents unique challenges due to the complex interplay between tumor characteristics, underlying chronic liver disease, and patient-specific factors. The integration of LLMs into clinical decision support systems could represent a promising frontier in healthcare delivery, particularly for complex diseases like HCC, where management decisions are multiparametric, and encompass multiple factors.

In this retrospective study of 489 real-world HCC clinical notes, we developed PHENO-RAG, an AI-assisted system designed to extract clinical information from narrative reports and provide clinical recommendations via LLMs, supported by international guidelines. Across eight models tested with different prompting/knowledge conditions, the accuracy of clinical decisions dramatically improved when the models were provided with structured clinical information and access to current management guidelines. The best overall results were obtained with GPT-oss-120B, followed by Llama-3-70B, while other tested models showed lower overall performance, but still benefitted from the same methodological enhancements. These findings support the potential for LLM-assisted tools to

Table 1. Distribution of clinical decisions according to case complexity.

Management decision	Low complexity (n = 113)	Moderate complexity (n = 272)	High complexity (n = 104)
Liver transplantation	5 (4.4%)	11 (4.0%)	4 (3.8%)
Surgical resection	9 (8.0%)	24 (8.8%)	8 (7.7%)
Ablation	10 (8.8%)	24 (8.8%)	8 (7.7%)
Transarterial treatments	11 (9.7%)	27 (9.9%)	11 (10.6%)
Systemic therapy	35 (31.0%)	84 (30.9%)	32 (30.8%)
Best supportive care	22 (19.5%)	52 (19.1%)	21 (20.2%)
Follow-up continuation	21 (18.6%)	50 (18.4%)	20 (19.2%)

Distribution of management decisions stratified by case complexity. Values are presented as absolute counts and column percentages, reflecting the proportion of each decision category within the respective complexity level.

enhance consistency and quality of HCC management decisions in clinical practice.

The first step of our approach was to assess the ability of our framework to extract key determinants of clinical decisions from unstructured clinical notes, thus obtaining structured data for subsequent clinical decision-making. This automated structuring process represents a critical advance, as it automatically transforms raw narrative clinical reports into systematically organized information that can be more reliably processed and analyzed. A key finding of our study is represented by the significant performance disparities across different models for the accurate identification of key clinical parameters influencing clinical decisions, such as tumor characteristics, liver function parameters, and response to previous HCC treatments. Combined approaches with GPT-oss-120B plus REGEX (a standardized text-searching method for identifying and extracting specific medical terms from clinical text) achieved superior clinical concept extraction, with accuracy higher than 90%, substantially outperforming smaller models. This aligns with recent evaluations showing that combined approaches are more accurate in medical applications.[28,29] The integration of REGEX with LLM processing proved critical, aligning with previous findings observed in other hepatological settings,[12] where guideline reformatting and structured presentation of clinical information led to similar improvements in accuracy. The performance gap between GPT-oss-120B and smaller models was substantial across all conditions, highlighting the importance of model selection in clinical applications.[30] Our extraction performance is consistent with recent systematic evidence showing that natural language processing systems achieve sensitivity and specificity higher than 90% in extracting information from free-text medical reports.[31]

We then assessed the performance of our framework in generating clinical recommendations, that were compared with real-world specialist decisions across three clinical tasks: HCC treatment allocation, grading of clinical complexity and identification of need for MDT discussion. Our framework demonstrated high concordance with real-world decisions for treatment allocation and clinical complexity assessment, suggesting potential to support clinical workflows while maintaining quality of care. Interestingly, our framework demonstrated progressive improvement across all models with structured data preprocessing, access to representative clinical examples (few-shot learning), and guideline-informed recommendations through RAG integration.

Table 2. Accuracy analysis of the extraction of clinical parameters from clinical reports.

Configurations	Clinical concepts	Numerical parameters
REGEX	0.62 (0.50;0.68)	0.87 (0.74;0.89)
**Meta's models**		
Llama-3-8B	0.81 (0.68;0.83)	0.81 (0.67;0.86)
Llama-3-8B+REGEX	0.84 (0.71;0.86)	0.84 (0.71.0.85)
Llama-3-70B	0.87 (0.73;0.88)	0.89 (0.77;0.92)
Llama-3-70B+REGEX	0.90 (0.79;0.91)	0.90 (0.80;0.92)
**OpenAI's models**		
GPT-oss-20B	0.83 (0.70;0.85)	0.80 (0.73;0.84)
GPT-oss-20B+REGEX	0.88 (0.81;0.93)	0.85 (0.71;0.87)
GPT-oss-120B	0.85 (0.77;0.90)	0.90 (0.83;0.95)
GPT-oss-120B+REGEX	0.92 (0.85;0.95)	0.93 (0.85;0.94)
**Qwen's models**		
Qwen-3-8B	0.79 (0.65;0.85)	0.70 (0.65;0.76)
Qwen-3-8B+REGEX	0.85 (0.77;0.91)	0.79 (0.69;0.83)
Qwen-3-80B	0.83 (0.74;0.92)	0.76 (0.65;0.81)
Qwen-3-80B+REGEX	0.88 (0.80;0.93)	0.82 (0.70;0.83)
**TII's models**		
Falcon-7B	0.65 (0.60;0.71)	0.69 (0.65;0.73)
Falcon-7B+REGEX	0.71 (0.65;0.74)	0.72 (0.64;0.75)
Falcon-40B	0.69 (0.64;0.71	0.75 (0.70;0.79)
Falcon-40B+REGEX	0.73 (0.66;0.78)	0.82 (0.74;0.83)

Extraction performance for *clinical concepts* – defined as categorical entities including vascular invasion, severe comorbidities, metastatic disease, previous hepatocellular carcinoma treatment, complete response to the last treatment, and presence of esophageal varices – was evaluated using the median F1 score**.** In contrast, *numerical parameters* – comprising alpha-fetoprotein, albumin, performance status, Child-Pugh score, model for end-stage liver disease score, and nodule diameter – were assessed using the median ICC to capture agreement in continuous data extraction. ICC, intraclass correlation coefficient.

Table 3. Performance comparison of large language models across clinical decision-making tasks for hepatocellular carcinoma management.

Models and Tasks	Zero-shot (unstructured note)	Zero-shot (structured note)	Few-shot (unstructured note)	Few-shot (structured note)	Few-shot+RAG (unstructured note)	Few-shot+RAG (structured note)
**Llama-3-8B**						
Treatment allocation	97 (19.83%)	119 (24.33%)	142 (29.03%)	159 (32.51%)	182 (37.21%)	240 (49.07%)
Clinical case complexity	139 (28.47%)	142 (29.04%)	218 (44.58%)	241 (49.28%)	273 (55.83%)	301 (61.55%)
MDT recommendation	62 (12.67%)	67 (13.70%)	81 (16.56%)	93 (19.01%)	107 (21.88%)	115 (23.51%)
**Llama-3-70B**						
Treatment allocation	126 (25.76%)	168 (34.35%)	187 (38.24%)	201 (41.10%)	242 (49.48%)	395 (80.77%)
Clinical case complexity	161 (32.92%)	178 (36.4%)	226 (46.21%)	260 (53.17%)	292 (59.71%)	408 (83.43%)
MDT recommendation	79 (16.15%)	92 (18.81%)	101 (20.65%)	129 (23.38%)	254 (51.94%)	308 (62.98%)
**GPT-oss-20B**						
Treatment allocation	133 (27.19%)	177 (36.19%)	202 (41.30%)	221 (45.20%)	263 (53.79%)	305 (62.37%)
Clinical case complexity	172 (35.17%)	186 (38.04%)	246 (50.31%)	281 (57.47%)	322 (65.85%)	341 (69.73%)
MDT recommendation	84 (17.18%)	96 (19.63%)	112 (22.90%)	137 (28.02%)	169 (34.56%)	230 (47.03%)
**GPT-oss-120B**						
Treatment allocation	141 (28.84%)	186 (38.04%)	212 (43.35%)	229 (46.83%)	269 (55.01%)	423 (86.51%)
Clinical case complexity	181 (37.01%)	192 (39.26%)	254 (51.95%)	289 (59.10%)	329 (67.28%)	433 (88.55%)
MDT recommendation	89 (18.20%)	103 (21.07%)	119 (24.34%)	144 (29.45%)	178 (36.40%)	327 (66.87%)
**Qwen-3-8B**						
Treatment allocation	85 (17.38%)	104 (21.27%)	128 (26.17%)	147 (30.06%)	169 (34.56%)	289 (59.10%)
Clinical case complexity	121 (24.74%)	133 (27.19%)	176 (36.01%)	198 (40.49%)	226 (46.21%)	311 (63.61%)
MDT recommendation	55 (11.25%)	61 (12.47%)	73 (14.93%)	86 (17.58%)	101 (20.65%)	223 (45.60%)
**Qwen-3-80B**						
Treatment allocation	98 (20.04%)	121 (24.74%)	147 (30.06%)	163 (33.33%)	194 (39.67%)	331 (67.69%)
Clinical case complexity	137 (28.02%)	152 (31.08%)	194 (39.67%)	219 (44.78%)	243 (49.69%)	351 (71.78%)
MDT recommendation	64 (13.09%)	71 (14.52%)	88 (18.00%)	107 (21.88%)	129 (26.38%)	251 (51.33%)
**Falcon-7B**						
Treatment allocation	61 (12.47%)	73 (14.93%)	84 (17.18%)	96 (19.63%)	118 (24.13%)	207 (42.33%)
Clinical case complexity	87 (17.80%)	104 (21.27%)	121 (24.74%)	133 (27.19%)	152 (31.08%)	229 (46.83%)
MDT recommendation	39 (7.98%)	46 (9.40%)	53 (10.84%)	64 (13.09%)	73 (14.93%)	163 (33.33%)
**Falcon-40B**						
Treatment allocation	71 (14.52%)	89 (18.20%)	101 (20.65%)	118 (24.13%)	144 (29.45%)	246 (50.31%)
Clinical case complexity	102 (20.86%)	117 (23.92%)	142 (29.04%)	163 (33.33%)	186 (38.04%)	267 (54.60%)
MDT recommendation	46 (9.40%)	55 (11.25%)	61 (12.47%)	74 (15.13%)	89 (18.20%)	197 (40.29%)

The table reports the accuracy for each model configuration, defined as exact match rates, reported as number of correct predictions with percentages in parentheses for treatment allocation, clinical case complexity assessment, and MDT recommendation decisions. Eight different language models were evaluated across six experimental conditions: zero-shot and few-shot prompting strategies applied to both unstructured clinical notes and structured data, with and without RAG. Results demonstrate progressive improvement in accuracy with structured data input, few-shot examples, and RAG integration across all models and tasks. MDT, multidisciplinary team; RAG, retrieval augmented generation.

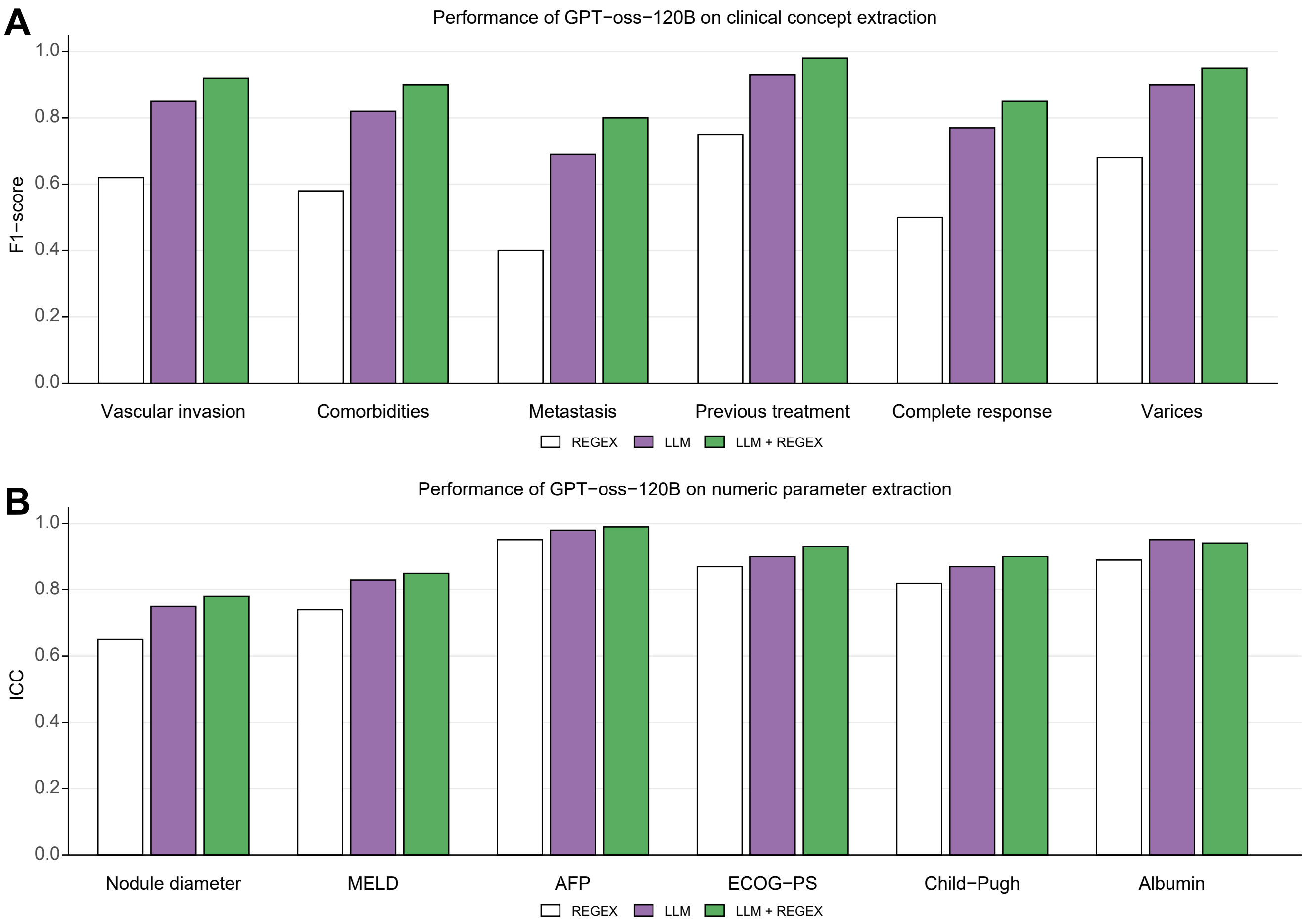

Fig. 3. Per-parameter extraction performance of the best-performing model (GPT-oss-120B+REGEX). Performance metrics for extraction of (A) categorical clinical concepts measured by F1 score and (B) numerical parameters measured by ICC. AFP, alpha-fetoprotein; ECOG-PS, Eastern Cooperative Oncology Group – performance status, ICC, intraclass correlation coefficient**;** MDT, multidisciplinary team; LLM, large language model; MELD, model for end-stage liver disease; REGEX, regular expression.

The magnitude of improvement – from 25-30% baseline accuracy to 80-88% with full PHENO-RAG implementation – underscores the critical importance of the use of pre-processed structured and the integration of data domain-specific knowledge, as already shown in other medical AI applications.[10,32] From a practical perspective, the structured data produced by this automated process enabled more accurate clinical decision-making compared to processing unstructured notes directly. This finding has important implications for clinical practice, as it suggests that automated systematic organization of clinical information may improve consistency and quality of treatment decisions. The ability to automatically convert narrative clinical reports into structured formats could therefore serve as a valuable tool for standardizing clinical workflows and supporting evidence-based decision-making. At the same time, incorporating current clinical guidelines directly into the decision-making process provided substantial performance gains. This approach, known as RAG, allows the system to automatically consult relevant sections of established treatment guidelines – similar to how clinicians reference current recommendations when making decisions. This ensures that treatment suggestions remain consistent with the latest evidence-based practices and can be updated as guidelines evolve. Our findings support evidence that LLM-RAG models can achieve high accuracy levels in specific clinical tasks when properly designed.[33]

Error analysis demonstrated that RAG systematically corrected the model's weakest performance areas: treatment categories with the lowest baseline accuracy (ablation, systemic therapy, follow-up) improved to 81-93% with guideline retrieval, while complexity assessment errors decreased from 200 to 56 cases with balanced directionality. This targeted improvement pattern suggests that guideline integration addresses specific knowledge gaps and systematic biases, strengthening confidence in the framework's clinical aim.

Among the three clinical tasks, MDT recommendation proved most challenging, with GPT-oss-120B achieving only 66.9% accuracy. This could reflect that current guidelines recommend universal or near-universal MDT discussion, while real-world practice requires selective prioritization due to resource constraints, creating discrepancy between guideline-based and actual decisions. Moreover, MDT referral incorporates subjective judgment and institutional factors that could not be captured in clinical notes.

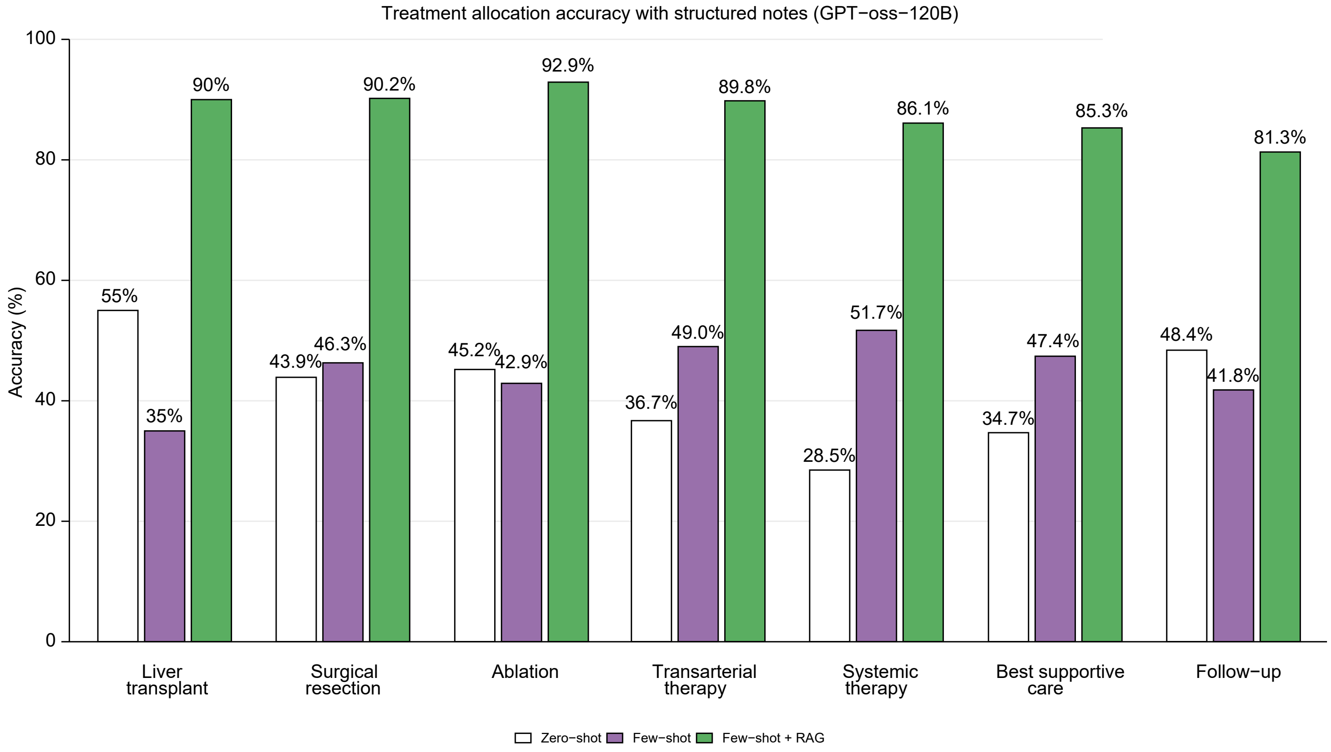

Fig. 4. Treatment allocation accuracy across prompting strategies for GPT-oss-120B. Bar chart illustrating the accuracy of treatment assignment for each management category when using zero-shot prompting, few-shot prompting, and few-shot prompting augmented with RAG on structured clinical notes. The integration of RAG consistently improved classification accuracy across all treatment categories, with the largest gains observed for systemic therapy, ablation, and transarterial therapy.

Our findings have important implications for the practical implementation of our framework as a tool for clinical decision support in HCC. First, they demonstrate the feasibility of using LLMs for complex clinical decision-making when properly configured with structured data (collected from a large sample of patients managed in a real-world setting at a tertiary referral center during a relatively short period) and domain-specific knowledge. Second, they highlight the importance of maintaining human oversight, particularly for complex cases requiring multidisciplinary input. Third, they suggest a potential role for LLMs in streamlining clinical workflows by identifying cases that would benefit most from MDT discussion, according to their clinical complexity.

Several limitations of our study should be acknowledged. First, while our dataset of 489 clinical reports represents a substantial sample, it comes from a single institution and may not capture the full variety of clinical presentations and decision-making patterns across different healthcare settings. Second, the retrospective nature of our study means that the model's recommendations require prospective validation. Third, we did not evaluate the predictive performance of the framework across different treatment modalities stratified by clinical complexity. Fourth, while our framework incorporated radiological information extracted from imaging reports embedded in clinical notes, it did not perform direct analysis of radiological images themselves, which are known to be fundamental determinants in therapeutic decision-making for HCC. The absence of this information could affect the ability of the model to fully capture the nuances that influence treatment allocation in clinical practice, particularly regarding the location of the tumor. Integrating direct radiological images in future iterations would potentially enhance the framework's accuracy and clinical applicability.

Future research directions should include prospective validation of the framework in clinical practice, evaluation across multiple institutions and healthcare systems, and investigation of how the framework performs with other complex diseases requiring multidisciplinary management. Additionally, further work is needed to improve the model's performance in borderline cases and to develop more robust methods for clinical complexity stratification.

Several ethical and practical considerations warrant discussion. First, while PHENO-RAG demonstrates promising accuracy, clinical liability remains with treating physicians. Second, transparent documentation of AI involvement in decision-making processes is essential for maintaining accountability and patient trust. Third, data confidentiality must be rigorously protected, particularly when implementing such frameworks in clinical practice; our use of locally hosted models rather than cloud-based application programming interfaces represents one approach to mitigating privacy risks.

In conclusion, our evaluation demonstrates the potential of multi-model LLM frameworks to support complex clinical decision-making in HCC management when properly implemented with structured data preprocessing, domain-specific knowledge retrieval, and systematic prompt optimization.

While these tools show promise in streamlining clinical workflows and supporting evidence-based practice, they should be viewed as augmentative technologies that enhance rather than replace clinical judgment and multidisciplinary expertise. Future research should focus on prospective validation, multimodal integration, and robust evaluation frameworks for complex clinical reasoning tasks.

Affiliations

[1]*Gastroenterology and Hepatology Unit, Department of Health Promotion, Mother & Child Care, Internal Medicine & Medical Specialties, University of Palermo, Palermo, Italy;* [2]*Department of Surgery & Cancer, Imperial College London, London, UK;* [3]*Department of Internal Medicine (Digestive Diseases), Yale School of Medicine, New Haven, Connecticut, USA;* [4]*Department of Medical, Surgical, and Health Sciences, University of Trieste, Trieste, Italy;* [5]*Department of Health Promotion, Mother & Child Care, Internal Medicine & Medical Specialties, University of Palermo, Palermo, Italy;* [6]*Department for the Treatment and the Study of Abdominal Diseases and Abdominal Transplantation, IRCCS-ISMETT (Istituto Mediterraneo per i Trapianti e Terapie ad alta specializzazione), UPMCI (University of Pittsburgh Medical Center Italy) Palermo, Italy;* [7]*University of Catania, Catania, Italy;* [8]*IRCCS-ISMETT (Istituto Mediterraneoper i Trapianti e Terapie ad alta specializzazione), UPMCI (University of Pittsburgh Medical Center Italy) Palermo, Italy;* [9]*Radiology Service, IRCCS-ISMETT (Istituto Mediterraneoper i Trapianti e Terapie ad alta specializzazione), UPMCI (University of Pittsburgh Medical Center Italy) Palermo, Italy;* [10]*Department of Biomedicine, Neuroscience, and Advanced Diagnostics (Bi.N.D.), University of Palermo, Palermo, Italy;* [11]*Department of Engineering and Architecture, University of Trieste, Italy;* [12]*Département Electronique-Electrotechnique-Automatique (EEA), University of Picardie Jules Verne, Amiens, France*

Abbreviations

AFP, alpha-fetoprotein; BSC, best supportive care; HCC, hepatocellular carcinoma; ICC, intraclass correlation coefficient; LLM, large language model; MELD, model for end-stage liver disease; MDT, multidisciplinary team; REGEX, regular expression; RAG, retrieval-augmented generation.

Financial support

The research leading to these results has received funding from the European Union - NextGenerationEU through the Italian Ministry of University and Research under PNRR M4C2I1.3 project PE_00000019 “HEAL ITALIA” CUPB73C22001250006 to Ca. C. M.G. is supported by the American-Italian Cancer Foundation Post-Doctoral Research Fellowship (Year 2024/2025).

Conflict of interest

The authors declare no conflicts of interest pertaining to this manuscript.
Please refer to the accompanying ICMJE disclosure forms for further details.

Authors’ contributions

Ci.C., and M.G. were involved in the conception of the study, the analysis and interpretation of the results and wrote the first draft of the manuscript. M.G., G.D.M, and S.K. were involved in formal analysis. G.C. and Ca.C.. were involved in the interpretation of the results and contributed to the discussion. R.C., M.S., A.G., S.R., G.G., A.Q., G.C., A.S., and V.G were involved in data collection. Calogero Cammà/Ca. C. is the guarantor. All authors edited, reviewed, and approved the final version of the manuscript.

Data availability

All supporting data are available within the article (and in the supplementary materials).

Code availability

Code can be provided based on personal requests. Please contact the corresponding author.

Supplementary data

Supplementary data to this article can be found online at https://doi.org/10.1016/j.jhepr.2025.101715.

References

[1] Bray F, Laversanne M, Sung H, et al. Global cancer statistics 2022: GLOBOCAN estimates of incidence and mortality worldwide for 36 cancers in 185 countries. CA Cancer J Clin 2024;74:229–263.

[2] Reig M, Cabibbo G. Antiviral therapy in the palliative setting of HCC (BCLC-B and -C). J Hepatol 2021;74:1225–1233.

[3] Vitale A, Cabibbo G, Iavarone M, et al. Personalised management of patients with hepatocellular carcinoma: a multiparametric therapeutic hierarchy concept. Lancet Oncol 2023;24:e312–e322.

[4] Cabibbo G, Daniele B, Borzio M, et al. Multidisciplinary treatment of hepatocellular carcinoma in 2023: Italian practice treatment guidelines of the Italian association for the study of the liver (AISF), Italian association of medical oncology (AIOM), Italian association of Hepato-Bilio-Pancreatic surgery (AICEP), Italian association of hospital gastroenterologists (AIGO), Italian association of radiology and clinical oncology (AIRO), Italian society of pathological anatomy and diagnostic cytology (SIAPeC-IAP), Italian society of surgery (SIC), Italian society of gastroenterology (SIGE), Italian society of medical and interventional radiology (SIRM), Italian organ transplant society (SITO), and association of patients with hepatitis and liver disease (EpaC) - Part I - surgical treatments. Dig Liver Dis 2024 Feb;56(2):223–234. https://doi.org/10.1016/j.dld.2023.10.029.

[5] Cabibbo G, Daniele B, Borzio M, et al. Multidisciplinary treatment of hepatocellular carcinoma in 2023: Italian practice treatment guidelines of the Italian association for the study of the liver (AISF), Italian association of medical oncology (AIOM), Italian association of hepato-Bilio-Pancreatic surgery (AICEP), Italian association of hospital gastroenterologists (AIGO), Italian association of radiology and clinical oncology (AIRO), Italian society of pathological anatomy and diagnostic cytology (SIAPeC-IAP), Italian society of surgery (SIC), Italian society of gastroenterology (SIGE), Italian society of medical and interventional radiology (SIRM), Italian organ transplant society (SITO), and association of patients with hepatitis and liver disease (EpaC) - Part II - non-surgical treatments. Dig Liver Dis 2024 Mar;56(3):394–405. https://doi.org/10.1016/j.dld.2023.10.028.

[6] Serper M, Taddei TH, Mehta R, et al. Association of provider specialty and multidisciplinary care with hepatocellular carcinoma treatment and mortality. Gastroenterology 2017;152:1954–1964.

[7] Sinn DH, Choi GS, Park HC, et al. Multidisciplinary approach is associated with improved survival of hepatocellular carcinoma patients. PLoS One 2019;14:e0210730.

[8] Yopp AC, Mansour JC, Beg MS, et al. Establishment of a multidisciplinary hepatocellular carcinoma clinic is associated with improved clinical outcome. Ann Surg Oncol 2014;21:1287–1295.

[9] Seif El Dahan K, Reczek A, Daher D, et al. Multidisciplinary care for patients with HCC: a systematic review and meta-analysis. Hepatol Commun 2023;7.

[10] Thirunavukarasu AJ, Ting DSJ, Elangovan K, et al. Large language models in medicine. Nat Med 2023;29:1930–1940.

[11] Singhal K, Azizi S, Tu T, et al. Large language models encode clinical knowledge. Nature 2023;620:172–180.

[12] Kresevic S, Giuffre M, Ajcevic M, et al. Optimization of hepatological clinical guidelines interpretation by large language models: a retrieval augmented generation-based framework. NPJ Digit Med 2024;7:102.

[13] Giuffre M, Kresevic S, Pugliese N, et al. Optimizing large language models in digestive disease: strategies and challenges to improve clinical outcomes. Liver Int 2024;44:2114–2124.

[14] Giuffre M, Kresevic S, You K, et al. Systematic review: the use of large language models as medical chatbots in digestive diseases. Aliment Pharmacol Ther 2024;60:144–166.

[15] European Association for the Study of the Liver. EASL clinical practice guidelines: management of hepatocellular carcinoma. J Hepatol 2018;69:182–236.

[16] Celsa C, Cabibbo G, Pagano D, et al. Sicily network for liver cancer: a multidisciplinary network model for the management of primary liver tumors. J Laparoendosc Adv Surg Tech A 2020;30:1048–1053.

[17] https://huggingface.co/meta-llama/Llama-3.1-8B-Instruct.

[18] https://huggingface.co/meta-llama/Llama-3.1-70B-Instruct.

[19] https://huggingface.co/openai/gpt-oss-20b.

[20] https://huggingface.co/openai/gpt-oss-120b.
[21] https://huggingface.co/Qwen/Qwen3-8B.
[22] https://huggingface.co/Qwen/Qwen3-Next-80B-A3B-Instruct.
[23] https://huggingface.co/tiiuae/falcon-7b.
[24] https://huggingface.co/tiiuae/falcon-40b.
[25] Giuffrè M, Distefano A, Kresevic S, et al. Guideline-enhanced large language models outperforms physician-test takers on EASL campus quizzes multiple choice questions. JHEP Rep 2025 Jul 14;7(10):101523. https://doi.org/10.1016/j.jhepr.2025.101523.
[26] Giuffre M, Pugliese N, Kresevic S, et al. From guidelines to real-time conversation: expert-validated retrieval-augmented and fine-tuned GPT-4 for hepatitis C management. Liver Int 2025;45:e70349.
[27] Giuffre M, You K, Pang Z, et al. Expert of experts verification and alignment (EVAL) framework for large language models safety in gastroenterology. NPJ Digit Med 2025;8:242.
[28] Xiong G, Jin Q, Lu Z, et al. Benchmarking retrieval-augmented generation for medicine. In: Findings of the association for computational linguistics ACL 2024; 2024.
[29] Zhao X, Liu S, Yang S-Y, et al. Medrag: enhancing retrieval-augmented generation with knowledge graph-elicited reasoning for healthcare copilot. In: Proceedings of the ACM on web conference 2025; 2025.
[30] Soroush A, Giuffre M, Chung S, et al. Generative artificial intelligence in clinical medicine and impact on gastroenterology. Gastroenterology 2025;169:502–517 e1.
[31] Yang Q, Jiang J, Dong X, et al. Performance of natural language processing model in extracting information from free-text radiology reports: a systematic review and meta-analysis. J Imaging Inform Med 2025 Oct 28. https://doi.org/10.1007/s10278-025-01728-8.
[32] Gaber F, Shaik M, Allega F, et al. Evaluating large language model workflows in clinical decision support for triage and referral and diagnosis. NPJ Digit Med 2025;8:263.
[33] Ke YH, Jin L, Elangovan K, et al. Retrieval augmented generation for 10 large language models and its generalizability in assessing medical fitness. NPJ Digit Med 2025;8:187.

Keywords: Large Language Models; Hepatocellular carcinoma; Retrieval Augmented Generation; Clinical Decision Support; Patient Phenotyping.

Received 22 July 2025; received in revised form 8 December 2025; accepted 15 December 2025; Available online 19 December 2025

Journal of Hepatology, Volume *8*

Supplemental information

PHENO-RAG: An artificial intelligence tool for guideline-informed management decisions in hepatocellular carcinoma

Ciro Celsa, Mauro Giuffrè, Gabriele Di Maria, Salvatore Gruttadauria, Ugo Palazzo, Roberto Miraglia, Luigi Maruzzelli, Duilio Pagano, Roberto Cannella, Federico Midiri, Roberta Ciccia, Mauro Salvato, Alessandro Grova, Sofia Rao, Gaetano Giusino, Alessio Quartararo, Guido Cusimano, Alba Sparacino, Valeria Gaudioso, Valeria Genovese, Rosangela Montenegro, Claudia La Mantia, Francesco Mercurio, Simone Kresevic, Milos Ajcevic, Giuseppe Cabibbo, Giansalvo Cirrincione, and Calogero Cammà

PHENO-RAG: An artificial intelligence tool for guideline-informed management decisions in hepatocellular carcinoma

Ciro Celsa, Mauro Giuffrè, Gabriele Di Maria, Salvatore Gruttadauria, Ugo Palazzo, Roberto Miraglia, Luigi Maruzzelli, Duilio Pagano, Roberto Cannella, Federico Midiri, Roberta Ciccia, Mauro Salvato, Alessandro Grova, Sofia Rao, Gaetano Giusino, Alessio Quartararo, Guido Cusimano, Alba Sparacino, Valeria Gaudioso, Valeria Genovese, Rosangela Montenegro, Claudia La Mantia, Francesco Mercurio, Simone Kresevic, Milos Ajcevic, Giuseppe Cabibbo, Giansalvo Cirrincione, Calogero Cammà

Table of contents

Definition of severe extrahepatic comorbidities………………………………..…………… 2

Criteria for MDT discussion……………………………………………………….……………2

Table S1……………………………………………………………………………………...….3

Composition of 100 cases used for the validation of clinical concept extraction approach…………………..…………………………………………………………..…………5

Table S2………………………………………………………………………………………….5

Table S3………………………………………………………………………………………….6

Prompt list for patient phenotyping……………………………………………………………7

Prompt list for treatment allocation…………………………………………………….….…17

Prompt list for case complexity determination……………………………………………...24

Fig. S1…………………………………………..………………………………….……………31

Fig. S2………………………………………………………………………..………………….32

Definition of severe extrahepatic comorbidities

Presence of at least one of the following: history of myocardial infarction, heart failure, peripheral vascular disease, stroke/ transient ischaemic attack (TIA), Chronic obstructive pulmonary disease (COPD), diabetes with end-organ damage, chronic kidney disease stage 4-5, active solid tumor, active leukemia/lymphoma, or Acquired Immune Deficiency Syndrome (AIDS).

Criteria for MDT discussion

PATIENTS REQUIRING MDT DISCUSSION:

1. Transplant candidates:
· Within Milan criteria
· Beyond Milan but within expanded criteria
· Downstaging candidates
2. Surgical resection candidates:
· Child-Pugh A with technically resectable tumor
· Selected Child-Pugh B or clinically significant portal hypertension with preserved liver function
3. Multiple treatment options available:
· Choice between resection vs. transplant
· Choice between ablation vs. resection
· Choice between resection vs intraarterial treatments
· Choice between ablation vs intraarterial treatments
· Bridge therapy selection for transplant candidates
4. Complex cases requiring careful evaluation:
· Borderline fitness for specific treatments
· Challenging tumor location
· Technical complexity
· Significant patient preferences affecting choice
· Relevant extrahepatic comorbidities impacting treatment selection

PATIENTS NOT REQUIRING MDT DISCUSSION:

1. Decompensated cirrhosis without transplant candidacy due to:
· Elderly age
· Severe extrahepatic comorbidities
· Tumor burden exceeding transplant criteria
2. Advanced HCC with:
· Extrahepatic spread
· Main portal vein invasion (Vp4)
· Clear systemic therapy indication

Table S1: Regex Dictionary for Phenotype Extraction.

Pattern Regex Alphafetoprotein:

```
Pattern_AFP =
r"(?i)(AFP|alfafetoproteina|alfa?feto(?:proteina)?|alfafeoproteina)\s*[=:]?\s*(\d+(?:[,.]\d+)?)"
```

Pattern Regex Comorbidities:

```
Pattern_Comorbidities = (
        r"(?i)(infarto|ischemia\s+miocardica|cardiopatia\s+ischemica|"
        r"scompenso\s+cardiaco|insufficienza\s+cardiaca|"
        r"vascolare\s+periferica|arteriopatia\s+periferica|"
        r"ictus|TIA|attacco\s+ischemico|"
        r"BPCO|broncopneumopatia\s+cronica\s+ostruttiva|enfisema|bronchite\s+cronica|"

r"diabete.*?(organo|complicanze)|retinopatia\s+diabetica|neuropatia\s+diabetica|nefropatia\s+diabetica|"
        r"insufficienza\s+renale.*?(stadio\s*4|stadio\s*5)|uremia|IRC\s+avanzata|"
        r"tumore\s+solido\s+attivo|neoplasia|carcinoma|metastasi|"
        r"leucemia|linfoma|mieloma|AIDS|HIV)"
)
```

Pattern Regex Metastases:

```
Pattern_Metastases_Negation = (
r"(?i)(assenza\s+di|non\s+presenta|esclusione\s+di|non\s+si\s+osservano|"
r"non\s+sono\s+presenti|priva\s+di|assenti|negativo\s+per)" )
Pattern_Regex_Metastases = (
r"(?i)(metastasi|secondarismi|localizzazioni\s+secondarie|diffusione|coinvolgimento).*?"
r"(polmon[ei]|oss[oae]|linfonod[oi]|peritoneo|pleura|surreni|reni|cervello|"
r"encefalo|midollo\s+spinale|vertebre|scheletro|torace|mediastino|"
r"extraepatic[ohea]|fuori\s+dal\s+fegato)" )
```

Pattern Regex Performance Status:

```
Pattern_Performance_Status = ( r"(?i)(PS|Performance\s+Status|[Ss]tatus\s+[Ff]unzionale|"
r"[Pp]erformance\s+[Ff]unzionale|ECOG)[\s:=-]*(\b[0-4]\b)" )
```

Pattern Regex Any Previous Treatment:

```
Pattern_Any_Previous_Treatment = (
r"(?i)(trapianto|chirurgia|resezion[ei]|TACE|ablazione|termoablazione|"
r"alcolizzazione|PEI|etanolo|alcol|TAE|TARE|sorafenib|regorafenib|"
r"lenvatinib|atezolizumab|bevacizumab|terapia\s+sistemica|radioterapia|"
r"chemioembolizzazione|embolizzazione|radioembolizzazione|immunoterapia|"
r"trattamento\s+chirurgico|intervento\s+chirurgico|terapia\s+locoregionale|"
r"chemioterapia|terapia\s+target|radio\s*frequenza|RF|microonde|MW)" )
```

Pattern Regex Complete Response Last Treatment:

```
Pattern_Regex_Complete_Response_Last_Treatment = (
        r"(?i)(trapianto|chirurgia|resezion[ei]|TACE|ablazione|termoablazione|"
        r"alcolizzazione|PEI|etanolo|alcol|TAE|TARE|sorafenib|regorafenib|"
        r"lenvatinib|atezolizumab|bevacizumab|terapia\s+sistemica|radioterapia|"
        r"chemioembolizzazione|embolizzazione|radioembolizzazione|immunoterapia|"
        r"trattamento\s+chirurgico|intervento\s+chirurgico|terapia\s+locoregionale|"
        r"chemioterapia|terapia\s+target|radio\s*frequenza|RF|microonde|MW).*?"
        r"(?:.*?(?:\b\d{1,2}[\/-]\d{1,2}[\/-](?:19|20)\d{2}\b|\b(?:19|20)\d{2}\b))?.*?"
        r"(risposta\s+completa|CR|complete\s+response|assenza\s+di\s+recidiva|"
        r"no\s+recidiva\s+di\s+malattia|assenza\s+di\s+progressione|"
        r"assenza\s+di\s+malattia|malattia\s+non\s+rilevabile|"
        r"remissione\s+completa|nessuna\s+progressione)"
    )
```

Regex Child-Pugh:

```
Pattern_Regex_Child-Pugh = [
    r"(?i)(?:Child[-\s]?Pugh|CP)[-\s]*(?:score|punteggio|classe)?[-\s]*(\b[5-9]|1[0-5]\b)",
    r"(?i)(\b[5-9]|1[0-5]\b)[-\s]*(?:points?|punti)?[-\s]*(?:Child[-\s]?Pugh|CP)",
    # Pattern per classe A/B/C con punteggio
    r"(?i)(?:Child[-\s]?Pugh|CP)[-\s]*(?:classe|class)?[-\s]*[ABC][-\s]*\((\b[5-9]|1[0-5]\b)\)",
    # Pattern per punteggio seguito da classe
    r"(?i)(?:punteggio|score)[-\s]*(?:Child[-\s]?Pugh|CP)[-\s]*(?:di)?[-\s]*(\b[5-9]|1[0-5]\b)"
  ]
```

Regex Esophageal Varices:

```
Pattern_Esophageal_Varices_Negation = (
    r"(?i)(non\s+sono\s+presenti|assenza\s+di|non\s+si\s+evidenziano|"
    r"non\s+si\s+rilevano|non\s+si\s+osservano|assenti|negative\s+per)"
  )
Pattern_Esophageal_Varices_Presence = (
    r"(?i)(varic[ei]\s+esofage[ea]|varic[ei]\s+gastro-?esofage[ea])"
    r"(?:\s+(?:di\s+)?(?:F[1-3]|[Gg]rado\s*[1-4]|[Pp]iccolo\s+[Cc]alibro|"
    r"[Mm]edio\s+[Cc]alibro|[Gg]rosso\s+[Cc]alibro))?"
  )
```

Regex Nodule Dimensions:

```
Pattern_Nodule_Dimension = (
    r"(?i)(nodulo|neoformazione|lesione|massa|formazione|HCC|lesioni\s+focali|"
    r"monofocale|S[1-8]|S4[ab]|tumefazione).*?"
    r"(?:di\s+)?(\d+(?:\.\d+)?(?:\s*[x×]\s*\d+(?:\.\d+)?)?)\s*(cm|mm)"
  )
```

Regex Portal_Vein_Invasion:

```
Pattern_Portal_Vein_Negation = re.compile(
    r"\b(assenza\s+di|no|non|assenza|non\s+evidenza\s+di|no\s+evidenza|"
    r"non\s+si\s+osservano|no\s+segni\s+di|non\s+evidenti|senza|privo\s+di|"
    r"non\s+trombosi|non\s+invasione|non\s+più\s+evidente|"
    r"non\s+apprezz\w+|non\s+rilevat[oa]|assenza\s+di\s+recidiva)\b",
    re.IGNORECASE
  )
Pattern_Portal_Vein_Normality = re.compile(
    r"\b(pervio|perviet[àa]|nella\s+norma|regolare|normale|invariat[oa])\b",
    re.IGNORECASE
  )

Pattern_Portal_Vein_Presence = re.compile(
    r"\b(trombosi\s+portale|trombosi\s+portale\s+neoplastica|"
    r"invasione\s+vascolare|invasione\s+vena\s+porta|"
    r"invasione\s+portale|infiltrazione\s+vascolare|infiltrazione\s+portale|"
    r"infiltrante|LR-TIV|trombosi\s+(vascolare|tumorale)|"
    r"ramo\s+(destro|sinistro)\s+della\s+vena\s+porta|"
    r"ramo\s+portale\s+(destro|sinistro)|"
    r"con\s+trombosi\s+portale|con\s+invasione\s+vascolare)\b",
    re.IGNORECASE
  )
```

Albumin:

```
Pattern_Albumin = [
r"(?i)(?:albumin[ae]|alb\.?)[\s:=]*(\d+(?:[.,]\d+)?)(?:\s*(?:g/[dD][lL]|g/[lL]|gr/dl|g%|gr%))",
r"(?i)(?:albumin[ae]|alb\.?)[\s:=]*(\d+(?:[.,]\d+)?)",
r"(?i)(?:esami[\s\w]+?|bioumor[\s\w]+?).*?(?:albumin[ae]|alb\.?)[\s:=]*(\d+(?:[.,]\d+)?)"
  ]
```

Composition of 100 cases used for the validation of clinical concept extraction approach

Among 100 cases used for validating clinical concept extraction, clinical decisions were represented by liver transplantation in 5 cases, surgical resection in 6, ablation in 6, transarterial treatments in 6, systemic therapy in 27, BSC in 24 and follow-up continuation in 26.
Case complexity was judged as low in 26 of cases, moderate in 41 and high in 33.

Table S2: Model-specific performance metrics for extraction of individual clinical concepts

Model Configuration	Metastasis	Complete Response	Comorbidities	Vascular Invasion	Varices	Previous Treatment
REGEX	0.40	0.50	0.58	0.62	0.68	0.75
Llama-3-8B	0.60	0.68	0.76	0.81	0.83	0.88
Llama-3-8B+REGEX	0.64	0.71	0.79	0.84	0.86	0.90
Llama-3-70B	0.66	0.73	0.82	0.87	0.88	0.92
Llama-3-70B+REGEX	0.72	0.79	0.86	0.90	0.91	0.95
GPT-oss-20B	0.63	0.70	0.78	0.83	0.85	0.89
GPT-oss-20B+REGEX	0.75	0.81	0.87	0.88	0.93	0.96
GPT-oss-120B	0.69	0.77	0.82	0.85	0.90	0.93
GPT-oss-120B+REGEX	0.80	0.85	0.90	0.92	0.95	0.98
Qwen-3-8B	0.58	0.65	0.73	0.79	0.85	0.87
Qwen-3-8B+REGEX	0.66	0.77	0.84	0.85	0.91	0.94
Qwen-3-80B	0.62	0.74	0.79	0.83	0.92	0.95
Qwen-3-80B+REGEX	0.70	0.80	0.85	0.88	0.93	0.96
Falcon-7B	0.52	0.60	0.63	0.65	0.71	0.75
Falcon-7B+REGEX	0.58	0.65	0.70	0.71	0.74	0.78
Falcon-40B	0.55	0.64	0.67	0.69	0.71	0.75
Falcon-40B+REGEX	0.60	0.66	0.72	0.73	0.78	0.82

Supplementary Table S2 reports the per-concept F1-scores for categorical clinical variables extracted from narrative clinical reports across all evaluated model configurations. The table includes performance for six key clinical concepts—metastatic disease, complete radiological response, severe comorbidities, vascular invasion, esophageal varices, and previous HCC treatment—representing the principal categorical features used for downstream clinical decision-support tasks. For each language-model family (Meta Llama-3, OpenAI GPT-oss, Qwen-3, and TII Falcon), both base models and their corresponding REGEX-augmented variants are presented alongside the standalone REGEX pipeline.

Table S3: Model-specific performance metrics for extraction of numerical parameters.

Model Configuration	Nodule Diameter	MELD	AFP	ECOG-PS	Child-Pugh	Albumin
REGEX	0.65	0.74	0.95	0.87	0.82	0.89
Llama-3-8B	0.60	0.67	0.90	0.81	0.77	0.86
Llama-3-8B+REGEX	0.63	0.71	0.90	0.84	0.79	0.85
Llama-3-70B	0.70	0.77	0.96	0.89	0.85	0.92
Llama-3-70B+REGEX	0.72	0.80	0.97	0.90	0.87	0.92
GPT-oss-20B	0.65	0.73	0.88	0.80	0.78	0.84
GPT-oss-20B+REGEX	0.66	0.71	0.91	0.85	0.80	0.87
GPT-oss-120B	0.75	0.83	0.98	0.90	0.87	0.95
GPT-oss-120B+REGEX	0.78	0.85	0.99	0.93	0.90	0.94
Qwen-3-8B	0.55	0.65	0.80	0.70	0.68	0.76
Qwen-3-8B+REGEX	0.58	0.69	0.88	0.79	0.75	0.83
Qwen-3-80B	0.57	0.65	0.86	0.76	0.72	0.81
Qwen-3-80B+REGEX	0.60	0.70	0.89	0.82	0.77	0.83
Falcon-7B	0.58	0.65	0.78	0.69	0.67	0.73
Falcon-7B+REGEX	0.59	0.64	0.80	0.72	0.70	0.75
Falcon-40B	0.62	0.74	0.89	0.82	0.79	0.83
Falcon-40B+REGEX	0.68	0.74	0.89	0.82	0.79	0.83

Supplementary Table S3 presents the intraclass correlation coefficients (ICC) for six numerical parameters extracted from clinical reports—nodule maximum diameter, MELD score, AFP levels, ECOG Performance Status, Child-Pugh score, and serum albumin. These metrics quantify agreement between automated extraction systems and human-annotated reference values, reflecting the accuracy of continuous variable retrieval.

****Prompt List for Patient Phenotyping****

Prompt Extraction Alpha-fetoprotein:

This prompt consists of three sections: “Instruction”, “Examples”, and “Input”. The “Instruction” section contains information on your task, the “Examples” section contains 10 examples of clinical notes from patients with Hepatocellular Carcinoma (HCC) and the relevant clinical note extracted; while the “Input” section contains a new clinical note from a patient with HCC, for which you need to extract the relevant information based on the provided “Instruction” and “Examples”. All the notes are in their original language (i.e., Italian), thus some word variations reported in the Instruction section are based on the original language of the clinical note.

Section 1: Instruction
Read the following clinical report and extract the numerical value of **Alpha-Fetoprotein (AFP)**. The value may be preceded or followed by the terms **AFP**, **alpha-feto**, **alfafetoproteina**, **alfa-fetoproteina**, or similar variations.
- The AFP value may be written in decimal format using either a **dot (4.4) or a comma (4,4)**. Both formats are considered equivalent.
- The AFP value is usually (but not exclusively) found in a section that begins with "Laboratory tests" or "Biochemical exams."
- **If the AFP value is present, respond ONLY with the number in dot-decimal format.**
- **If the AFP value is not reported, respond with `"Not reported"`.**
- **Do not include any additional text in your response.**

Section 2: Examples
Example N:
Clinical Report:
"{Example Clinical Note}"
Response: {Example of Value Extraction}

Section 3: Input
Clinical Report:
"{Patient_Data}"
Response:

Prompt Extraction Severe Comorbidities:

This prompt consists of three sections: “Instruction”, “Examples”, and “Input”. The “Instruction” section contains information on your task, the “Examples” section contains 10 examples of clinical notes from patients with Hepatocellular Carcinoma (HCC) and the relevant clinical note extracted; while the “Input” section contains a new clinical note from a patient with HCC, for which you need to extract the relevant information based on the provided “Instruction” and “Examples”. All the notes are in their original language (i.e., Italian), thus some word variations reported in the Instruction section are based on the original language of the clinical note.

Section 1: Instruction

Read the following clinical report and determine whether at least **one** of the following comorbidities is present. If **any** of the listed comorbidities are found, respond with `1`. Otherwise, respond with `0`. #### **Targeted Comorbidities:** - **History of myocardial infarction** (e.g., *infarto*, *ischemia miocardica*, *cardiopatia ischemica*) - **Heart failure** (e.g., *scompenso cardiaco*, *insufficienza cardiaca*) - **Peripheral vascular disease** (e.g., *vascolare periferica*, *arteriopatia periferica*) - **Stroke or transient ischemic attack (TIA)** (e.g., *ictus*, *TIA*, *attacco ischemico transitorio*) - **Chronic obstructive pulmonary disease (COPD)** (e.g., *BPCO*, *broncopneumopatia cronica ostruttiva*, *enfisema*, *bronchite cronica*) - **Diabetes with organ complications** (e.g., *diabete con complicanze d'organo*, *retinopatia diabetica*, *neuropatia diabetica*, *nefropatia diabetica*) - **Chronic kidney disease (stage 4 or 5)** (e.g., *insufficienza renale stadio 4 o 5*, *uremia*, *IRC avanzata*) - **Active solid tumor** (e.g., *tumore solido attivo*, *neoplasia*, *carcinoma*, *metastasi*) - **Active leukemia or lymphoma** (e.g., *leucemia*, *linfoma*, *mieloma*) - **AIDS/HIV** (e.g., *AIDS*, *HIV*)
Section 2: Examples
Example N:
Clinical Report:
"{Example Clinical Note}"
Response: {Example of Value Extraction}

Section 3: Input
Clinical Report:
"{Patient_Data}"
Response:

Prompt Extraction Metastases:

This prompt consists of three sections: "Instruction", "Examples", and "Input". The "Instruction" section contains information on your task, the "Examples" section contains 10 examples of clinical notes from patients with Hepatocellular Carcinoma (HCC) and the relevant clinical note extracted; while the "Input" section contains a new clinical note from a patient with HCC, for which you need to extract the relevant information based on the provided "Instruction" and "Examples". All the notes are in their original language (i.e., Italian), thus some word variations reported in the Instruction section are based on the original language of the clinical note.

Section 1: Instruction
Read the following clinical report and determine whether at least **one** of the following extrahepatic metastases (i.e., tumor spread beyond the liver) is present. If **any** of the listed metastatic sites are found, respond with `1`. Otherwise, respond with `0`. #### **Extrahepatic Metastases Locations:** - **Lungs** (e.g., *polmoni*, *polmone*) - **Bones** (e.g., *osso*, *ossa*, *scheletro*, *vertebre*, *midollo spinale*) - **Lymph nodes** (e.g., *linfonodi*, *linfonodale*, *mediastino*) - **Peritoneum** (e.g., *peritoneo*) - **Pleura** (e.g., *pleura*, *torace*) - **Adrenal glands** (e.g., *surreni*) - **Kidneys** (e.g., *reni*) - **Brain** (e.g., *cervello*, *encefalo*) - **Other locations beyond the liver** (e.g., *extraepatico*, *extraepatiche*, *fuori dal fegato*, *extrapatico*)
Section 2: Examples

Example N:
Clinical Report:
"{Example Clinical Note}"
Response: {Example of Value Extraction}

Section 3: Input
Clinical Report:
"{Patient_Data}"
Response:

Prompt Extraction Performance Status:

This prompt consists of three sections: “Instruction”, “Examples”, and “Input”. The “Instruction” section contains information on your task, the “Examples” section contains 10 examples of clinical notes from patients with Hepatocellular Carcinoma (HCC) and the relevant clinical note extracted; while the “Input” section contains a new clinical note from a patient with HCC, for which you need to extract the relevant information based on the provided “Instruction” and “Examples”. All the notes are in their original language (i.e., Italian), thus some word variations reported in the Instruction section are based on the original language of the clinical note.

Section 1: Instruction
Read the following clinical report and extract the **numerical value** of the **Performance Status (PS)**. The PS is an **integer between 0 and 4** that represents the patient's functional capacity. - The PS value may be written as **"PS"**, **"Performance Status"**, **"status funzionale"**, **"performance funzionale"**, or similar terms. - The PS value is always an **integer (0, 1, 2, 3, or 4)**. - **If the value is present, respond ONLY with the number.** - **If the value is not reported, respond with `"Not reported"`.** - **Do not include any additional text in your response.** If a match is found, extract the **numerical value (0-4)**. Otherwise, return `"Not reported"`.

Section 2: Examples
Example N:
Clinical Report:
"{Example Clinical Note}"
Response: {Example of Value Extraction}

Section 3: Input
Clinical Report:
"{Patient_Data}"
Response:

Prompt Extraction Portal Vein Invasion:

This prompt consists of three sections: “Instruction”, “Examples”, and “Input”. The “Instruction” section contains information on your task, the “Examples” section contains 10 examples of clinical notes from patients with Hepatocellular Carcinoma (HCC) and the relevant clinical note extracted; while the “Input” section contains a new clinical note from

a patient with HCC, for which you need to extract the relevant information based on the provided “Instruction” and “Examples”. All the notes are in their original language (i.e., Italian), thus some word variations reported in the Instruction section are based on the original language of the clinical note.

Section 1: Instruction
Read the following clinical report and determine whether there is **clear and non-negated evidence** of **tumor invasion of the portal vein** or **portal system**.
Criteria for Identifying Positive Cases (Portal Vein Invasion Present) Consider the presence of **portal vein invasion** only if there is a clear and **non-negated** mention of **at least one** of the following conditions: 1. **Neoplastic portal vein thrombosis** (*trombosi portale neoplastica*) 2. **Tumor or vascular thrombosis of the portal vein** (*trombosi portale tumorale o vascolare*) 3. **Tumor-related vascular invasion** (*invasione vascolare riferita a processo tumorale*) 4. **Portal vein invasion** (*invasione della vena porta*) 5. **Portal invasion** (*invasione portale*) 6. **Vascular or portal infiltration** (*infiltrazione vascolare o portale*) 7. **Presence of LR-TIV** 8. **Thrombosis or invasion of the right or left branch of the portal vein** (*ramo portale destro o sinistro con trombosi/invasione*) 9. **Thrombosis or infiltration of a portal branch (right or left)**
If **any** of these terms appear **without negation**, classify the case as `"1"`.
Criteria for Identifying Negative Cases (No Portal Vein Invasion) Consider the absence of **portal vein invasion** if **any** of the following conditions are mentioned: 1. **Portal system is patent** (*sistema portale pervio*) 2. **Portal vein is patent** (*vena porta pervia*) 3. **Portal system is normal** (*sistema portale nella norma*) 4. **Patent portal system** (*pervietà del sistema portale*) 5. **Absence of portal thrombosis** (*assenza di trombosi portale*) 6. **No evidence of thrombosis** (*non evidenza di trombosi*) 7. **Absence of vascular invasion** (*assenza di invasione vascolare*) 8. **No signs of infiltration** (*no segni di infiltrazione*) 9. **No observed invasion** (*non si osserva invasione*) 10. **Vascular system is normal or regular** (*sistema vascolare regolare/normale*) 11. **Follow-up shows normal findings** (*follow-up nella norma*) 12. **No recurrence is detected** (*assenza di recidiva*) 13. **Not evident anymore** (*non più evidente*) 14. **Not appreciable** (*non apprezzabile*) 15. **Invasion not detected** (*non rilevata invasione*) 16. **Portal system unchanged and previously normal** (*sistema portale invariato e precedentemente normale*) If **any** of these terms appear in the clinical note, classify the case as `"0"`.

Important Considerations - The **mere presence of words** like *"thrombosis"* or *"invasion"* is **not sufficient** if they are preceded by **negations**. - If the report describes a **normal follow-up** or **absence of recurrence**, classify the case as `"No"`. - **If in doubt or ambiguity exists, classify the case as `"No"`**.

Section 2: Examples
Example N:
Clinical Report:
"{Example Clinical Note}"
Response: {Example of Value Extraction}

Section 3: Input
Clinical Report:
"{Patient_Data}"
Response:

Prompt Extraction Previous HCC Treatment:

This prompt consists of three sections: “Instruction”, “Examples”, and “Input”. The “Instruction” section contains information on your task, the “Examples” section contains 10 examples of clinical notes from patients with Hepatocellular Carcinoma (HCC) and the relevant clinical note extracted; while the “Input” section contains a new clinical note from a patient with HCC, for which you need to extract the relevant information based on the provided “Instruction” and “Examples”. All the notes are in their original language (i.e., Italian), thus some word variations reported in the Instruction section are based on the original language of the clinical note.

Section 1: Instruction
Read the following clinical report and determine whether at least **one** of the following **HCC treatments** is present. If **any** of the listed treatments are found, respond with `1`. Otherwise, respond with `0`. Previous treatment refers to **any therapeutic intervention** the patient has received **at any point in time** for HCC, regardless of the outcome. This includes **surgical, locoregional, systemic, and radiation-based treatments**, as well as **targeted and immunotherapies**. The presence of a treatment is confirmed if there is a direct mention of a specific intervention in the clinical note. #### **HCC Treatments:** - **Liver transplant** (e.g., *trapianto*) - **Surgery or resection** (e.g., *chirurgia*, *resezione*, *resezioni*, *trattamento chirurgico*, *intervento chirurgico*) - **Transarterial chemoembolization (TACE)** (e.g., *TACE*, *chemioembolizzazione*) - **Ablation (e.g., radiofrequency or microwave ablation)** (e.g., *ablazione*, *termoablazione*) - **Transarterial embolization (TAE)** (e.g., *TAE*, *embolizzazione*) - **Transarterial radioembolization (TARE)** (e.g., *TARE*, *radioembolizzazione*) - **Systemic therapy** (e.g., *terapia sistemica*, *chemioterapia*, *terapia target*) - **Targeted therapy** (e.g., *sorafenib*, *regorafenib*, *lenvatinib*, *atezolizumab*, *bevacizumab*, *immunoterapia*) - **Radiotherapy** (e.g., *radioterapia*)

Section 2: Examples
Example N:
Clinical Report:
"{Example Clinical Note}"
Response: {Example of Value Extraction}

Section 3: Input
Clinical Report:

"{Patient_Data}"
Response:

Prompt Extraction Complete Response Last Treatment:

This prompt consists of three sections: “Instruction”, “Examples”, and “Input”. The “Instruction” section contains information on your task, the “Examples” section contains 10 examples of clinical notes from patients with Hepatocellular Carcinoma (HCC) and the relevant clinical note extracted; while the “Input” section contains a new clinical note from a patient with HCC, for which you need to extract the relevant information based on the provided “Instruction” and “Examples”. All the notes are in their original language (i.e., Italian), thus some word variations reported in the Instruction section are based on the original language of the clinical note.

Section 1: Instruction
Read the following clinical report and determine whether **esophageal varices** are present. If **any** mention of esophageal varices is found, respond with `"1"`. Otherwise, respond with `"0"`. **Criteria for Identifying Esophageal Varices:** The presence of esophageal varices is confirmed if the clinical note mentions **any of the following terms**: - **Explicit mention of esophageal varices** (e.g., *varici esofagee*) - **Classification grades** associated with varices: - **F1, F2, F3**
Section 2: Examples
Example N:
Clinical Report:
"{Example Clinical Note}"
Response: {Example of Value Extraction}

Section 3: Input
Clinical Report:
"{Patient_Data}"
Response:

Prompt Extraction Esophageal Varices:

This prompt consists of three sections: “Instruction”, “Examples”, and “Input”. The “Instruction” section contains information on your task, the “Examples” section contains 10 examples of clinical notes from patients with Hepatocellular Carcinoma (HCC) and the relevant clinical note extracted; while the “Input” section contains a new clinical note from a patient with HCC, for which you need to extract the relevant information based on the provided “Instruction” and “Examples”. All the notes are in their original language (i.e., Italian), thus some word variations reported in the Instruction section are based on the original language of the clinical note.

Section 1: Instruction
Read the following clinical report and determine whether **esophageal varices** are present. If **any** mention of esophageal varices is found, respond with `"1"`. Otherwise, respond with `"0"`. *Criteria for Identifying Esophageal Varices:** The presence of esophageal varices is confirmed if the clinical note mentions **any of the following

terms**: - **Explicit mention of esophageal varices** (e.g., *varici esofagee*) - **Classification grades** associated with varices: - **(F)1, (F)2, (F)3**

Section 2: Examples
Example N:
Clinical Report:
"{Example Clinical Note}"
Response: {Example of Value Extraction}

Section 3: Input
Clinical Report:
"{Patient_Data}"
Response:

Prompt Extraction Nodule Dimension:

This prompt consists of three sections: “Instruction”, “Examples”, and “Input”. The “Instruction” section contains information on your task, the “Examples” section contains 10 examples of clinical notes from patients with Hepatocellular Carcinoma (HCC) and the relevant clinical note extracted; while the “Input” section contains a new clinical note from a patient with HCC, for which you need to extract the relevant information based on the provided “Instruction” and “Examples”. All the notes are in their original language (i.e., Italian), thus some word variations reported in the Instruction section are based on the original language of the clinical note.

Section 1: Instruction
Read the following clinical report and extract the **largest reported size** of a **nodule, lesion, or mass** found in the **most recent imaging study (CT, MRI, or ultrasound)**.
Criteria for Extracting Nodule Size:
1. **Identify the most recent imaging study** in the clinical note. If multiple imaging reports are present, use the **latest one**. To determine the **most recent imaging report**, extract the **latest valid year** in the format: (19|20)\d{2}.
- If **multiple sizes are mentioned**, select the **largest** one reported in the latest imaging study.
- **If a nodule size is present, return the extracted size in the original format:**
2. **Locate any mention of a nodule, lesion, mass, or tumor**, including:
- **Nodule / Neoformation / Lesion / Mass** (e.g., *nodulo*, *neoformazione*, *lesione*, *massa*, *formazione*)
- **HCC lesion** (e.g., *HCC*, *lesioni focali*, *monofocale*)
- **Segment-specific lesions** (e.g., *S1*, *S2*, *S3*, *S4*, *S4a*, *S4b*, *S5*, *S6*, *S7*, *S8*)
3. **Extract the largest reported size**, formatted as:
- **Single value** (e.g., `"5 cm"`, `"11 mm"`)
- **Multiple dimensions** (e.g., `"11 x 8.8 cm"`, `"4.5 x 3.2 mm"`)
- **Ensure the extracted value includes the unit** (`cm` or `mm`).
4. **If no nodules or lesions are found, return `"No nodule found"`**.

Section 2: Examples
Example N:
Clinical Report:
"{Example Clinical Note}"
Response: {Example of Value Extraction}

Section 3: Input
Clinical Report:
"{Patient_Data}"
Response:

<u>Prompt Extraction Albumin:</u>

This prompt consists of three sections: “Instruction”, “Examples”, and “Input”. The “Instruction” section contains information on your task, the “Examples” section contains 10 examples of clinical notes from patients with Hepatocellular Carcinoma (HCC) and the relevant clinical note extracted; while the “Input” section contains a new clinical note from a patient with HCC, for which you need to extract the relevant information based on the provided “Instruction” and “Examples”. All the notes are in their original language (i.e., Italian), thus some word variations reported in the Instruction section are based on the original language of the clinical note.

Section 1: Instruction
Read the following clinical report and extract the **numerical value** of **albumin** if present.
Criteria for Extracting Albumin Value: 1. **Identify the albumin value**, which may be preceded or followed by: - **Albumina, alb, albumin, albumina sierica**, or similar terms. 2. **Albumin values may be reported in decimal format** using: - **Dot (4.4) or comma (4,4)** notation (both are considered equivalent). 3. **Units are typically**: - **g/dL, g/L, gr/dL, or g%**. 4. **If the albumin value is present, respond ONLY with the number in dot-decimal format (e.g., `4.4`).** 5. **If the albumin value is not reported, respond with `"Not reported"`.** 6. **Do not include any additional text in your response.**

Section 2: Examples
Example N:
Clinical Report:
"{Example Clinical Note}"
Response: {Example of Value Extraction}

Section 3: Input
Clinical Report:
"{Patient_Data}"
Response:

<u>Prompt Extraction MELD:</u>

This prompt consists of three sections: “Instruction”, “Examples”, and “Input”. The “Instruction” section contains information on your task, the “Examples” section contains 10 examples of clinical notes from patients with Hepatocellular Carcinoma (HCC) and the relevant clinical note extracted; while the “Input” section contains a new clinical note from a patient with HCC, for which you need to extract the relevant information based on the provided “Instruction” and “Examples”. All the notes are in their original language (i.e., Italian), thus some word variations reported in the Instruction section are based on the original language of the clinical note.

Section 1: Instruction
Read the following clinical report and extract the **numerical value** of the **MELD score** if present.
Criteria for Extracting MELD Score: 1. **Identify the MELD value**, which may be preceded by: - **MELD, MELD score, punteggio MELD, MELD-Na**, or similar terms. 2. **MELD values are typically integers** between **6 and 40**. 3. **If the MELD value is present, respond ONLY with the number.** 4. **If the MELD value is not reported, respond with `"Not reported"`.** 5. **Do not include any additional text in your response.**

Section 2: Examples
Example N:
Clinical Report:
"{Example Clinical Note}"
Response: {Example of Value Extraction}

Section 3: Input
Clinical Report:
"{Patient_Data}"
Response:

Prompt Extraction Child-Pugh:

This prompt consists of three sections: “Instruction”, “Examples”, and “Input”. The “Instruction” section contains information on your task, the “Examples” section contains 10 examples of clinical notes from patients with Hepatocellular Carcinoma (HCC) and the relevant clinical note extracted; while the “Input” section contains a new clinical note from a patient with HCC, for which you need to extract the relevant information based on the provided “Instruction” and “Examples”. All the notes are in their original language (i.e., Italian), thus some word variations reported in the Instruction section are based on the original language of the clinical note.

Section 1: Instruction
Read the following clinical report and extract the **Child-Pugh classification**, including both: 1. **The class (A, B, or C)** 2. **The numerical score (if available)**.
Criteria for Extracting the Child-Pugh Classification: - The classification may appear as: - **"Class A6", "Class B7 of Child-Pugh", "Class C", "CP Class B"** - Variants such as **"Child-Pugh A", "CP class B", "Child Pugh C"** are also valid. - **If the Child-Pugh class and score are present, return both** (e.g., `"B7"`). - **If only the Child-Pugh class is

present without a score, return only the letter** (e.g., `"B"`). - **If the classification is not reported, respond with `"Not reported"`**. - **Do not include any additional text in your response.**

Section 2: Examples
Example N:
Clinical Report:
"{Example Clinical Note}"
Response: {Example of Value Extraction}

Section 3: Input
Clinical Report:
"{Patient_Data}"
Response:

Prompt List for Treatment Allocation

Zero-Shot Prompt [Unstructured Note]:

This prompt consists of two sections: “Instruction”, and “Input”. The “Instruction” section contains information on your task, while the “Input” section contains a new clinical note from a patient with HCC, for which you need to determine the most appropriate treatment based on the provided provided“Instruction”.

Section 1: Instruction
You are a **hepatology expert specializing in the treatment of Hepatocellular Carcinoma (HCC)**. Read the following unstructured clinical report (uploaded in its original language – Italian) carefully and, based on the **patient’s characteristics and disease status**, determine the **most appropriate therapeutic option** from the list below:
A. **Liver Transplantation** (`TRAPIANTO EPATICO`)
B. **Surgical Resection** (`RESEZIONE CHIRURGICA`)
C. **Ablation** (`ABLAZIONE`) → Includes:
- **Microwave Ablation (MWA)** (*ablazione a microonde*)
- **Radiofrequency Ablation (RFA)** (*ablazione con radiofrequenza*)
- **Cryoablation** (*crioablazione*)
D. **Embolization** (`EMBOLIZZAZIONE`) → Includes:
- **Transarterial Chemoembolization (TACE)** (*chemoembolizzazione transarteriosa*)
- **Transarterial Radioembolization (TARE, also called SIRT)** (*radioembolizzazione transarteriosa, SIRT*)
- **Transarterial Embolization (TAE)** (*embolizzazione transarteriosa senza chemioterapia*)
E. **Systemic Therapy** (`TERAPIA SISTEMICA`) → Includes: **Sorafenib** - **Lenvatinib** - **Regorafenib** - **Cabozantinib** - **Atezolizumab + Bevacizumab** - **Durvalumab + Tremelimumab** - **Pembrolizumab** - **Nivolumab** -
F. **Best Supportive Care** (`BEST SUPPORTIVE CARE`) → **To be selected ONLY when all other therapeutic options (curative or palliative) have been exhausted.**: The disease is **too advanced** for locoregional or has progressed on second and third line systemic therapy. The clinical approach is limited to **symptom management and supportive care** rather than curative or life-prolonging strategies.
G. **Follow-up** (`FOLLOW-UP`) → **Only if the patient has achieved complete response (CR) and no active disease is present on the latest imaging.**

Response Format: - **If the most appropriate treatment is identified, return only the name of the treatment option** from the list above. - **Do not include any additional text in your response.**

Section 2: Input
Clinical Report:
"{Patient_Data}"
Response:

Zero-Shot Prompt [Structured Note]:

This prompt consists of two sections: “Instruction”, and “Input”. The “Instruction” section contains information on your task, while the “Input” section contains a new clinical note from a patient with HCC, for which you need to determine the most appropriate treatment based on the provided provided“Instruction”.

Section 1: Instruction
You are a **hepatology expert specializing in the treatment of Hepatocellular Carcinoma (HCC)**. Read the following structured clinical report (uploaded in its original language – Italian) carefully and, based on the **patient’s characteristics and disease status**, determine the **most appropriate therapeutic option** from the list below:
A. **Liver Transplantation** (`TRAPIANTO EPATICO`)
B. **Surgical Resection** (`RESEZIONE CHIRURGICA`)
C. **Ablation** (`ABLAZIONE`) → Includes:
- **Microwave Ablation (MWA)** (*ablazione a microonde*)
- **Radiofrequency Ablation (RFA)** (*ablazione con radiofrequenza*)
- **Cryoablation** (*crioablazione*)
D. **Embolization** (`EMBOLIZZAZIONE`) → Includes:
- **Transarterial Chemoembolization (TACE)** (*chemoembolizzazione transarteriosa*)
- **Transarterial Radioembolization (TARE, also called SIRT)** (*radioembolizzazione transarteriosa, SIRT*)
- **Transarterial Embolization (TAE)** (*embolizzazione transarteriosa senza chemioterapia*)
E. **Systemic Therapy** (`TERAPIA SISTEMICA`) → Includes: **Sorafenib** - **Lenvatinib** - **Regorafenib** - **Cabozantinib** - **Atezolizumab + Bevacizumab** - **Durvalumab + Tremelimumab** - **Pembrolizumab** - **Nivolumab** -
F. **Best Supportive Care** (`BEST SUPPORTIVE CARE`) → **To be selected ONLY when all other therapeutic options (curative or palliative) have been exhausted.**: The disease is **too advanced** for locoregional or has progressed on second and third line systemic therapy. The clinical approach is limited to **symptom management and supportive care** rather than curative or life-prolonging strategies.
G. **Follow-up** (`FOLLOW-UP`) → **Only if the patient has achieved complete response (CR) and no active disease is present on the latest imaging.**

Response Format: - **If the most appropriate treatment is identified, return only the name of the treatment option** from the list above. - **Do not include any additional text in your response.**

Section 2: Input
Clinical Report:
"{Patient_Data}"
Response:

Few-Shot Prompt [Unstructured Note]:

This prompt consists of three sections: “Instruction”, “Examples”, and “Input”. The “Instruction” section contains information on your task, the “Examples” section contains 10 examples of clinical notes from patients with Hepatocellular Carcinoma (HCC) and the relevant treatment selection; while the “Input” section contains a new clinical note from a patient with HCC, for which you need to determine the most appropriate treatment based on the provided “Instruction” and “Examples.”

Section 1: Instruction
You are a **hepatology expert specializing in the treatment of Hepatocellular Carcinoma (HCC)**. Read the following unstructured clinical report (uploaded in its original language – Italian) carefully and, based on the **patient’s characteristics and disease status**, determine the **most appropriate therapeutic option** from the list below:
A. **Liver Transplantation** (`TRAPIANTO EPATICO`)
B. **Surgical Resection** (`RESEZIONE CHIRURGICA`)
C. **Ablation** (`ABLAZIONE`) → Includes:
- **Microwave Ablation (MWA)** (*ablazione a microonde*)
- **Radiofrequency Ablation (RFA)** (*ablazione con radiofrequenza*)
- **Cryoablation** (*crioablazione*)
D. **Embolization** (`EMBOLIZZAZIONE`) → Includes:
- **Transarterial Chemoembolization (TACE)** (*chemoembolizzazione transarteriosa*)
- **Transarterial Radioembolization (TARE, also called SIRT)** (*radioembolizzazione transarteriosa, SIRT*)
- **Transarterial Embolization (TAE)** (*embolizzazione transarteriosa senza chemioterapia*)
E. **Systemic Therapy** (`TERAPIA SISTEMICA`) → Includes: **Sorafenib** - **Lenvatinib** - **Regorafenib** - **Cabozantinib** - **Atezolizumab + Bevacizumab** - **Durvalumab + Tremelimumab** - **Pembrolizumab** - **Nivolumab** -
F. **Best Supportive Care** (`BEST SUPPORTIVE CARE`) → **To be selected ONLY when all other therapeutic options (curative or palliative) have been exhausted.**: The disease is **too advanced** for locoregional or has progressed on second and third line systemic therapy. The clinical approach is limited to **symptom management and supportive care** rather than curative or life-prolonging strategies.
G. **Follow-up** (`FOLLOW-UP`) → **Only if the patient has achieved complete response (CR) and no active disease is present on the latest imaging.**

Response Format: - **If the most appropriate treatment is identified, return only the name of the treatment option** from the list above. - **Do not include any additional text in your response.**

Section 2: Examples
Example N:
Clinical Report:
"{Example Clinical Note}"
Response: {Treatment Allocation}
Section 3: Input

Clinical Report:
"{Patient_Data}"
Response:

Few-Shot Prompt [Structured Note]:

This prompt consists of three sections: “Instruction”, “Examples”, and “Input”. The “Instruction” section contains information on your task, the “Examples” section contains 10 examples of clinical notes from patients with Hepatocellular Carcinoma (HCC) and the relevant treatment selection; while the “Input” section contains a new clinical note from a patient with HCC, for which you need to determine the most appropriate treatment based on the provided “Instruction” and “Examples.”

Section 1: Instruction
You are a **hepatology expert specializing in the treatment of Hepatocellular Carcinoma (HCC)**. Read the following Structured clinical report (uploaded in its original language – Italian) carefully and, based on the **patient’s characteristics and disease status**, determine the **most appropriate therapeutic option** from the list below:
A. **Liver Transplantation** (`TRAPIANTO EPATICO`)
B. **Surgical Resection** (`RESEZIONE CHIRURGICA`)
C. **Ablation** (`ABLAZIONE`) → Includes:
- **Microwave Ablation (MWA)** (*ablazione a microonde*)
- **Radiofrequency Ablation (RFA)** (*ablazione con radiofrequenza*)
- **Cryoablation** (*crioablazione*)
D. **Embolization** (`EMBOLIZZAZIONE`) → Includes:
- **Transarterial Chemoembolization (TACE)** (*chemoembolizzazione transarteriosa*)
- **Transarterial Radioembolization (TARE, also called SIRT)** (*radioembolizzazione transarteriosa, SIRT*)
- **Transarterial Embolization (TAE)** (*embolizzazione transarteriosa senza chemioterapia*)
E. **Systemic Therapy** (`TERAPIA SISTEMICA`) → Includes: **Sorafenib** - **Lenvatinib** - **Regorafenib** - **Cabozantinib** - **Atezolizumab + Bevacizumab** - **Durvalumab + Tremelimumab** - **Pembrolizumab** - **Nivolumab** -
F. **Best Supportive Care** (`BEST SUPPORTIVE CARE`) → **To be selected ONLY when all other therapeutic options (curative or palliative) have been exhausted.**: The disease is **too advanced** for locoregional or has progressed on second and third line systemic therapy. The clinical approach is limited to **symptom management and supportive care** rather than curative or life-prolonging strategies.
G. **Follow-up** (`FOLLOW-UP`) → **Only if the patient has achieved complete response (CR) and no active disease is present on the latest imaging.**

Response Format: - **If the most appropriate treatment is identified, return only the name of the treatment option** from the list above. - **Do not include any additional text in your response.**

Section 2: Examples
Example N:

Clinical Report:
"{Example Clinical Note}"
Response: {Treatment Allocation}

Section 3: Input
Clinical Report:
"{Patient_Data}"
Response:

Few-Shot+RAG Prompt [Unstructured Note]:
This prompt consists of four sections: “Instruction”, “Examples”, “Guidelines”, and “Input”. The “Instruction” section contains information on your task, the “Examples” section contains 10 examples of clinical notes from patients with Hepatocellular Carcinoma (HCC) and the relevant treatment selection; the “Guidelines” section provides text chunks from the latest guidelines, which contain strict recommendations that determine the correct treatment allocation - these guidelines must be used in addition to the provided examples when selecting the most appropriate treatment; while the “Input” section contains a new clinical note from a patient with HCC, for which you need to determine the most appropriate treatment based on the provided “Instruction”, “Examples”, and “Guidelines”.

Section 1: Instruction
You are a **hepatology expert specializing in the treatment of Hepatocellular Carcinoma (HCC)**. Read the following unstructured clinical report (uploaded in its original language – Italian) carefully and, based on the **patient’s characteristics and disease status**, determine the **most appropriate therapeutic option** from the list below:
A. **Liver Transplantation** (`TRAPIANTO EPATICO`)
B. **Surgical Resection** (`RESEZIONE CHIRURGICA`)
C. **Ablation** (`ABLAZIONE`) → Includes:
- **Microwave Ablation (MWA)** (*ablazione a microonde*)
- **Radiofrequency Ablation (RFA)** (*ablazione con radiofrequenza*)
- **Cryoablation** (*crioablazione*)
D. **Embolization** (`EMBOLIZZAZIONE`) → Includes:
- **Transarterial Chemoembolization (TACE)** (*chemoembolizzazione transarteriosa*)
- **Transarterial Radioembolization (TARE, also called SIRT)** (*radioembolizzazione transarteriosa, SIRT*)
- **Transarterial Embolization (TAE)** (*embolizzazione transarteriosa senza chemioterapia*)
E. **Systemic Therapy** (`TERAPIA SISTEMICA`) → Includes: **Sorafenib** - **Lenvatinib** - **Regorafenib** - **Cabozantinib** - **Atezolizumab + Bevacizumab** - **Durvalumab + Tremelimumab** - **Pembrolizumab** - **Nivolumab** -
F. **Best Supportive Care** (`BEST SUPPORTIVE CARE`) → **To be selected ONLY when all other therapeutic options (curative or palliative) have been exhausted.**: The disease is **too advanced** for locoregional or has progressed on second and third line

systemic therapy. The clinical approach is limited to **symptom management and supportive care** rather than curative or life-prolonging strategies.
G. **Follow-up** (`FOLLOW-UP`) → **Only if the patient has achieved complete response (CR) and no active disease is present on the latest imaging.**

Response Format: - **If the most appropriate treatment is identified, return only the name of the treatment option** from the list above. - **Do not include any additional text in your response.**

Section 2: Examples
Example N:
Clinical Report:
"{Example Clinical Note}"
Response: {Treatment Allocation}

Section 3: Guidelines
The following text chunks are extracted from the latest guidelines related to HCC treatment and provide strict recommendations for treatment allocation. These must be used in conjunction with the examples above to determine the correct treatment.

Section 4: Input
Clinical Report:
"{Patient_Data}"
Response:

Few-Shot+RAG Prompt [Structured Note]:

This prompt consists of four sections: “Instruction”, “Examples”, “Guidelines”, and “Input”. The “Instruction” section contains information on your task, the “Examples” section contains 10 examples of clinical notes from patients with Hepatocellular Carcinoma (HCC) and the relevant treatment selection; the “Guidelines” section provides text chunks from the latest guidelines, which contain strict recommendations that determine the correct treatment allocation - these guidelines must be used in addition to the provided examples when selecting the most appropriate treatment; while the “Input” section contains a new clinical note from a patient with HCC, for which you need to determine the most appropriate treatment based on the provided “Instruction”, “Examples”, and “Guidelines”.

Section 1: Instruction
You are a **hepatology expert specializing in the treatment of Hepatocellular Carcinoma (HCC)**. Read the following Structured clinical report (uploaded in its original language – Italian) carefully and, based on the **patient’s characteristics and disease status**, determine the **most appropriate therapeutic option** from the list below:
A. **Liver Transplantation** (`TRAPIANTO EPATICO`)
B. **Surgical Resection** (`RESEZIONE CHIRURGICA`)
C. **Ablation** (`ABLAZIONE`) → Includes:
- **Microwave Ablation (MWA)** (*ablazione a microonde*)

- **Radiofrequency Ablation (RFA)** (*ablazione con radiofrequenza*)
- **Cryoablation** (*crioablazione*)
D. **Embolization** (`EMBOLIZZAZIONE`) → Includes:
- **Transarterial Chemoembolization (TACE)** (*chemoembolizzazione transarteriosa*)
- **Transarterial Radioembolization (TARE, also called SIRT)** (*radioembolizzazione transarteriosa, SIRT*)
- **Transarterial Embolization (TAE)** (*embolizzazione transarteriosa senza chemioterapia*)
E. **Systemic Therapy** (`TERAPIA SISTEMICA`) → Includes: **Sorafenib** - **Lenvatinib** - **Regorafenib** - **Cabozantinib** - **Atezolizumab + Bevacizumab** - **Durvalumab + Tremelimumab** - **Pembrolizumab** - **Nivolumab** -
F. **Best Supportive Care** (`BEST SUPPORTIVE CARE`) → **To be selected ONLY when all other therapeutic options (curative or palliative) have been exhausted.**: The disease is **too advanced** for locoregional or has progressed on second and third line systemic therapy. The clinical approach is limited to **symptom management and supportive care** rather than curative or life-prolonging strategies.
G. **Follow-up** (`FOLLOW-UP`) → **Only if the patient has achieved complete response (CR) and no active disease is present on the latest imaging.**

Response Format: - **If the most appropriate treatment is identified, return only the name of the treatment option** from the list above. - **Do not include any additional text in your response.**

Section 2: Examples
Example N:
Clinical Report:
"{Example Clinical Note}"
Response: {Treatment Allocation}

Section 3: Guidelines
The following text chunks are extracted from the latest guidelines related to HCC treatment and provide strict recommendations for treatment allocation. These must be used in conjunction with the examples above to determine the correct treatment.

Section 4: Input
Clinical Report:
"{Patient_Data}"
Response:

Prompt List for Case Complexity Determination

Zero-Shot Prompt [Unstructured Note]:

This prompt consists of two sections: “Instruction” and “Input”. The “Instruction” section provides details on your task, including the definitions of clinical complexity. - The “Input” section contains a new clinical note from a patient with HCC, for which you need to determine the “clinical complexity level” based on treatment allocation difficulty.

Section 1: Instruction
You are a hepatology expert specializing in the treatment of Hepatocellular Carcinoma (HCC). Read the following unstructured clinical report (uploaded in its original language – Italian) carefully and determine the clinical complexity level for treatment allocation, according to the definitions below.
Definition of Clinical Complexity in Treatment Allocation:

A. **Low Complexity (`LOW`)** → The optimal treatment allocation is **straightforward** and follows clear guideline recommendations, with no ambiguity.
B. **Moderate Complexity (`MODERATE`)** → Some elements of **uncertainty** exist, where **multiple treatment options** could be considered, but a **preferred pathway** can still be identified based on guideline interpretation.
C. **High Complexity (`HIGH`)** → The case falls into **"grey areas"** of current guidelines, where: - **Multiple therapeutic options hold comparable validity**, OR - **Patient characteristics introduce significant uncertainty** in applying standard treatment algorithms.

Important Considerations: - Complexity is assessed **only based on treatment allocation difficulty** (not overall disease severity or number of comorbidities). - If guideline-based treatment selection is **clear and direct**, classify as `LOW`. - If **some debate exists** but a clear treatment choice is still possible, classify as `MODERATE`. - If the case **does not fit standard treatment pathways** or involves **significant uncertainty**, classify as `HIGH`.

Response Format: - **Return only one of the following labels: `"LOW"`, `"MODERATE"`, or `"HIGH"`**. - **Do not include any additional text in your response.**

Section 2: Input
Clinical Report:
"{Patient_Data}"
Response:

Zero-Shot Prompt [Structured Note]:

This prompt consists of two sections: “Instruction” and “Input”. The “Instruction” section provides details on your task, including the definitions of clinical complexity. - The “Input” section contains a new clinical note from a patient with HCC, for which you need to determine the “clinical complexity level” based on treatment allocation difficulty.

Section 1: Instruction
You are a hepatology expert specializing in the treatment of Hepatocellular Carcinoma (HCC). Read the following Structured clinical report (uploaded in its original language – Italian) carefully and determine the clinical complexity level for treatment allocation, according to the definitions below.
Definition of Clinical Complexity in Treatment Allocation:

A. **Low Complexity (`LOW`)** → The optimal treatment allocation is **straightforward** and follows clear guideline recommendations, with no ambiguity.
B. **Moderate Complexity (`MODERATE`)** → Some elements of **uncertainty** exist, where **multiple treatment options** could be considered, but a **preferred pathway** can still be identified based on guideline interpretation.
C. **High Complexity (`HIGH`)** → The case falls into **"grey areas"** of current guidelines, where: - **Multiple therapeutic options hold comparable validity**, OR - **Patient characteristics introduce significant uncertainty** in applying standard treatment algorithms.

Important Considerations: - Complexity is assessed **only based on treatment allocation difficulty** (not overall disease severity or number of comorbidities). - If guideline-based treatment selection is **clear and direct**, classify as `LOW`. - If **some debate exists** but a clear treatment choice is still possible, classify as `MODERATE`. - If the case **does not fit standard treatment pathways** or involves **significant uncertainty**, classify as `HIGH`.

Response Format: - **Return only one of the following labels: `"LOW"`, `"MODERATE"`, or `"HIGH"`**. - **Do not include any additional text in your response.**

Section 2: Input
Clinical Report:
"{Patient_Data}"
Response:

Few-Shot Prompt [Unstructured Note]:

This prompt consists of three sections: “Instruction”, “Examples”, and “Input”. The Instruction section provides details on your task, including the definitions of clinical complexity. The “Examples” section includes clinical notes with corresponding complexity classifications to illustrate the classification process. The “Input” section contains a new clinical note from a patient with HCC, for which you need to determine the **clinical complexity level** based on treatment allocation difficulty.

Section 1: Instruction
You are a hepatology expert specializing in the treatment of Hepatocellular Carcinoma (HCC). Read the following unstructured clinical report (uploaded in its original language – Italian) carefully and determine the clinical complexity level for treatment allocation, according to the definitions below.
Definition of Clinical Complexity in Treatment Allocation:

A. **Low Complexity (`LOW`)** → The optimal treatment allocation is **straightforward** and follows clear guideline recommendations, with no ambiguity.
B. **Moderate Complexity (`MODERATE`)** → Some elements of **uncertainty** exist, where **multiple treatment options** could be considered, but a **preferred pathway** can still be identified based on guideline interpretation.
C. **High Complexity (`HIGH`)** → The case falls into **"grey areas"** of current guidelines, where: - **Multiple therapeutic options hold comparable validity**, OR - **Patient characteristics introduce significant uncertainty** in applying standard treatment algorithms.

Important Considerations: - Complexity is assessed **only based on treatment allocation difficulty** (not overall disease severity or number of comorbidities). - If guideline-based treatment selection is **clear and direct**, classify as `LOW`. - If **some debate exists** but a clear treatment choice is still possible, classify as `MODERATE`. - If the case **does not fit standard treatment pathways** or involves **significant uncertainty**, classify as `HIGH`.

Response Format: - **Return only one of the following labels: `"LOW"`, `"MODERATE"`, or `"HIGH"`**. - **Do not include any additional text in your response.**

Section 2: Examples
Example N:
Clinical Report:
"{Example Clinical Note}"
Response: {Clinical Complexity Level}

Section 3: Input
Clinical Report:
"{Patient_Data}"
Response:

Few-Shot Prompt [Structured Note]:

This prompt consists of three sections: “Instruction”, “Examples”, and “Input”. The Instruction section provides details on your task, including the definitions of clinical complexity. The “Examples” section includes clinical notes with corresponding complexity classifications to illustrate the classification process. The “Input” section contains a new clinical note from a patient with HCC, for which you need to determine the **clinical complexity level** based on treatment allocation difficulty.

Section 1: Instruction
You are a hepatology expert specializing in the treatment of Hepatocellular Carcinoma (HCC). Read the following Structured clinical report (uploaded in its original language – Italian) carefully and determine the clinical complexity level for treatment allocation, according to the definitions below.
Definition of Clinical Complexity in Treatment Allocation:

A. **Low Complexity (`LOW`)** → The optimal treatment allocation is **straightforward** and follows clear guideline recommendations, with no ambiguity.
B. **Moderate Complexity (`MODERATE`)** → Some elements of **uncertainty** exist, where **multiple treatment options** could be considered, but a **preferred pathway** can still be identified based on guideline interpretation.
C. **High Complexity (`HIGH`)** → The case falls into **"grey areas"** of current guidelines, where: - **Multiple therapeutic options hold comparable validity**, OR - **Patient characteristics introduce significant uncertainty** in applying standard treatment algorithms.

Important Considerations: - Complexity is assessed **only based on treatment allocation difficulty** (not overall disease severity or number of comorbidities). - If guideline-based treatment selection is **clear and direct**, classify as `LOW`. - If **some debate exists** but a clear treatment choice is still possible, classify as `MODERATE`. - If the case **does not fit standard treatment pathways** or involves **significant uncertainty**, classify as `HIGH`.

Response Format: - **Return only one of the following labels: `"LOW"`, `"MODERATE"`, or `"HIGH"`**. - **Do not include any additional text in your response.**

Section 2: Examples
Example N:
Clinical Report:
"{Example Clinical Note}"
Response: {Clinical Complexity Level}

Section 3: Input
Clinical Report:
"{Patient_Data}"
Response:

Few-Shot+RAG Prompt [Unstructured Note]:

This prompt consists of four sections: “Instruction”, “Examples”, “Guidelines”, and “Input”. - The “Instruction” section provides details on your task, including the definitions of clinical complexity. The “Examples” section includes clinical notes with corresponding complexity classifications to illustrate the classification process. The “Guidelines” section provides text chunks from the latest guidelines, which contain strict recommendations that influence treatment allocation complexity. These guidelines must be used in addition to the provided examples when determining the complexity level. The “Input” section contains a new clinical note from a patient with HCC, for which you need to determine the **clinical complexity level** based on treatment allocation difficulty.

Section 1: Instruction
You are a hepatology expert specializing in the treatment of Hepatocellular Carcinoma (HCC). Read the following unstructured clinical report (uploaded in its original language – Italian) carefully and determine the clinical complexity level for treatment allocation, according to the definitions below.
Definition of Clinical Complexity in Treatment Allocation:

A. **Low Complexity (`LOW`)** → The optimal treatment allocation is **straightforward** and follows clear guideline recommendations, with no ambiguity.
B. **Moderate Complexity (`MODERATE`)** → Some elements of **uncertainty** exist, where **multiple treatment options** could be considered, but a **preferred pathway** can still be identified based on guideline interpretation.
C. **High Complexity (`HIGH`)** → The case falls into **"grey areas"** of current guidelines, where: - **Multiple therapeutic options hold comparable validity**, OR - **Patient characteristics introduce significant uncertainty** in applying standard treatment algorithms.

Important Considerations: - Complexity is assessed **only based on treatment allocation difficulty** (not overall disease severity or number of comorbidities). - If guideline-based treatment selection is **clear and direct**, classify as `LOW`. - If **some debate exists** but a clear treatment choice is still possible, classify as `MODERATE`. - If the case **does not fit standard treatment pathways** or involves **significant uncertainty**, classify as `HIGH`.

Response Format: - **Return only one of the following labels: `"LOW"`, `"MODERATE"`, or `"HIGH"`**. - **Do not include any additional text in your response.**

Section 2: Examples
Example N:
Clinical Report:
"{Example Clinical Note}"
Response: {Clinical Complexity Level}

Section 3: Guidelines

The following text chunks are extracted from the latest guidelines related to HCC treatment and provide strict recommendations for treatment allocation. These must be used in conjunction with the examples above to determine the correct case complexity.

Section 4: Input
Clinical Report:
"{Patient_Data}"
Response:

Few-Shot+RAG Prompt [Structured Note]:
This prompt consists of four sections: “Instruction”, “Examples”, “Guidelines”, and “Input”. - The “Instruction” section provides details on your task, including the definitions of clinical complexity. The “Examples” section includes clinical notes with corresponding complexity classifications to illustrate the classification process. The “Guidelines” section provides text chunks from the latest guidelines, which contain strict recommendations that influence treatment allocation complexity. These guidelines must be used in addition to the provided examples when determining the complexity level. The “Input” section contains a new clinical note from a patient with HCC, for which you need to determine the **clinical complexity level** based on treatment allocation difficulty.

Section 1: Instruction
You are a hepatology expert specializing in the treatment of Hepatocellular Carcinoma (HCC). Read the following Structured clinical report (uploaded in its original language – Italian) carefully and determine the clinical complexity level for treatment allocation, according to the definitions below.
Definition of Clinical Complexity in Treatment Allocation:

A. **Low Complexity (`LOW`)** → The optimal treatment allocation is **straightforward** and follows clear guideline recommendations, with no ambiguity.
B. **Moderate Complexity (`MODERATE`)** → Some elements of **uncertainty** exist, where **multiple treatment options** could be considered, but a **preferred pathway** can still be identified based on guideline interpretation.
C. **High Complexity (`HIGH`)** → The case falls into **"grey areas"** of current guidelines, where: - **Multiple therapeutic options hold comparable validity**, OR - **Patient characteristics introduce significant uncertainty** in applying standard treatment algorithms.

Important Considerations: - Complexity is assessed **only based on treatment allocation difficulty** (not overall disease severity or number of comorbidities). - If guideline-based treatment selection is **clear and direct**, classify as `LOW`. - If **some debate exists** but a clear treatment choice is still possible, classify as `MODERATE`. - If the case **does not fit standard treatment pathways** or involves **significant uncertainty**, classify as `HIGH`.

Response Format: - **Return only one of the following labels: `"LOW"`, `"MODERATE"`, or `"HIGH"`**. - **Do not include any additional text in your response.**

Section 2: Examples
Example N:
Clinical Report:
"{Example Clinical Note}"
Response: {Clinical Complexity Level}

Section 3: Guidelines
The following text chunks are extracted from the latest guidelines related to HCC treatment and provide strict recommendations for treatment allocation. These must be used in conjunction with the examples above to determine the correct case complexity.

Section 4: Input
Clinical Report:
"{Patient_Data}"
Response:

Fig. S1: Confusion matrix for treatment allocation by GPT-oss-120B with few-shot without RAG and with few-shot+RAG on structured notes

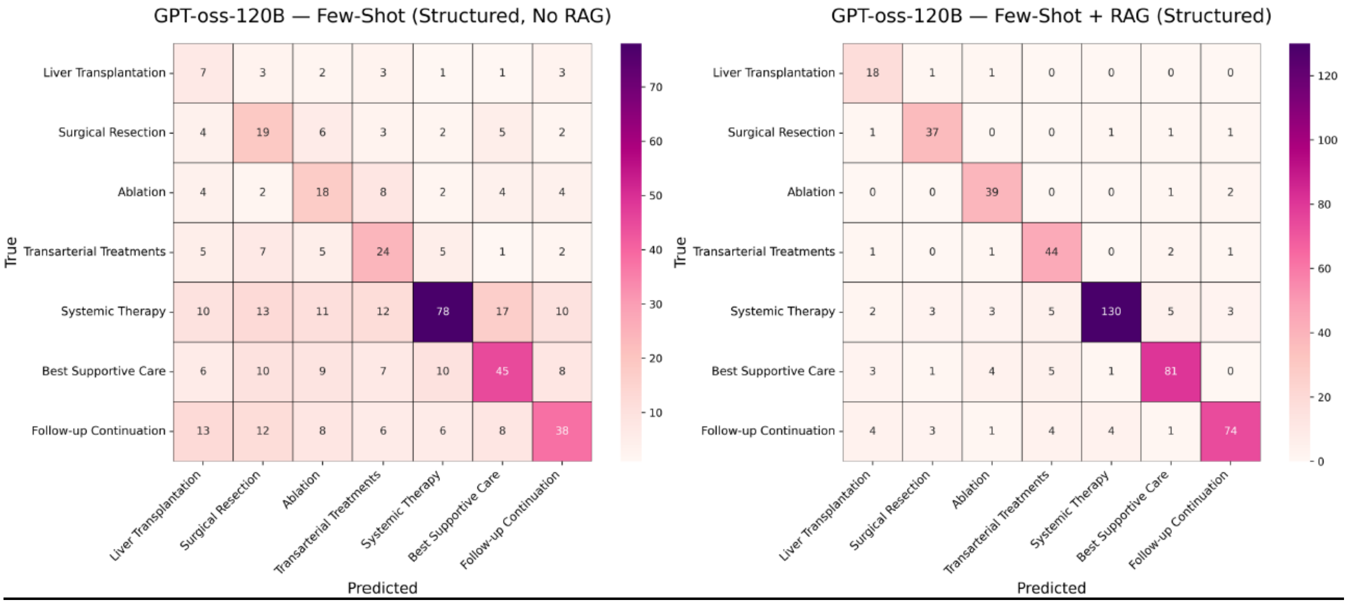

Fig. S2: Confusion matrix for clinical complexity by GPT-oss-120B with few-shot without RAG and with few-shot+RAG on structured notes

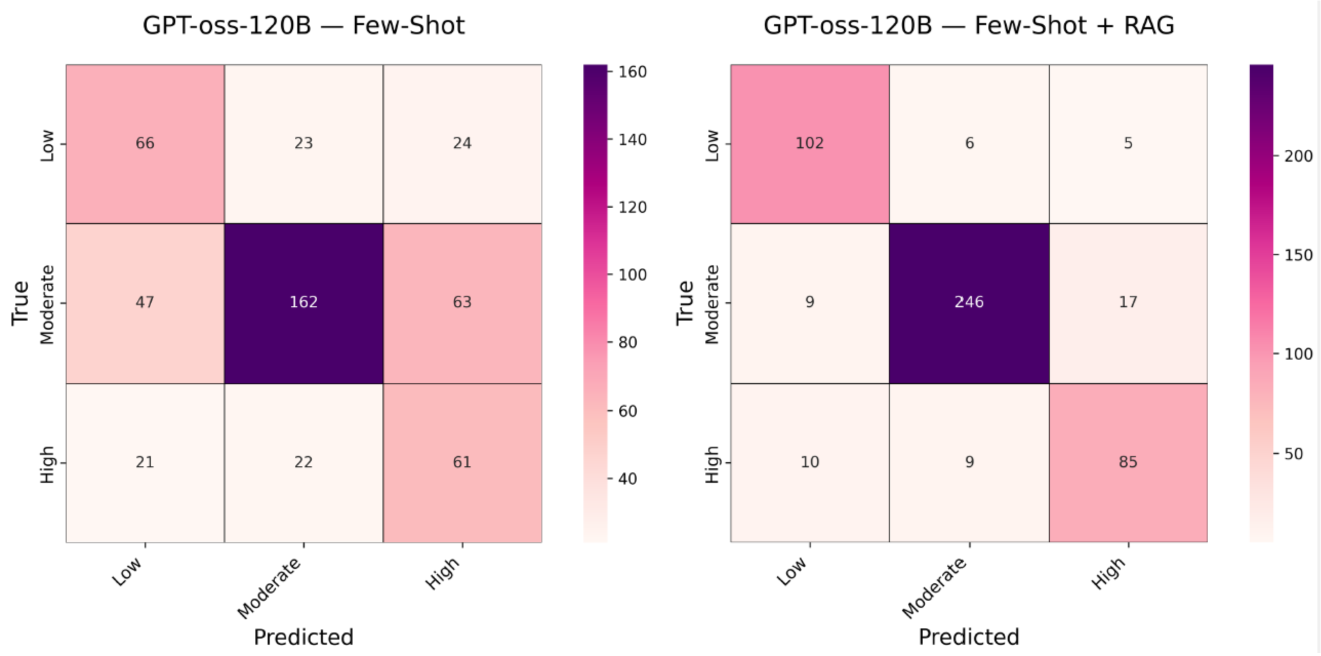

Supplement: Multimedia component 4 [file mmc4.pdf]
